# Responsive Supramolecular Sensors Based on Pillar[5]arene–BTD Complexes for Aqueous Sensing: From Static Quenching to Anion and DNA Recognition

**DOI:** 10.1021/acsomega.5c10011

**Published:** 2025-12-31

**Authors:** Débora Kélen Silva da Conceição, Yasmin Petter daVeiga, Claudiana Dotti, Luis García-Río, Adriana Passarela Gerola, Henrique de Castro Silva Junior, Fabiano Severo Rodembusch, Ricardo Ferreira Affeldt, Angélica Venturini Moro

**Affiliations:** † Laboratory of Molecular Catalysis, 28124 Universidade Federal do Rio Grande do Sul, Porto Alegre, Rio Grande do Sul 90010-150, Brazil; ‡ Laboratory of Catalysis and Interfacial Phenomena, 28117 Universidade Federal de Santa Catarina, Florianópolis, Santa Catarina 88040-900, Brazil; § Departament of Physical Chemistry, 16780 Universidad de Santiago de Compostela, Santiago de Compostela 15782, Spain; ∥ Departamento de Química Fundamental, Instituto de Química, 67825 Universidade Federal Rural do Rio de Janeiro, Rodovia BR-465, Km 7, Seropédica, Rio de Janeiro 23897-000, Brazil

## Abstract

We report the design and investigation of supramolecular guest–host systems based on cationic pillar[5]­arene macrocycles and fluorescent 2,1,3-benzothiadiazole (BTD) derivatives. The BTD guests, obtained through Sonogashira cross-coupling, display intense visible emission, large Stokes shifts, and strong solvatochromism, while the pillar[5]­arene hosts enable selective recognition in aqueous media. Spectrofluorimetric titrations and NMR experiments revealed the formation of 1:1 inclusion complexes between dianionic BTD derivatives and cationic pillar[5]­arenes, with binding constants on the order of 10^5^ M^–1^. Static fluorescence quenching was observed upon complexation, in agreement with molecular docking simulations (Δ*G* ≈ −8.5 kcal mol^–1^) that highlighted the role of electrostatic and π–π interactions. Proof-of-concept sensing studies demonstrated that competitive binding of anions or DNA induces disassembly of the nonemissive BTD **4**⊂P­[5]­A complex, restoring the intrinsic fluorescence of the guest. These results establish pillar[5]­arene–BTD assemblies as promising supramolecular platforms for responsive fluorescence sensing in aqueous environments.

## Graphical abstract

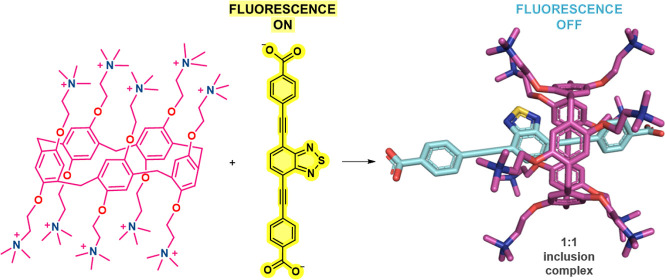

## 1 Introduction

In nature, biochemical processes occur within confined spaces of macromolecular structures, such as cellular organelles or proteins.^1^ These structures, which possess well-defined internal cavities, are commonly referred to as host molecules. The species encapsulated within these hosts are known as guest molecules, and encapsulation relies on the complementarity of size, shape, and chemical surface properties between host and guest.^2^ Natural hosts, such as enzymes, typically display high guest selectivity, leading to the formation of supramolecular assemblies with enhanced stability.^3^

Among synthetic hosts, pillar­[*n*]­arenes have attracted particular attention due to their rigid architecture, ease of functionalization, and diverse self-assembly modes. Multiple noncovalent interactions enable them to selectively bind a wide range of guest molecules and construct versatile supramolecular systems. These interactions also allow pillar­[*n*]­arenes to exhibit tunable responses to external stimuli.^4,5^ As the fifth generation of macrocyclic hosts, following crown ethers, cyclodextrins, calixarenes, and cucurbiturils, pillar­[*n*]­arenes consist of hydroquinone units bridged at the *para* positions by methylene linkers.^6,7^ Despite being the most recently discovered class of major macrocyclic hosts, they have found extensive application in guest–host chemistry, where selective functionalization at the hydroquinone *para* positions imparts solubility in aqueous or organic media broaden their utility.^8–11^

Pillar­[*n*]­arenes and their guest–host complexes have been applied in diverse areas, including self-assembled materials,^12^ stimuli-response sensors,^13^ supramolecular polymers,^14^ drug delivery,^15^ ion separation,^16^ molecular machinery,^17^ artificial transmembrane channels,^18^ catalysis,^19^ and biological recognition.^20,21^ Functionalized pillar­[*n*]­arenes have served as receptors for both cations and anions, with interactions elucidated by spectroscopic methods such as UV–vis and NMR.^22,23^

Depending on the nature of the guest–host interaction, both fluorescence quenching and enhancement have been reported as effective strategies for chemical sensing^24,25^ and for detecting ions and biomolecules in aqueous environments.^26–28^ The combination of synthetic organic fluorophores with pillar­[*n*]­arenes can generate noncovalent assemblies that modulate the photophysical behavior of the free fluorophore, rendering these supramolecular systems responsive to external stimuli, as already reported in the literature.^29–32^ A complementary strategy involves associating fluorescent organic guests with pillar­[*n*]­arenes to create simpler yet efficient systems in aqueous media, where water solubility is readily achieved by introducing charged substituents. For instance, ammonium pillar[5]­arene displays excellent solubility and has been used to develop selective fluorescence-based sensors for phenols and chlorophenols.^33^ Wei and co-workers also reported supramolecular complexes between ammonium pillar[5]­arene and aromatic fluorescent anions that exhibited enhanced emission upon Al^3+^ binding, functioning as “off–on” sensors. This system was further employed in sequential recognition of CN^–^ through competitive coordination, showing reversible “on–off–on” switching with minimal signal loss. Only the assembled complex, not the isolated components, responded to CN^–^, highlighting the critical role of supramolecular assembly.^34–36^

In our recent work, we have employed both neutral and charged pillar­[*n*]­arenes in applications including enzyme-mimicking catalysts,^37,38^ pH-regulated guest–host association,^39^ drug recognition,^40^ and modulation of fluorophore photophysical properties.^41^ Although highly fluorescent 2,1,3-benzothiadiazole (BTD) derivatives have been widely explored in guest–host chemistry,^42–44^ only one example involving pillar­[*n*]­arenes has been reported, in which supramolecular polymers of the BTD dye were assembled within the channels of pillar[5]­arenes.^45^ BTD-based fluorophores are well-known as chemosensors due to their favorable photophysical properties, including high extinction coefficients, intense visible emission, large Stokes shift, and pronounced solvatochromism.^46–51^ Tailored derivatives can emit across a wide spectral range, from blue to red, depending on the environment, with high fluorescence quantum yields.^52–54^ Despite their widespread use, the integration of BTD fluorophores with pillar­[*n*]­arene hosts remains unexplored, representing a promising opportunity for developing new supramolecular architectures. Whereas most BTD derivatives employed in light-emitting technologies follow a donor–acceptor–donor design with electron-rich aromatics directly conjugated to the BTD core,^55–60^ our approach introduces an alkyne spacer between the aryl and BTD units. Furthermore, a carboxylic substituent is incorporated into the aryl ring to extend the conjugated system.

In this context, we synthesized BTD-based fluorophores as guests and investigated their supramolecular association with functionalized pillar­[*n*]­arene hosts. The integration of highly emissive BTD derivatives with pillar­[*n*]­arenes aims to combine the favorable photophysical properties of the guests with the selective binding capabilities of the hosts, thereby enabling the construction of responsive supramolecular systems. By exploring the modulation of fluorescence through guest–host interactions, this study seeks to establish a platform for the development of advanced chemical sensors. The combination of synthetic BTD fluorophores and versatile pillar­[*n*]­arene hosts not only expands the scope of supramolecular chemistry but also opens promising opportunities for applications in environmental monitoring, biological recognition, and other sensing technologies.

## 2 Experimental Section

### 2.1 Materials and Methods

All solvents and reagents (Merck) used in this study were obtained commercially and, unless otherwise noted, were employed without further purification. Triethylamine was distilled and stored over KOH. 1,2-Dichloroethane was distilled over calcium hydride (CaH_2_) and stored over molecular sieve. Analytical grade or absolute solvents (ethanol, dimethylformamide, 1,4-dioxane, ethyl acetate, hexane, dichloromethane, and chloroform) were purchased from Quimidrol, Vetec, Neon, and Sigma-Aldrich. Aqueous experiments were carried out using deionized water (PureLab Option-Q, 18.2 MΩ cm^–1^). Reactions requiring an inert atmosphere were performed under argon by heating followed by purging. Reaction progress was monitored by thin-layer chromatography (TLC) using aluminum-backed silica gel plates, with visualization under UV light or by staining with potassium permanganate or iodine. Products were purified by flash column chromatography on silica gel 60 (230–400 mesh) using appropriate solvent mixtures as eluents. Fourier transform infrared (FTIR) spectra were recorded on a Bruker Alpha spectrometer. Samples were prepared as KBr pellets. Deuterated solvents, including D_2_O, CDCl_3_, CD_3_OD-*d*_4_, and DMSO-*d*_6_ (99.8% + 0.05% TMS), were acquired from Cambridge Isotope Laboratories. ^1^H and ^13^C NMR spectra were recorded on Bruker AVANCE DRX spectrometers (Department of Chemistry, UFSC) operating at 200 or 400 MHz (200 or 400 MHz for ^1^H, 50 or 100 MHz for ^13^C), and on Varian VNMRS spectrometers operating at 400 MHz (400 MHz for ^1^H, 100 MHz for ^13^C), using 5.0 mm sample tubes. Chemical shifts (δ) are reported in parts per million (ppm) relative to tetramethylsilane (TMS, δ = 0.00 ppm) for ^1^H NMR. Residual nondeuterated solvent signals were used as internal references for ^1^H spectra, while deuterated solvent signals were used as references for ^13^C spectra, in accordance with the literature: DMSO-*d*_6_ (δ = 2.50 ppm for ^1^H, 39.5 ppm for ^13^C), D_2_O (δ = 4.79 ppm for ^1^H), CD_3_OD-*d*_4_ (δ = 4.87 ppm for ^1^H, 49.0 ppm for ^13^C), and CDCl_3_ (δ = 7.27 ppm for ^1^H, 77.0 ppm for ^13^C, central triplet). Signal multiplicities are indicated as s (singlet), d (doublet), or t (triplet). Proton assignments were deduced from relative integrations, and coupling constants (*J*) are reported in hertz (Hz).

### 2.2 Synthesis of Guests^47,61^

In a Schlenk flask, the alkyne (1.0 mmol) was dissolved in a 1:1 mixture of DMF and Et_3_N (29 mL) under stirring. The solution was degassed for 20 min, after which PdCl­(PPh_3_)_2_ (26.5 mg, 0.037 mmol), CuI (6.1 mg, 0.032 mmol), and 4,7-dibromo-2,1,3-benzothiadiazole (145 mg, 0.5 mmol), previously prepared according to the literature, was added.^62,63^ The reaction mixture was stirred at 80 °C under an argon atmosphere for 16 h. After cooling to room temperature, the mixture was poured into water (26 mL), resulting in product precipitation. The solid was collected by vacuum filtration with a Büchner funnel and washed sequentially with water, hexane, and a small amount of dichloromethane. The respective crude products BTD **1** or BTD **3** were purified by flash column chromatography using ethyl acetate/hexane (1:9) as the eluent. 4,4′-(2,1,3-Benzothiadiazole-4,7-diyldi-2,1-ethynediyl)­bis­[dimethyl benzoate] (BTD **1**): yield 60% (137 mg). ^1^H NMR (400 MHz, CDCl_3_, ppm): δ 8.01 (d, *J* = 8.5 Hz, 4H), 7.72 (s, 2H), 7.59 (d, *J* = 8.5 Hz, 4H), 3.96 (s, 6H). ^13^C NMR (100 MHz, CDCl_3_, ppm): δ 166.3, 153.0, 132.5, 132.4, 130.6, 129.6, 126.1, 113.9, 81.8, 76.3, 52.4. 4,4′-(2,1,3-Benzothiadiazole-4,7-diyldi-2,1-ethynediyl)­bis­[benzoic acid] (BTD **3**): yield 48% (53 mg). ^1^H NMR (400 MHz, CDCl_3_, ppm): δ 7.78 (s, 2H), 7.66 (dd, *J* = 8.8, 5.4 Hz, 4H), 7.10 (t, *J* = 8.7 Hz, 4H). ^13^C NMR (100 MHz, CDCl_3_, ppm): δ 163.0 (d, *J* = 163.3 Hz), 154.3, 134.0 (d, *J* = 8.5 Hz); 132.4, 118.6, 117.1, 116 (d, *J* = 22.1 Hz), 96.4, 85.0.

BTD **2** was obtained by hydrolysis of BTD **1**, as reported in the literature.^61,64^ In a typical procedure, BTD-ester **1** (86 mg, 0.20 mmol) was dissolved in THF (35 mL) in a round-bottom flask. An aqueous solution of NaOH (80 mg, 2.0 mmol) was then added, and the reaction mixture was refluxed for 24 h. After cooling to room temperature, 2.0 M HCl was added dropwise until the pH reached 2, resulting in product precipitation. The mixture was centrifuged, and the supernatant was discarded. The yellow solid was washed with water and centrifuged three times until the washings reached neutral pH. The solid was then washed with acetone, centrifuged, and dried under high vacuum to afford the desired product. 4,7-Bis­[2-(4-fluorophenyl)­ethynyl]-2,1,3-benzothiadiazole (BTD **2**): yield 86% (73 mg). ^1^H NMR (400 MHz, 40 °C, DMSO-*d*_6_, ppm): δ 8.03 (m, 4H), 8.03 (s, 2H), 7.77 (d, *J* = 7.4 Hz, 4H).

### 2.3 Synthesis of Hosts

Pillar­[5]­arene P­[5]­Im:^37,65^ In a 25 mL round-bottom flask, dimethylformamide (4.0 mL) was added, followed by powdered potassium hydroxide (902 mg, 16 mmol) and imidazole (283 mg, 4.0 mmol). The mixture was stirred for 30 min before addition of previously prepared brominated pillar[5]­arene (P[5]­Br,^37,39^ 564 mg, 0.332 mmol). The volume was then adjusted with DMF (4.4 mL), and the reaction was stirred at room temperature for 24 h. After this period, cold water (25 mL) was added, and the flask was stored in the refrigerator for 8 days, resulting in the formation of a white precipitate. The solid was collected by centrifugation, and the supernatant (DMF/water) was discarded. The precipitate was washed twice with water, centrifuging after each wash, followed by a wash with a 3:1 mixture of water and acetone. The resulting solid was dried under high vacuum to afford the desired product. Yield 59% (307 mg). ^1^H NMR (400 MHz, CDCl_3_, ppm): δ 7.68 (s, 10H), 7.09 (s, 10H), 6.95 (s, 10H), 6.47 (s, 10H), 4.17 (t, *J* = 5.0 Hz, 20H), 3.82 (t, *J* = 5.0 Hz, 20H), 3.55 (s, 10H). ^13^C NMR (100 MHz, CDCl_3_, ppm): δ 149.9, 137.5, 129.3, 127.8, 119.6, 115.7, 68.5, 46.4, 29.0.

Pillar­[5]­arene P[5]­A:^39,65,66^ A solution of previously prepared brominated pillar[5]­arene (P[5]­Br,^37,39^ 292 mg, 0.17 mmol) in ethanol (21 mL) was prepared. To this solution, Me_3_N (45% aqueous solution, 765 mg, 12.94 mmol) was added, and the mixture was refluxed for 24 h. After cooling to room temperature, water (5 mL) was added to dissolve the product. The mixture was filtered through a Büchner funnel, and the aqueous phase was evaporated to afford a dark yellow liquid. Ethanol was then added, inducing crystallization. The suspension was kept in the refrigerator for 1 h, and then filtered. The resulting white solid was dried under high vacuum to yield the desired product. Yield 96% (372 mg). ^1^H NMR (400 MHz, CDCl_3_, ppm): δ 6.83 (s, 10H), 4.34 (s, 20H), 3.81 (s, 10H), 3.69 (s, 20H), 3.10 (s, 90H). ^13^C NMR (100 MHz, CDCl_3_, ppm): δ 149.4, 129.9, 116.5, 64.9, 63.4, 54.0, 29.3.

### 2.4 Photophysics

HPLC-grade solvents were used for steady-state spectrophotometric and spectrofluorimetric measurements. UV–vis absorption spectra were recorded on a Varian Cary 50 spectrophotometer equipped with a Microquímica thermostatic bath (model MQBTC99-20). CaryWinUV 3.00 software was used for data acquisition and processing. Fluorescence spectra were obtained on a Varian Cary Eclipse spectrofluorimeter, equipped with a xenon lamp as the excitation source and variable slit widths and voltages. All experiments were carried out at room temperature in solution at concentrations of 10^–5^–10^–6^ M. The absorption maximum was used as the excitation wavelength for fluorescence emission measurements. Fluorescence quantum yields were determined in diluted solutions with absorbances below 0.1, using coumarin 153 (Φ_FL_ = 0.53) as the quantum yield standard.^67^

### 2.5 Theoretical Calculations

All calculations were performed using the ORCA quantum chemistry package (v 6.1).^68^ Initial molecular geometries were generated from a conformational search using the GOAT module^69^ with the GFN2-xTB semiempirical method.3 ^70^ The resulting global energy minima were then reoptimized for the ground (S_0_) and first singlet excited (S_1_) states at the ωB97X-D4/Def2-TZVP^71–73^ level of theory. Solvent effects (water, EtOH, CH_2_Cl_2_, THF, and 1,4-dioxane) were included in all optimizations via the Conductor-like Polarizable Continuum Model (CPCM).^74^

Vertical electronic excitations were calculated using Time-Dependent Density Functional Theory (TD-DFT).^75^ These calculations employed the SOS-ωPBEPP86 double-hybrid functional with the Def2-TZVP basis set, a method well-suited for describing intramolecular charge-transfer states.^76^ The Tamm-Dancoff Approximation (TDA) was applied,^77^ and the lowest 100 electronic transitions were computed. The character of key excitations was analyzed using Natural Transition Orbitals (NTOs)^78^ and further analysis of the molecular orbitals was conducted with the NBO7 program.^79^

A known caveat of double-hybrid TD-DFT is the systematic overestimation of vertical excitation energies.^76^ To correct for this deviation and facilitate a more direct comparison with experiment, a uniform energy shift (Δ*E*) was applied to the raw calculated TD-DFT energies (*E*_calc_). This empirical shift for this molecular set was determined to be Δ*E* = −0.343 eV, representing the mean difference between the theoretical and experimental electronic transition energies across the 19 available data points. All corrected wavelengths (λ_corr_) reported herein were then obtained by applying this energy shift to the raw theoretical wavelengths (λ_th_) using the relation presented in eq 1

1 $$\lambda_{corr}=\frac{1240}{1240/\lambda_{th}-0.343}$$

### 2.6 Guest–Host Characterization

For the NMR investigation of the guest–host interactions between pillar[5]­arenes and BTD derivatives, stock solutions of P[5]­A (1.0 mM in D_2_O) and BTD (1.0 mM in D_2_O containing 4.0 mM NaOD) were first prepared. Only BTD **4** was employed in these experiments due to its superior solubility in deuterated water, which made it suitable for aqueous studies. Mixtures of P[5]­A and BTD **4** were then combined at molar ratios of 1:0.125, 1:0.25, 1:0.5, 1:0.75, and 1:1. These experiments aimed to verify whether chemical shift variations occurred for the guest protons, which was assessed by comparison with spectra of free BTD **4** and pure P[5]­A. Typically, shifts larger than 1.0 ppm are attributed to the shielding effect exerted by the aromatic rings of the macrocycle on guest protons located inside the cavity. Fluorescence studies were subsequently performed to further characterize the binding. Mixtures of P[5]­A and BTD **4** were prepared directly in the cuvette at the same molar ratios, with final concentrations of P[5]­A adjusted to approximately 1.0 × 10^–5^ M and 1.0 × 10^–6^ M. The choice of the studied systems was guided by solubility and electrostatic considerations: P[5]­A with BTD **4** was selected because electrostatic attraction was expected to promote complexation in aqueous medium, while P[5]­Im with BTD **3** was investigated as a complementary system. The latter derivative had previously shown superior photophysical performance in ethanol, making it a promising candidate for subsequent complexation studies with the neutral P[5]­Im. Fluorimetric titrations were then conducted. In the case of P[5]­A with BTD **4**, sequential additions of 0.05, 0.1, 0.2, 0.3, 0.4, 0.6, 1.0, and 2.0 equiv of P[5]­A were made to a cuvette containing BTD **4** (2.3 × 10^–6^ M), with P[5]­A concentrations ranging from 1.0 × 10^–7^ to 1.0 × 10^–6^ M. Similarly, titration of P[5]­Im with BTD **3** was carried out by adding 0.1, 0.2, 0.3, 0.4, 0.6, 1.0, and 2.0 equiv of P[5]­Im to a cuvette containing BTD **3** (2.7 × 10^–6^ M), with the concentration of P[5]­Im varied between 1.0 × 10^–7^ and 1.0 × 10^–6^ M. Fluorescence spectra were recorded and analyzed using OriginPro 9.0, applying nonlinear fitting to a 1:1 receptor-substrate binding model.

### 2.7 Molecular Docking

Geometry optimization of the BTD ligands was performed using Gaussian09 package (Gaussian, Inc.) for Density Functional Theory at B3LYP 6,31g­(d) basis-set prior to molecular docking simulations.^80^ The investigation of stability and modes of complexation of BTD and P[5]­A were performed with AutoDock4 software (version 4.2.6, Scripps Research Institute)^81^ using previously optimized free sigma-bond rotation models of three different protonation states of BTD as ligands (neutral, monoanionic and dianionic) and rigid pillar[5]­arene model with open portals with +10 charge as macromolecule. The gridbox (100 × 100 × 100) was created with AutoGrid4 ensuring that the macromolecule was centered inside a box covering all atoms. The molecular docking was performed with 1000 scans using Lamarckian genetic algorithm with rmsd tolerance of 2.0 and the different conformations were grouped in energy clusters where the resulting lower energies achieved for inclusion complexes were between 8.15 and 8.79 kcal mol^–1^.

## 3 Results and Discussion

### 3.1 Synthesis

The conjugated BTD derivatives (BTD **1** and BTD **3**) were synthesized through double Sonogashira cross-coupling reactions between dibromo-BTD and terminal alkynes, affording internal alkynes with extended conjugation, using PdCl_2_(PPh_3_)_2_ and CuI as catalysts, DMF as solvent and triethylamine as base (Scheme 1). The acid derivative BTD **2** was obtained by basic hydrolysis of the corresponding ester derivative BTD **1**.

**Scheme 1:**
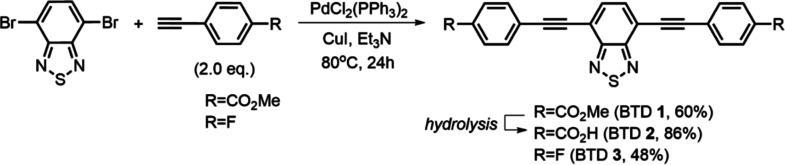
Synthesis of the Guests 4,7-Aryl-substituted Benzothiadiazoles (BTD **1–3**); Hydrolysis: NaOH/H_2_O, THF, 75 °C, 24h, Then HCl 2M to pH = 2

The pillar[5]­arene derivatives P[5]­Im and P[5]­A were obtained by bimolecular nucleophilic substitution (S_N_2) of the brominated pillar[5]­arene P[5]Br with either imidazole or trimethylamine (Scheme 2). Introduction of the imidazole unit in the presence of excess base afforded the pillar[5]­arene-imidazole derivative P[5]­Im in 59% yield, providing a neutral derivative with potential for hydrogen-bonding and π–π interactions.^37^ In contrast, quaternization with excess trimethylamine yielded the cationic pillar[5]­arene derivative P[5]­A in 96% yield,^39,66^ enhancing solubility in polar media and enabling electrostatic interactions with anionic guests.

**Scheme 2:**
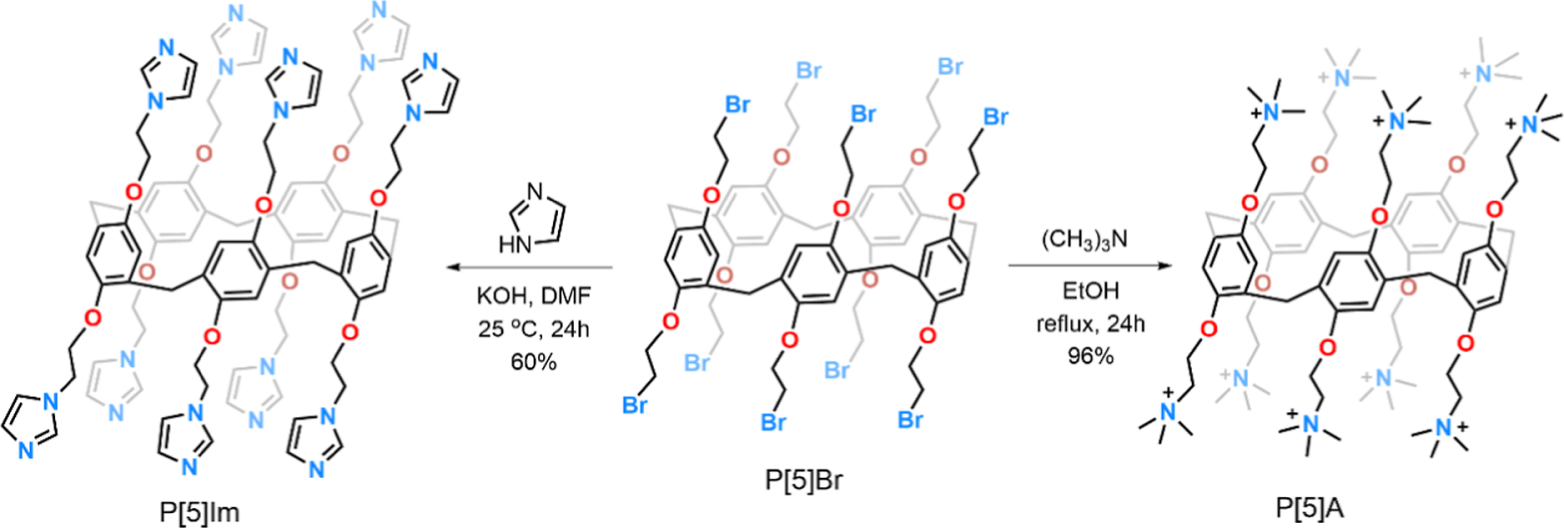
Synthesis of the Hosts Pillar[5]­arenes P[5]­Im and P[5]­A

### 3.2 Photophysical Characterization

Photophysical measurements were performed in solvents of different polarity to assess how the environment influences the electronic behavior of the BTD derivatives. The absorption and emission characteristics, summarized in Figures 1, 2, Tables 1 and 2, reveal the expected solvatochromic behavior of these fluorophores. The dianionic derivative BTD **4** was obtained by deprotonation of BTD **2** with NaOH (pH 10). The introduction of additional negative charges was crucial for enabling supramolecular recognition with the cationic pillar[5]­arene P[5]­A, and its photophysical properties were therefore characterized prior to the complexation studies. The absorption maxima showed only minor variations among the BTD derivatives (BTD **1**: 407–411 nm; BTD **2**: 409–417 nm; BTD **3**: 408–412 nm; BTD **4**: 410–421 nm; Table 1). All compounds displayed similar violet-region bands with molar extinction coefficients on the order of 10^4^ M^–1^ cm^–1^, typical of π–π* transitions in conjugated systems. Strickler–Berg analysis confirmed spin- and symmetry-allowed π–π* excitations.^82,83^ Measurements for BTD **4** in dichloromethane were not possible due to solubility limitations. UV–vis data for the pillar[5]­arene hosts are provided in the Supporting Information. Both P[5]­Im and P[5]­A exhibit absorption maxima near 291 nm, characteristic of π–π* transitions, with no significant solvent dependence observed.

**Figure 1:**
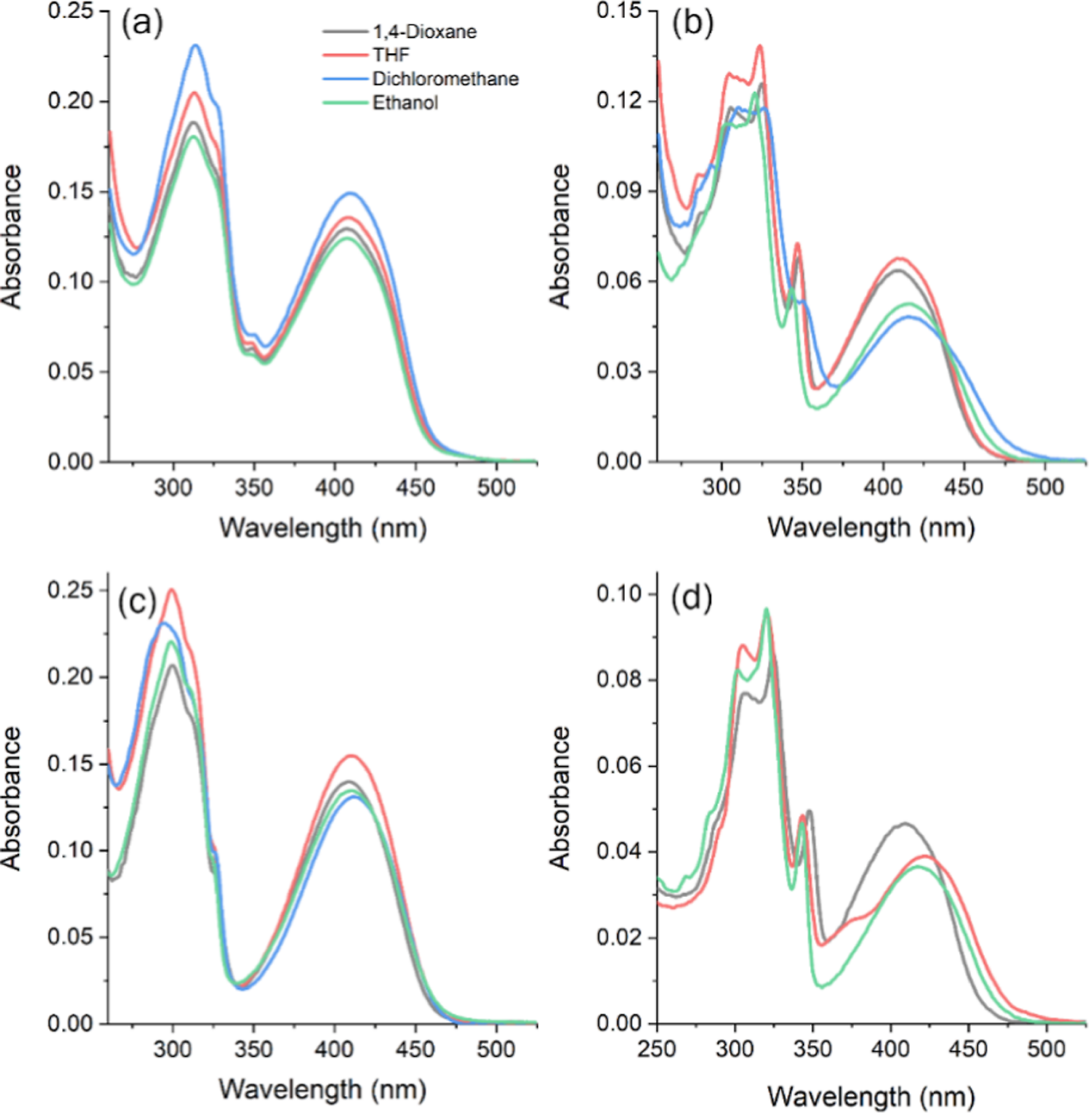
UV–vis absorption spectra in solution [∼10^–5^ M] of BTD **1** (a), BTD **2** (b), BTD **3** (c), BTD **4** (d), in different organic solvents.

**Figure 2:**
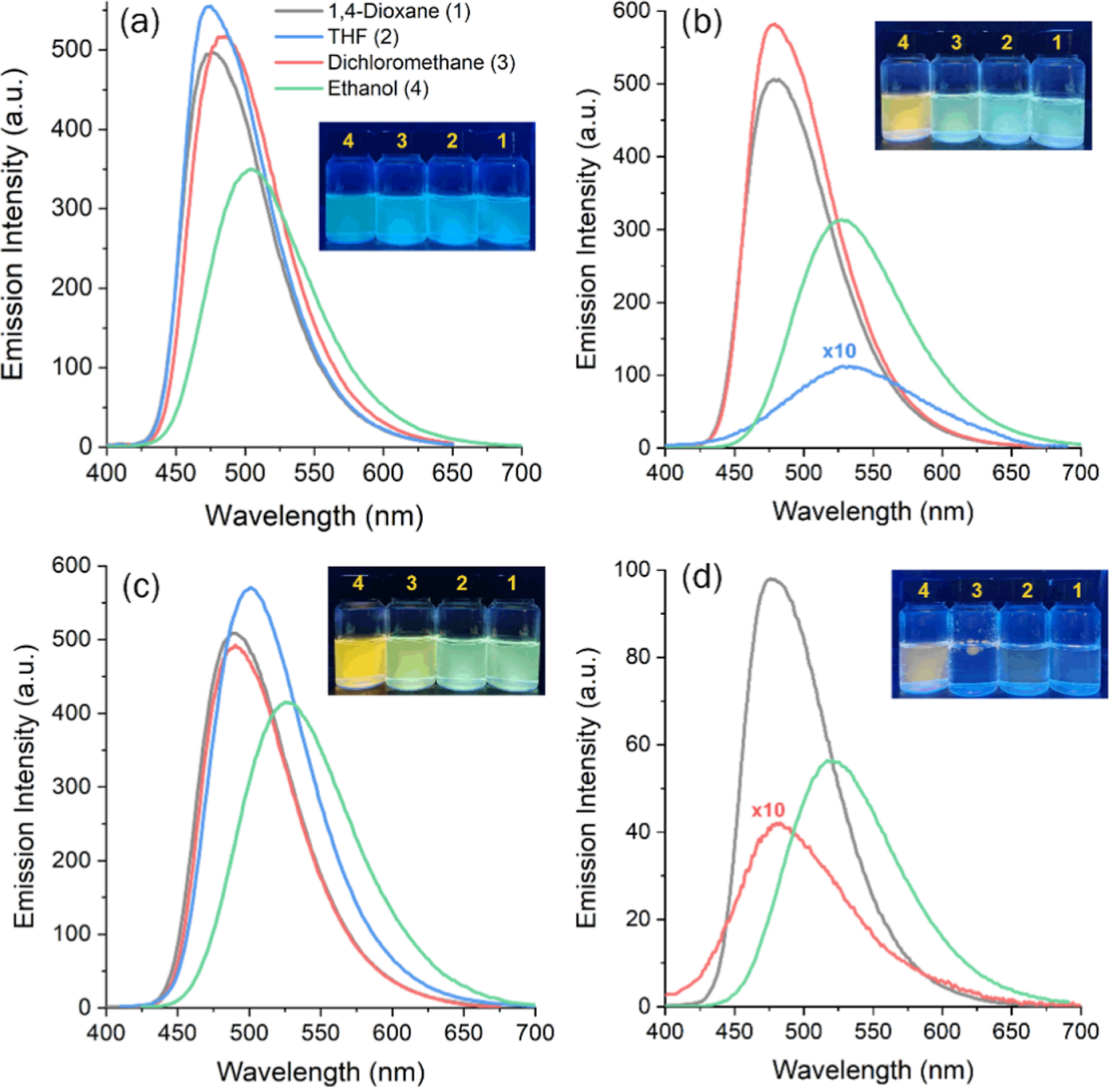
Steady-state fluorescence emission spectra in solution (∼10^–5^ M, excitation/emission slits: 5.0 nm/5.0 nm) of BTD **1** (a), BTD **2** (b), BTD **3** (c), BTD **4** (d), in different organic solvents. The inset are photographs from the respective solutions under UV light (365 nm).

**Table 1:** Ground-State Photophysical Data of BTDs **1–4** in Different Solvents, Where λ_abs_ Is the Absorption Maximum (nm); ε is the Molar Absorptivity (×10^4^ M^–1^ cm^–1^), *f*_e_ Is the Calculated Oscillator Strength, and *k*_e_^0^ is the Calculated Radiative Decay Rate Constant (×10^8^ s^–1^)

BTD	solvent	λ_abs_	ε	*f*_e_	*k*_e_^0^
**1**	1,4-dioxane	408	1.16	0.21	1.29
	THF	410	1.30	0.23	1.39
	CH_2_Cl_2_	411	1.46	0.27	1.59
	ethanol	407	0.59	0.09	0.55
**2**	1,4-dioxane	410	0.58	0.10	0.58
	THF	409	0.61	0.10	0.59
	CH_2_Cl_2_	417	0.48	0.07	0.44
	ethanol	417	0.47	0.08	0.48
**3**	1,4-dioxane	408	0.98	0.19	1.12
	THF	411	0.98	0.21	1.27
	CH_2_Cl_2_	412	1.00	0.23	1.36
	ethanol	410	1.12	0.20	1.18
**4**	1,4-dioxane	410	0.14	0.03	0.20
	THF	421	0.14	0.03	0.16
	CH_2_Cl_2_	-	-	-	-
	ethanol	421	0.16	0.03	0.16

**Table 2:** Excited-State Photophysical Data of BTDs **1–4** in Different Solvents, Where λ_em_ is the Emission Maximum (nm); Δλ_ST_ Is the Stokes Shift (cm^–1^), and Φ_FL_ Is the Relative Fluorescence Quantum Yield

BTD	solvent	λ_em_	Δλ_ST_	Φ_FL_
**1**	1,4-dioxane	476	3500	0.20
	THF	473	3250	0.19
	CH_2_Cl_2_	486	3750	0.40
	ethanol	504	4730	0.96
**2**	1,4-dioxane	480	3560	0.64
	THF	477	3490	0.80
	CH_2_Cl_2_	533	5220	0.69
	ethanol	528	5040	0.85
**3**	1,4-dioxane	490	4100	0.90
	THF	490	3920	0.67
	CH_2_Cl_2_	501	4310	0.68
	ethanol	528	5450	0.92
**4**	1,4-dioxane	479	3510	0.92
	THF	479	2880	0.80
	CH_2_Cl_2_	-	-	-
	ethanol	522	4600	0.82

The emission properties of the BTD derivatives were strongly influenced by solvent polarity and substituent effects. BTD **1** exhibited the widest solvatochromic shift (473 to 504 nm, Δλ_ST_ = 3250–4730 cm^–1^) and the highest efficiency in ethanol (Φ_FL_ = 0.96). Similar trends were observed for BTD **2** (477–533 nm, Φ_FL_ = 0.80–0.85) and BTD **3** (490–528 nm, Φ_FL_ = 0.90–0.92), which showed large Stokes shifts (up to 5450 cm^–1^) attributed to stabilization of the excited state by the fluorine substituent. The dianionic BTD **4** combined high solubility with robust emission (479–522 nm, Δλ_ST_ = 2880–4600 cm^–1^, and Φ_FL_ = 0.80–0.92) in both organic and aqueous media, making it the most versatile fluorophore in the series.

The photophysical properties of the compounds were also investigated in aqueous media (Figure 3). Although limited solubility was expected for some derivatives, these measurements were performed to qualitatively assess their aqueous behavior, relevant for subsequent guest–host studies. BTD **1–3** exhibited pronounced hypochromic effects, while the dianionic BTD **4** showed only a slight decrease in intensity, consistent with its higher solubility. The absorption maxima (398–415 nm) were similar to those in organic solvents, with apparent molar absorptivity values of ∼10^4^ M^–1^ cm^–1^. All derivatives displayed weaker emission and lower quantum yields in water due to nonradiative decay and solubility limitations, yet large Stokes shifts (∼5800–7100 cm^–1^) indicated significant excited-state reorganization. The dianionic BTD **4** maintained measurable fluorescence (λ_em_ ∼545 nm), confirming its potential for supramolecular studies in aqueous media.

**Figure 3:**
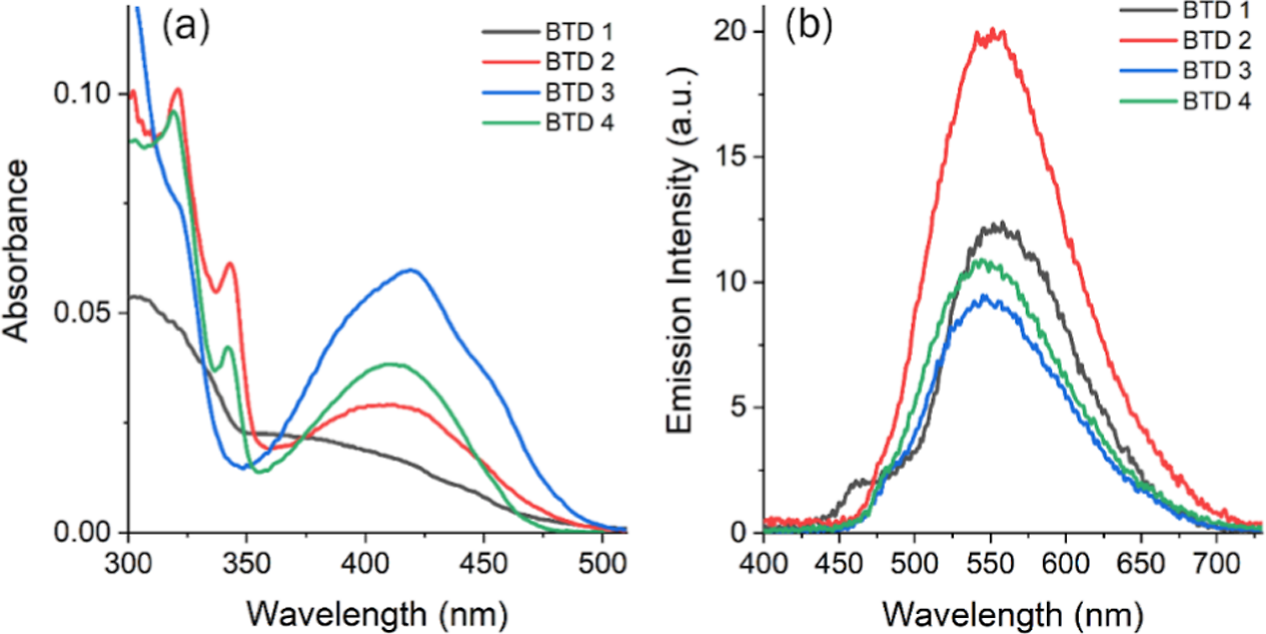
(a) UV–vis and (b) steady-state fluorescence emission spectra of BTD **1–4** in aqueous solutions (∼10^–5^ M).

### 3.3 Theoretical Calculations

The equilibrium geometries of BTDs **1–4** were optimized in both the in the ground (S_0_) and first singlet excited (S_1_) states using the CPCM solvation model in five solvents (water, EtOH, CH_2_Cl_2_, THF, and 1,4-dioxane). Structural variations were quantified as heavy-atom RMSDs after optimal Kabsch alignment,^84^ allowing assessment of solvent-induced distortions and excited-state reorganizations. In this investigation two analyses were conducted: (i) solvent-dependent drift of S_0_ geometries relative to S_0_ (water), and (ii) S_1_ to S_0_ reorganization within each solvent. This distinction isolates dielectric effects on the ground-state minimum from intrinsic excited-state relaxation. Ground-state geometries were nearly solvent-invariant for BTDs **3** and **4** (mean drifts ∼0.02 Å), moderately sensitive for BTD **2** (∼0.08 Å), and more dependent on solvent for BTD **1** (∼0.14 Å) (Table 3). Excited-state reorganizations followed a similar trend: large for BTD **1** and BTD **4** (∼0.42–0.48 Å), moderate for BTD **2** (∼0.12 Å), and minimal for BTD **3** (∼0.05 Å). The nearly solvent-independent displacement of BTD **4** in the S_1_ state suggests a dominant internal relaxation coordinate weakly affected by dielectric screening, whereas BTD **3** remains the most rigid across states and solvents. These results indicate that BTD **4** experiences pronounced yet solvent-stable relaxation, consistent with its strong but environment-insensitive fluorescence response.

**Table 3:** RMSD Values (Å) Comparing the Optimized Ground State (S_0_) Geometries in Various Solvents Against the Reference S_0_ Geometry in Water

	solvent			
BTD	1,4-dioxane	THF	CH_2_Cl_2_	ethanol
**1**	0.139	0.144	0.144	0.146
**2**	0.097	0.078	0.077	0.074
**3**	0.037	0.011	0.010	0.002
**4**	0.043	0.017	0.014	0.007

A qualitative analysis of the Frontier molecular orbitals reveals a highly conserved electronic structure across the BTD series (Figure 4). The HOMO is a π-orbital delocalized along the conjugated framework, with contributions from both the central BTD core and the peripheral aromatic rings, whereas the LUMO is a π* orbital primarily localized on the electron-accepting BTD core. This conserved orbital topology accounts for the uniformity observed in the calculated HOMO–LUMO gaps for all derivatives. To evaluate the effect of photoexcitation and solvent polarity, the HOMO and LUMO energies were analyzed in both the ground (S_0_) and first singlet excited (S_1_) states. The results reveal a pronounced and nearly uniform contraction of the HOMO–LUMO gap upon relaxation to the S_1_ geometry (Δ*E* = −0.883 ± 0.025 eV) (Figure 4). This narrowing arises from simultaneous HOMO destabilization (+0.438 ± 0.011 eV) and LUMO stabilization (−0.446 ± 0.018 eV), indicating a concerted reorganization of the electronic density following excitation. Notably, the magnitude of this effect remains consistent across all BTD derivatives and solvents, underscoring the intrinsic electronic robustness of the series. Full orbital energy data and solvent-resolved results are available in the Supporting Information.

**Figure 4:**
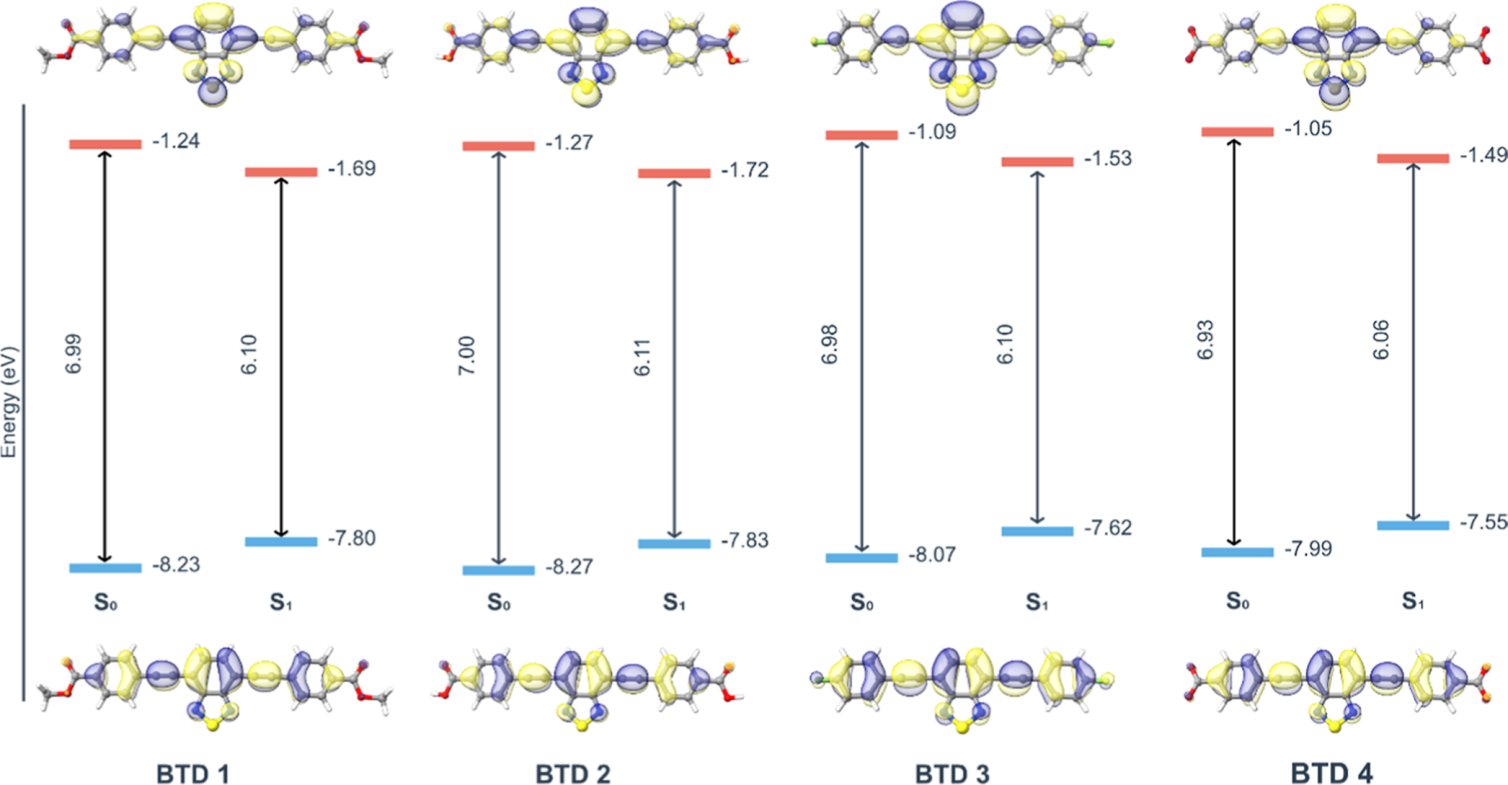
Average HOMO–LUMO gaps (eV) across five solvents (CPCM). The HOMO (bottom) and LUMO (top) are rendered with ρ = 0.03.

Solvent effects, although modest, are discernible in both electronic states. In the ground state (S_0_), polar solvents slightly increase the HOMO–LUMO gap, whereas in the excited state (S_1_) the opposite trend is observed, with water and ethanol producing marginally smaller gaps (Supporting Information). This inversion suggests that the S_1_ state has a larger dipole moment or higher polarizability than S_0_, resulting in preferential stabilization in polar media. Overall, the Frontier orbital energetics are highly consistent across BTDs **1–4**: both absolute gaps and the magnitude of the S_0_ → S_1_ contraction are nearly identical, confirming that the BTD core dictates the electronic structure while peripheral substituents exert negligible influence on the fundamental orbital energies (see Table 4).

**Table 4:** RMSD (S_1_ → S_0_) Values (Å) Comparing the Optimized Excited State (S_1_) Geometries With the Ground State (S_0_) Geometry in Each Solvent

	solvent				
BTD	1,4-dioxane	THF	CH_2_Cl_2_	ethanol	water
**1**	0.438	0.436	0.443	0.430	0.367
**2**	0.087	0.132	0.134	0.135	0.100
**3**	0.045	0.051	0.053	0.055	0.055
**4**	0.475	0.488	0.470	0.490	0.492

To provide a theoretical framework for the experimental absorption spectra, vertical electronic transitions were computed from the optimized S_0_ geometries. In all derivatives, the dominant excitation corresponds to an intense HOMO → LUMO transition with π → π* and intramolecular charge-transfer (ICT) character. Electron density difference (EDD) maps (Figure 5) illustrate the process, showing charge depletion over conjugated backbone and accumulation on the electron-accepting BTD core. This conserved ICT character explains the large Stokes shifts and solvatochromic fluorescence observed experimentally. Quantitative data (Table 5) indicate that the transition involves >90% HOMO → LUMO contribution, with oscillator strengths (*f*_osc_ >1.3) consistent with the high molar extinction coefficients measured experimentally. Although the calculations systematically overestimate excitation energies, applying a uniform correction of −0.343 eV yields excellent agreement with experiment (RMSE = 6.4 nm). The computed absorption spectra predict a slight negative solvatochromism (blue shift) due to preferential stabilization of the ground state in polar media, in agreement with the experimentally observed fluorescence red shift that confirms the higher polarity of the relaxed S_1_ state.

**Figure 5:**
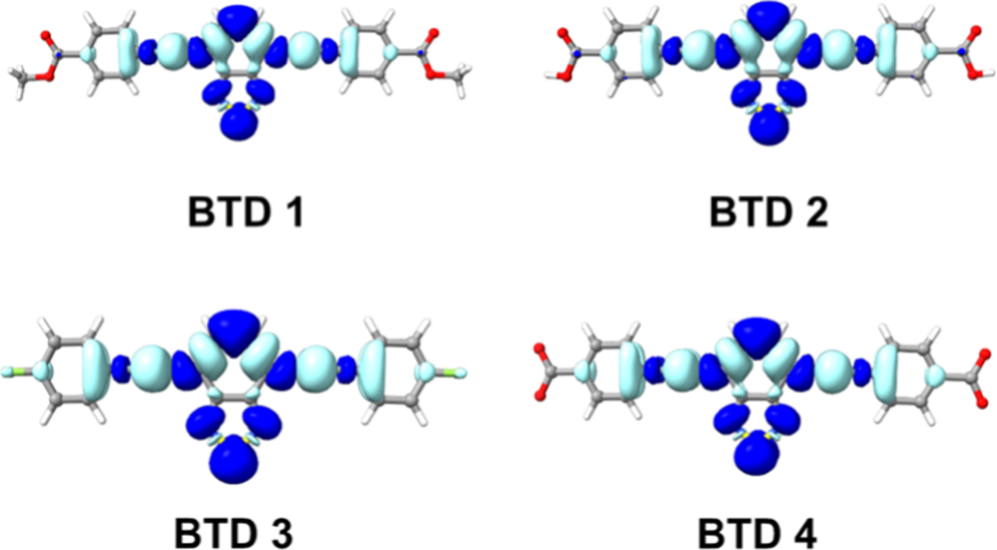
Representative electron density difference (EDD) plots for the S_0_ → S_1_ transition of BTDs **1–4**. Light blue isosurfaces show regions of density depletion (hole), while dark blue shows regions of density accumulation (particle). The selected ρ is 0.0005.

**Table 5:** Calculated and Experimental Electronic Transition Data for the Main Absorption Band of BTDs **1–4**, Where λ_th_ are the Raw TDDFT Wavelengths (nm), λ_corr_ are Corrected by Systematic Energy Shifts (nm) and λ_exp_ are Obtained From Experiments (nm)

BTD	solvent	λ_th_	λ_corr_	*f*_osc_	HOMO → LUMO (%)	λ_exp_
**1**	1,4-dioxane	370.5	410.8	1.71	90.4	408
	THF	368.6	410.7	1.72	90.3	410
	CH_2_Cl_2_	368.8	411	1.72	90.3	411
	ethanol	367.3	408.5	1.72	90.2	407
	water	368.4	406.8	1.63	90.6	398
**2**	1,4-dioxane	370.4	410.7	1.71	90.3	410
	THF	368.1	411.5	1.71	90.2	409
	CH_2_Cl_2_	369.3	412	1.71	90.2	417
	ethanol	367.9	409.5	1.71	90.1	417
	water	365.9	406.4	1.7	90.1	411
**3**	1,4-dioxane	376.5	415.6	1.35	92.5	408
	THF	372.3	415.8	1.34	92.5	411
	CH_2_Cl_2_	372.3	415.8	1.34	92.5	412
	ethanol	370.3	413.2	1.33	92.5	410
	water	369.4	412.1	1.32	92.4	415
**4**	1,4-dioxane	369.7	409.7	1.57	91.5	410
	THF	368.1	410	1.58	91.3	421
	CH_2_Cl_2_	368.4	410.5	1.58	91.3	-
	ethanol	366.9	408	1.58	91.2	421
	water	365.9	406.4	1.58	91.2	412

### 3.4 Guest–Host Interaction Study

The interaction study was first performed with neutral compounds by conducting spectrofluorimetric titrations in ethanol, incrementally adding P[5]­Im to solutions of BTD **1–3**. As illustrated in Figure 6a, using BTD **3**⊂P­[5]­Im complex as a representative model (full data available in the Supporting Information), only minor changes in fluorescence emission intensity were detected, and the correlation with pillar[5]­arene concentration (10^–6^–10^–5^ M) was weak. Monitoring the titration by exciting at the P[5]­Im absorption maximum (λ_exc_ = 291 nm) produced a distinct emission profile when large amounts of the macrocycle were present (1.0–2.0 equiv) (Figure 6b). The observed blue-shifted and enhanced emission of P[5]­Im in the presence of BTD is likely attributed to restricted rotational and vibrational motion of the imidazole groups at the pillar[5]­arene portals upon increasing concentration. This restriction reduces nonradiative relaxation pathways and enhances radiative decay efficiency, leading to stronger emission. At the same time, the resulting intermolecular interactions between macrocycles may hinder guest access and prevent the formation of a stable inclusion complex, an energetically unfavorable outcome.

**Figure 6:**
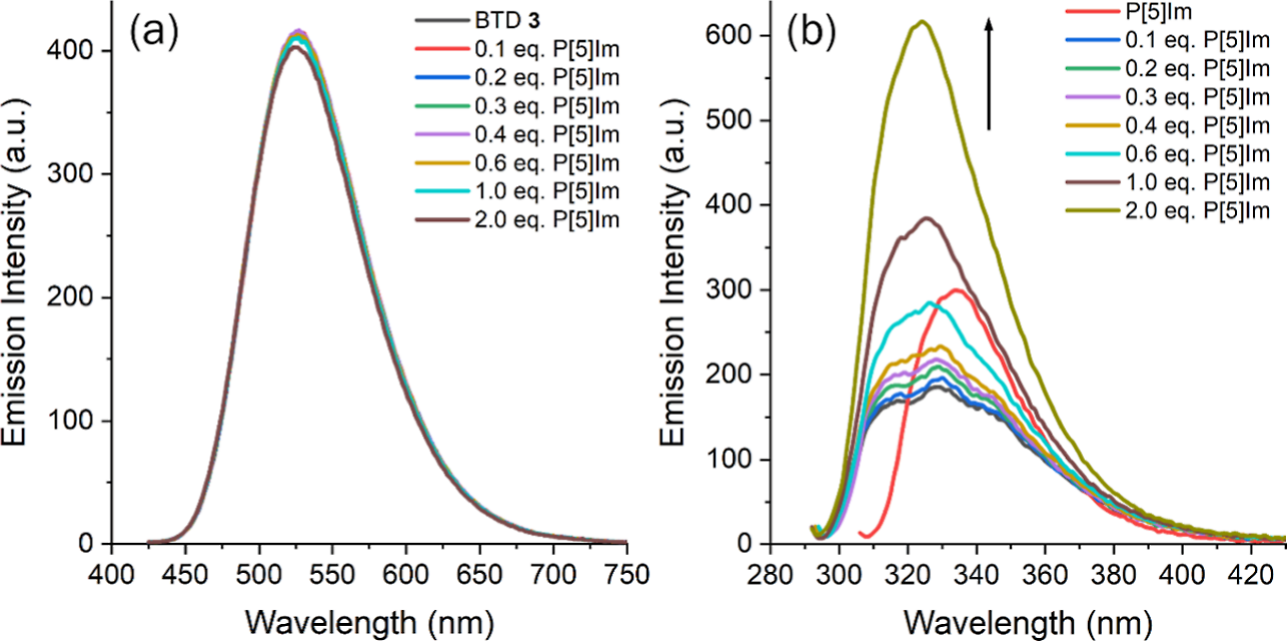
Spectrofluorimetric titration of BTD **3** with different concentrations P[5]­Im (10^–7^–10^–6^ M, 0.1–2.0 equiv) at different excitation wavelengths (a) λ_exc_ = 410 nm and (b) λ_exc_ = 291 nm (excitation/emission slits: 5.0 nm/5.0 nm); P[5]­Im refers to its emission in the absence of BTD **3**.

Given the complex behavior of the neutral system composed of the imidazole-functionalized pillar[5]­arene P[5]­Im and BTD fluorophores, we next investigated charged guest–host pairs by performing spectrofluorimetric titrations of anionic BTD **4** with P[5]­A in water. The addition of 0.1–1.0 equiv of P[5]­A induced pronounced quenching of BTD fluorescence. Mixtures were prepared at different BTD/P[5]­A ratios (1:1, 1:0.75, 1:0.5, 1:0.25, and 1:0.125) at two final concentrations (10^–5^ and 10^–6^ M). Figure 7 displays the fluorescence emission spectra of BTD **4** with P[5]­A in water at 10^–5^ M; the corresponding data at 10^–6^ M are provided in the Supporting Information In both cases, a marked decrease in BTD emission intensity was observed, consistent with supramolecular complex formation between free BTD **4** and P[5]­A, most likely mediated by ionic interactions. Notably, all fluorescence measurements were recorded after 1 h of incubation at room temperature. Nonlinear fittings of the maximum fluorescence intensity as a function of BTD **4** concentration to a 1:1 binding model using the Langmuir equation (Supporting Information), yielded a high binding constant (*K*_b_ = 4.82 × 10^5^ M^–1^).^85,86^

**Figure 7:**
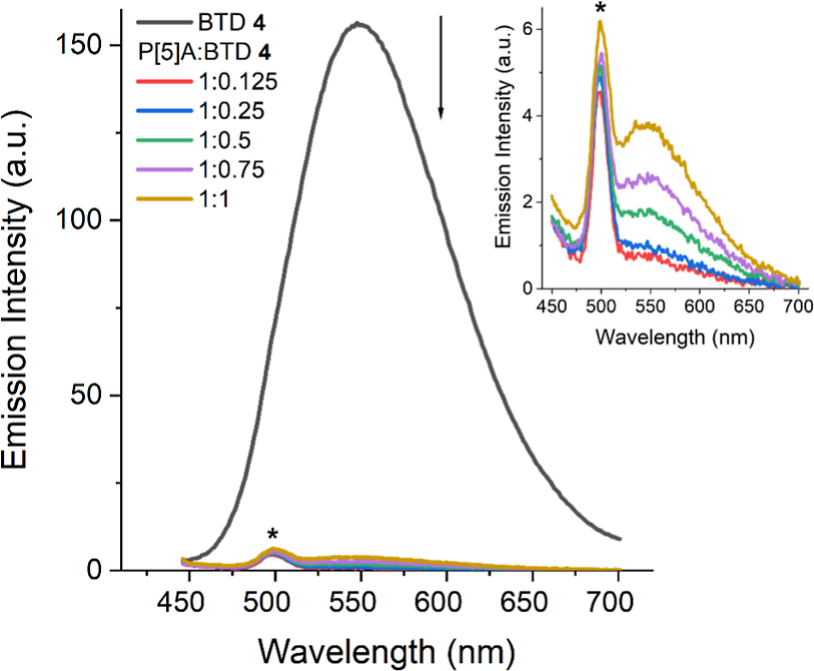
Fluorescence emission spectra for anionic BTD **4** and different mixtures of cationic P[5]­A in water (10^–6^ M), λ_exc_ = 428 nm, excitation/emission slits: 10.0 nm/10.0 nm. The inset magnifies the curves from the P[5]­A/BTD **4**. The asterisk indicated the Raman band.

It is worth noting that the 1 h incubation time and the 10^–6^ M concentration were carefully selected based on spectroscopic studies of a 1:1 mixture of dianionic BTD **4** and P[5]­A in water over different time intervals. We monitored the characteristic UV absorption wavelengths of both chromophores (BTD and pillar[5]­arene) and followed the signal evolution for up to 300 min (Figure 8a). While free BTD **4** exhibited no significant changes during this period, the 1:1 complex displayed a marked decrease in intensity within the first 20 min (Figure 8b, red arrow). The mixing of BTD and pillararene in water required approximately 40 s prior to the first acquisition, with a final concentration of ca. 10^–6^ M. The same experiments performed at 10^–5^ M revealed a detectable decrease in absorption within the first 50 min, which continued to decline throughout the monitored period of 17 h (Supporting Information).

**Figure 8:**
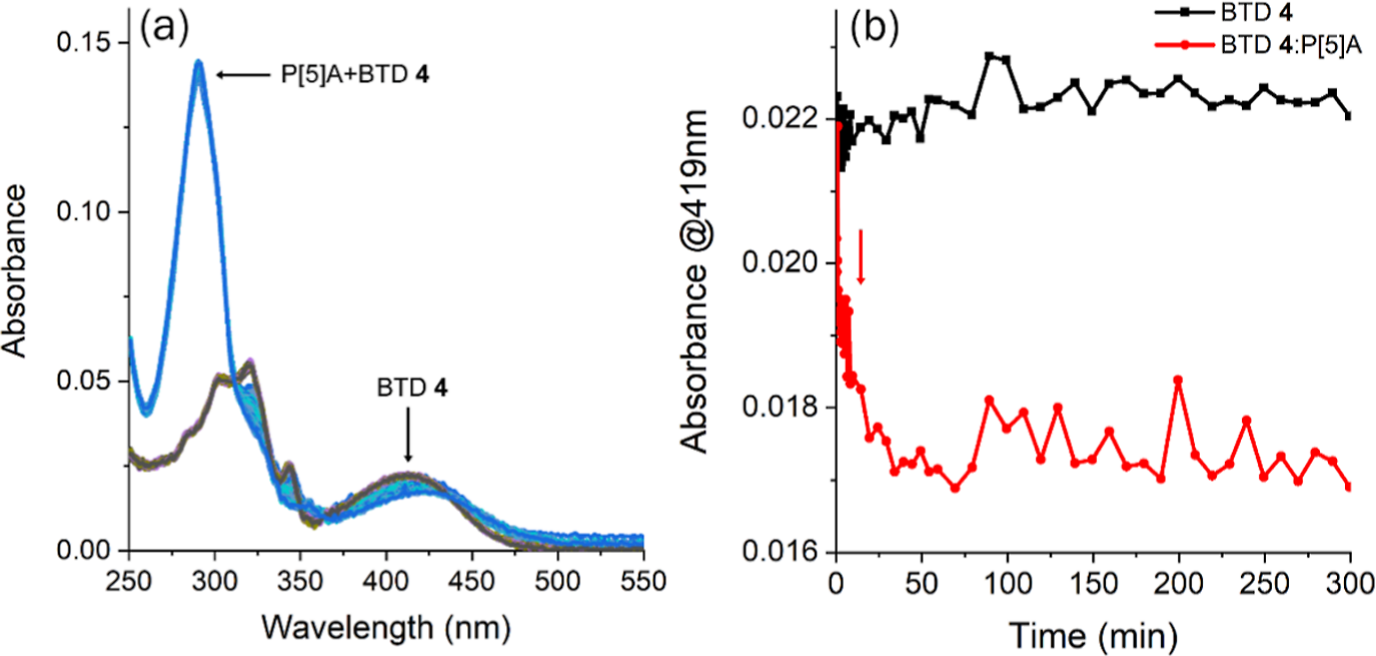
(a) UV–vis absorption spectra of a 1:1 mixture of BTD **4**/P­[5]­A (10^–5^ M) monitored over 300 min. (b) Time-dependent absorbance changes at 291 nm (black) and 419 nm (red).

Based on these results, all fluorescence emission samples were prepared and analyzed after 1 h of incubation at the same concentrations (10^–5^ and 10^–6^ M). Figure 9a shows the emission spectrum of the pure anionic BTD **4** in water, with λ_em_ = 552 nm and an emission intensity of 741 au, while Figure 9b displays the corresponding spectra monitored for 20 min after addition of P[5]­A (10^–5^ M). Mixing the 1:1 solution for approximately 40 s already resulted in a pronounced decrease in fluorescence intensity, with the first measurement (*t* = 0 s) quenching ∼80% of the initial emission (144 au). The rate constant of fluorescence quenching was determined to be 0.46 min^–1^. The same host, P[5]­A, was previously investigated in the presence of monoanionic and dianionic guests, and it was demonstrated that complexation occurs through a two-step process: an initial guest approach governed by electrostatic forces, followed by formal inclusion within the hydrophobic cavity of P[5]­A, the latter being the rate-determining step.^48,87^ The present results are consistent with this mechanism, as the rapid initial decrease if fluorescence intensity upon mixing reflects the fast electrostatically driven association step.

**Figure 9:**
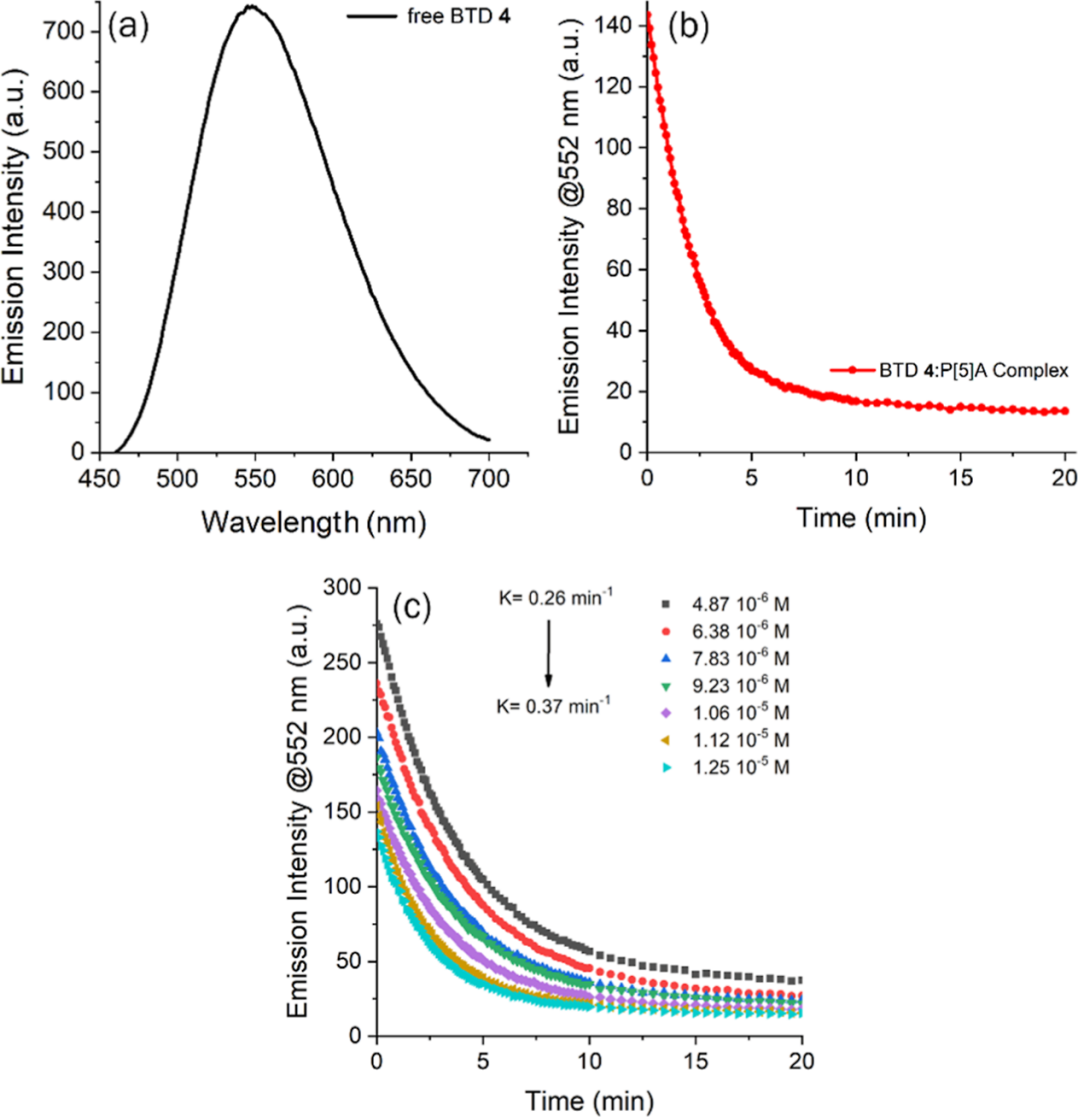
(a) Fluorescence emission spectrum of pure anionic BTD **4** (10^–5^ M, excitation/emission slits: 10.0 nm/10.0 nm). Time-dependent emission intensity at 552 nm for (b) 1:1 mixture of BTD **4** and P[5]­A (10^–5^ M) and (c) BTD **4** (10^–5^ M) and different concentrations of P[5]­A (excitation/emission slits: 10.0 nm/10.0 nm) monitored over 20 min.

Previous studies on inclusion complex formation between P[5]­A host and charged compounds have shown that bromide counterions can influence complexation through ion-exchange processes. Cationic pillar[5]­arene is prone to bind bromide ions, thereby reducing its effective positive charge and consequently its capacity to interact with organic anionic guest. García-Rio and co-workers reported that the binding profile of trimethylammonium pillar[5]­arene with anionic guests strongly depends on its effective positive charge. Based on this, we chose to work at concentrations below 10^–5^ M to maximize the availability of positive charges at the macrocycle portals. At lower concentrations, the effective charge is higher, which facilitates interaction with anionic guests; conversely, increasing concentration decreases the net positive charge from approximately +10 (<10^–5^ M) to +5 (>10^–3^ M).^88^ To support this rationale, a kinetic study was carried out at different concentrations of P[5]­A (10^–6^–10^–5^ M), with the first data points acquired 12 s after mixing (Supporting Information). As shown in Figure 9c, the rate constant increased as the concentration decreased (*k* = 0.26 min^–1^ to *k* = 0.37 min^–1^), consistent with the expectation that lower concentrations enhance accessibility of the host. Under these conditions, slower fluorescence quenching was observed, corresponding to inclusion complex formation (full spectra available in the Supporting Information). Reducing the counterion effect increases the availability of positive charges on P[5]­A to interact with dianionic BTD **4**, thereby promoting guest incorporation.^89^ Since addition of stoichiometric amounts of the macrocycle led to complete quenching of BTD fluorescence, we further investigated the effect of gradually increasing P[5]­A concentration from 10^–7^ to 10^–6^ M and compared the results with free BTD in solution. The results can be rationalized by plotting the free fluorophore emission intensity against the fluorescence emission intensity of each sample in the presence of the quencher (*I*_0_/*I*) as a function quencher concentration, thereby obtaining the Stern–Volmer parameters associated with the type of quenching (Supporting Information). This analysis revealed a static fluorescence quenching (κ_q_ ∼10^14^ M^–1^ s^–1^), as determined by correlating the Stern–Volmer constant (*K*_sv_) with the excited-state lifetime (τ^0^).^90–92^ Static fluorescence quenching is well-known to occur when the emission intensity decreases due to a reduction in the number of available emitting species. In this case, quenching arises in the ground state through complex formation, rather than from diffusion-controlled interactions between excited and emitting species, leading to the generation of new nonemissive complex.

NMR spectroscopy is widely employed for structural elucidation of inclusion complexes and for probing intramolecular interactions through the evaluation of complexation-induced chemical shifts.^93^ Upon mixing different concentrations of anionic BTD **4** with cationic pillar[5]­arene P[5]­A, clear shifts in the BTD hydrogen signals were observed relative to the free BTD (Figure 10). The symmetrically distributed hydrogens from the central core at positions 5 and 6 (green hydrogen) exhibited a chemical shift of −1.03 ppm [Δδ = δ­(complex) – δ­(free)], consistent with the shielding effect arising from inclusion of BTD within the macrocyclic cavity.^94–97^ A similar trend was observed for the hydrogens on the *para*-substituted rings of BTD (orange and blue hydrogens), which showed a shift of −1.13 ppm, attributed to the anisotropic shielding effect of the pillar[5]­arene aromatic rings. In contrast, no significant shifts were detected for the pillar[5]­arene aromatic hydrogens (black hydrogen). No noticeable changes were detected in the aliphatic region of the P[5]­A proton signals during titration with BTD 4, consistent with the interaction occurring mainly through electrostatic and aromatic contacts between the host and guest. Based on these results, Figure 10b depicts a proposed 1:1 interaction between BTD **4** and P[5]­A. Although ^1^H NMR titration of BTD **4**⊂P­[5]­A with NaBr could provide additional evidence for the displacement mechanism, such experiments were not feasible due to the limited solubility of the complex under conditions comparable to those employed in the UV–vis titration studies. Although we recognize the relevance of Job’s plots for evaluating binding stoichiometries, this method was not applied here due to its known limitations in accurately describing supramolecular equilibria, particularly in weakly bound or multistep systems.^98^ Instead, the 1:1 stoichiometry was established based on consistent results from complementary ^1^H NMR, UV–vis, and fluorescence emission experiments.

**Figure 10:**
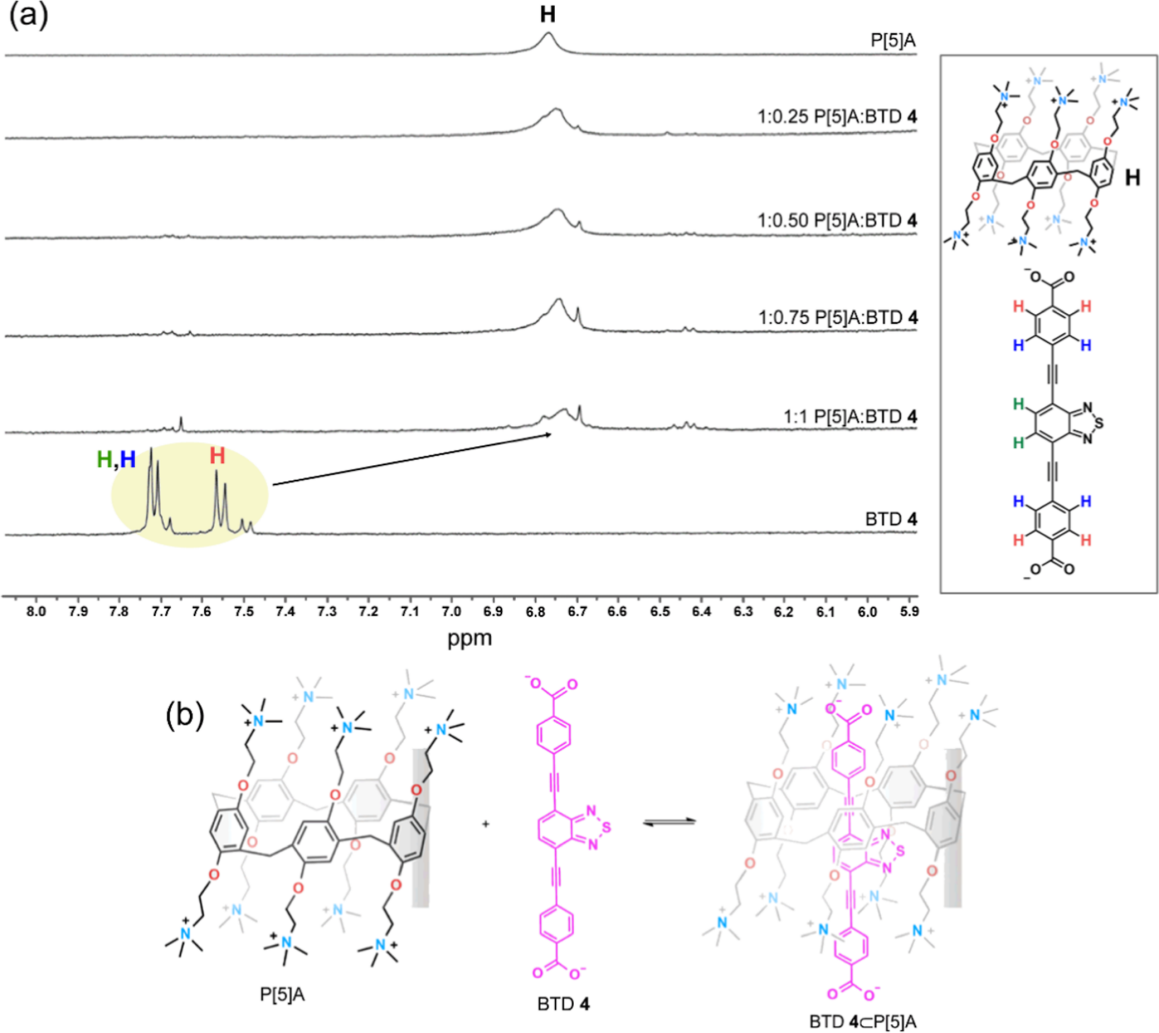
(a) ^1^H NMR spectra in D_2_O at 298 K, from top to bottom: pure cationic P[5]­A (1.0 mM), mixtures of BTD **4**:P­[5]­A at different ratios, and pure BTD **4** (1 mM). (b) Proposed inclusion complex BTD **4**⊂P­[5]­A formed by the charged host (P[5]­A) and guest (BTD **4**).

### 3.5 Molecular Docking

For gain deeper insight into the assembly of the BTD-pillar[5]­arene inclusion complex, we performed molecular docking simulations assuming a 1:1 stoichiometry. The representative interaction modes are shown in Figure 11, illustrating both the formation of inclusion and side-complexes for BTD **4**, as well as inclusion complexes for its monoanionic and neutral species (**2b** and **2**). The calculated negative free energy values (∼−8.5 kcal mol^–1^) reflects the stability of the inclusion complex (Figure 11a). Importantly, the formation of a side-complex (Figure 11b) in the case of the dianionic species (**4**) cannot be excluded. In this arrangement, the distance between the two negative charges of BTD **4** is suitable for electrostatic interactions with the ammonium groups at both portals of pillar[5]­arene, while the benzothiadiazole π-system remains accommodated within the aromatic cavity. Alternatively, a similar electrostatic interaction can occur at only one portal of the macrocycle, a configuration that is slightly lower in energy (−0.07 kcal mol^–1^), likely due to reduced steric hindrance. These results highlight electrostatic interactions as the primary driving force governing interactions the supramolecular guest–host assembly between cationic ammonium pillar[5]­arene p[5]­A and the deprotonated BTD **4**. Among the docking outcomes, the lowest free energy values were obtained for the monoprotonated species (−8.74 kcal mol^–1^), whereas the neutral form displayed slightly higher values (−8.56 kcal mol^–1^) (Figure 11c,d). This indicates that all protonation states of BTD can contribute to complexation with cationic P[5]­A, with additional stabilization inside the cavity provided by hydrophobic interactions.

**Figure 11:**
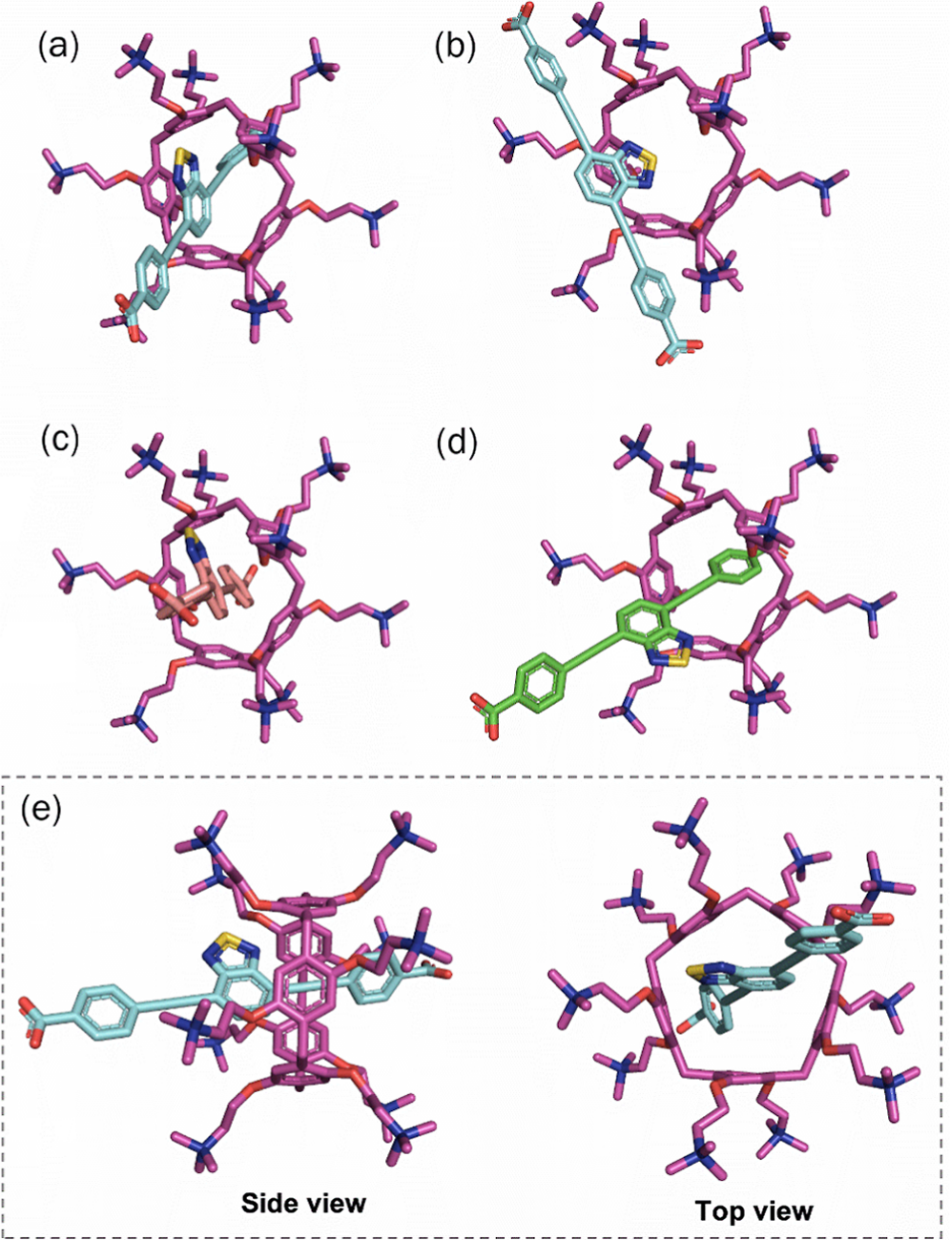
Simulated interaction modes (“poses”) obtained by molecular docking between (a,b) dianionic BTD **4**, (c) monoanionic BTD **2b**, and (d) neutral BTD **2** with P[5]­A. The calculated lowest free energy values were −8.19, −8.26, −8.74, and −8.56 kcal mol^–1^ respectively. (e) Different modes of the BTD **4**⊂P­[5]­A complex.

It is well established that complex formation is thermodynamically favorable when the free energy decreases (Δ*G* < 0) compared to the unbound components. In guest–host supramolecular systems, stabilization arises from the combined effects of noncovalent interactions, including hydrophobic and electrostatic forces.^99^ In the case of BTD and P[5]­A, we proposed that electrostatic interactions are the dominant driving forces, owing to the positive charges on the ammonium groups located on the exterior of the macrocyclic cavity. These are complemented by secondary π-interactions within the cavity, whose dimensions are suitable to accommodate a BTD molecule. Hereafter, the inclusion complex of the charged species will be denoted as BTD **4**⊂P­[5]­A. This assignment is further supported by the absence of significant changes in the fluorophore emission spectra and the lack of fluorescence quenching when the neutral guest–host pair was evaluated.

### 3.6 Photophysical Response

The sensing experiments described in this study were conducted as qualitative proof-of-concept demonstrations to highlight the sensitivity of the complex to different environments. To illustrate this responsiveness, we investigated their performance under diverse chemical conditions, including different anions and biomacromolecules. These exploratory case studies serve as examples of how such systems could be further developed and point toward potential directions for future applications. In this way, motivated by the guest–host interaction study results, we prepared 1:1 mixtures of BTD **4**⊂P­[5]­A (10^–6^ M) in aqueous solution, allowed them to equilibrate for 1 h, and then performed titrations with different sodium salts. The fluorescence signal was monitored by tracking the displacement of the fluorophore guest from the nonemissive supramolecular complex until the intensity reached that of free BTD in solution. Salt concentrations were gradually increased from 10^–4^ to 10^–2^ M. Among the salts tested, complete BTD displacement was observed only in the presence of NaBr (Figure 12a), as the fluorescence emission maximum matched that of the free fluorophore. Notably, NaBr was also the only case where the emission maximum did not exhibit a slightly blue-shifted (−20 nm) relative to free BTD, indicating that displacement occurs even at low NaBr concentrations. For NaI (Figure 12b), a pronounced increase in fluorescence was detected upon the first aliquots (∼10^–4^ M), suggesting higher sensitivity toward iodide. However, the final emission intensity did not reach that of the free fluorophore, indicating incomplete displacement. Similar behavior was observed for NaCl (Figure 12c), NaNO_3_, and Na_2_CO_3_ (Figures 12d,e). The “off–on” sensing ability was also tested with sodium azide (Figure 12f), but the fluorescence response followed the same trend, showing no selectivity since all anions produced detectable signals within the tested concentration range (10^–4^–10^–2^ M). Binding constants (*K*_b_) were calculated for all anions using Langmuir isotherms (Supporting Information):^86^ (a) NaBr, *K*_b_ = 6.34 × 10^2^ M^–1^; (b) NaI, *K*_b_ = 3.89 × 10^3^ M^–1^; (c) NaCl, *K*_b_ = 5.80 × 10^2^ M^–1^; (d) NaNO_3_, *K*_b_ = 7.83 × 10^2^ M^–1^; (e) NaCO_3_, *K*_b_ = 6.46 × 10^2^ M^–1^; and (f) NaN_3_, *K*_b_ = 9.62 × 10^2^ M^–1^.

**Figure 12:**
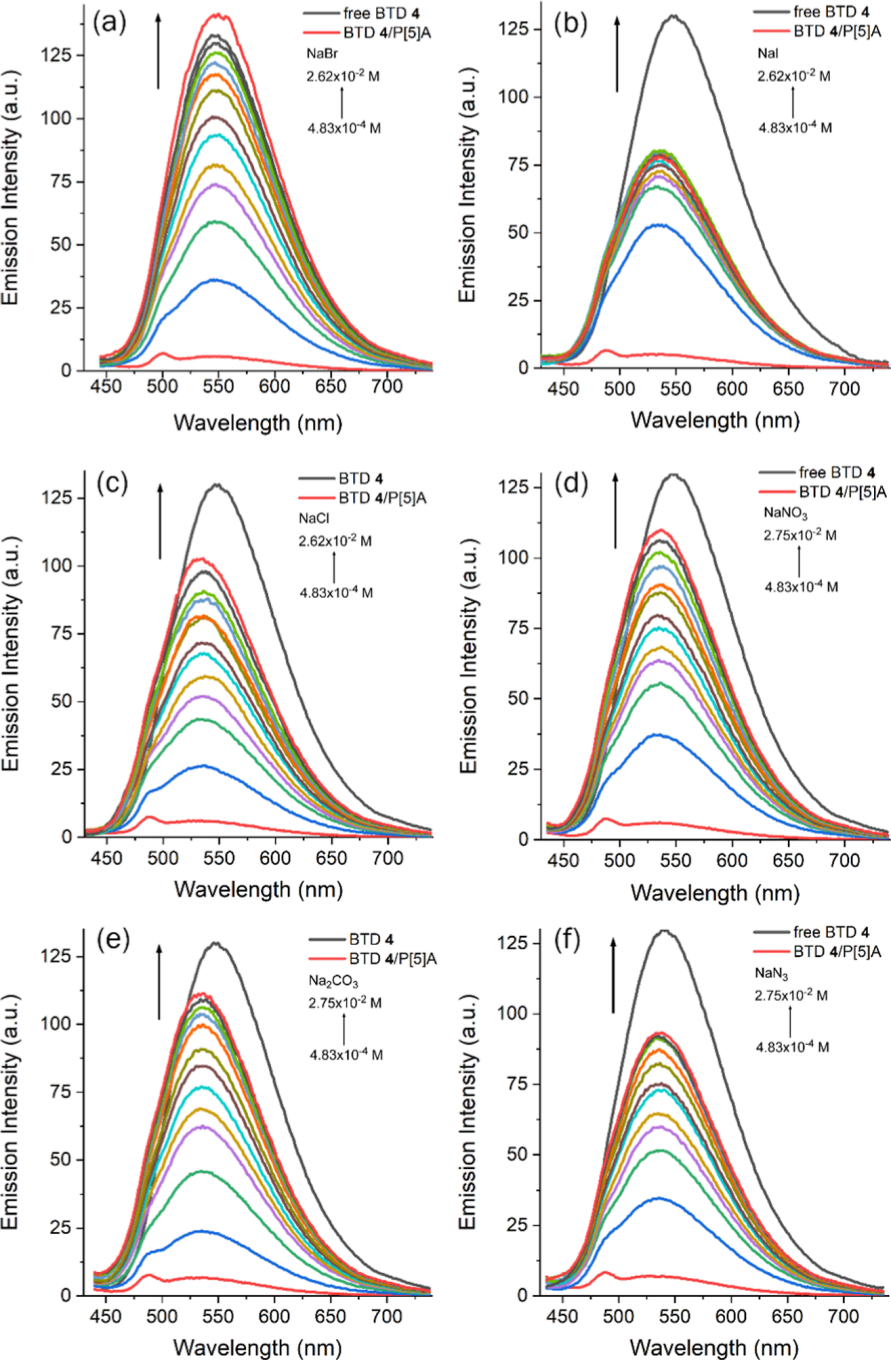
Spectrofluorimetric titration (λ_exc_ = 428 nm, excitation/emission slits: 10.0 nm/10.0 nm) of BTD **4**⊂P­[5]­A in water (10^–6^ M) with various anions (10^–4^ to 10^–2^ M): (a) NaBr, (b) NaI, (c) NaCl, (d) NaNO_3_, (e) Na_2_CO_3_ and (f) NaN_3_. The pure BTD **4** and the 1:1 BTD **4**⊂P­[5]­A complex (10^–6^ M) are presented for comparison.

It is important to note that the effects of the anions on the free guest and host molecules were also examined to rule out interference in supramolecular probing. In the absence of the BTD **4**⊂P­[5]­A complex, the fluorescence emission of BTD at 552 nm showed only a slight decrease upon addition of iodine, while no changes were observed in the very weak fluorescence of the macrocycle (Supporting Information). These findings confirm that the increase in fluorescence intensity observed in the presence of anions arises from the disassembly of the BTD **4**⊂P­[5]­A supramolecular complex. A comparative histogram showing the relative fluorescence intensities of BTD **4**⊂P­[5]­A in the presence of various anions is provided in the Supporting Information to facilitate visualization of the sensing behavior.

Since the complex exhibited poor selectivity toward simple anions, we were motivated to investigate its potential for DNA sensing, given that DNA provides a polyanionic environment. It is well established that DNA can interact noncovalently with various aromatic heterocyclic compounds through reversible van der Waals and electrostatic interactions, without perturbing the biomolecule’s structure. This property represents a significant advantage for biomolecular probing or staining. Spectrofluorimetric titrations with calf thymus DNA (ct-DNA), performed using the same protocol applied to sodium salts, demonstrated that BTD **4**⊂P­[5]­A is also effective for ct-DNA detection, even at lower concentrations (10^–7^–10^–5^ M). The nonemissive complex diluted to 10^–6^ M exhibited a pronounced increase in the fluorescence intensity of the free BTD compound upon successive ct-DNA additions, without complete displacement, and with a higher calculated binding constant (*K*_b_ = 2.33 × 10^5^ M^–1^) (Figure 13a and Supporting Information).

**Figure 13:**
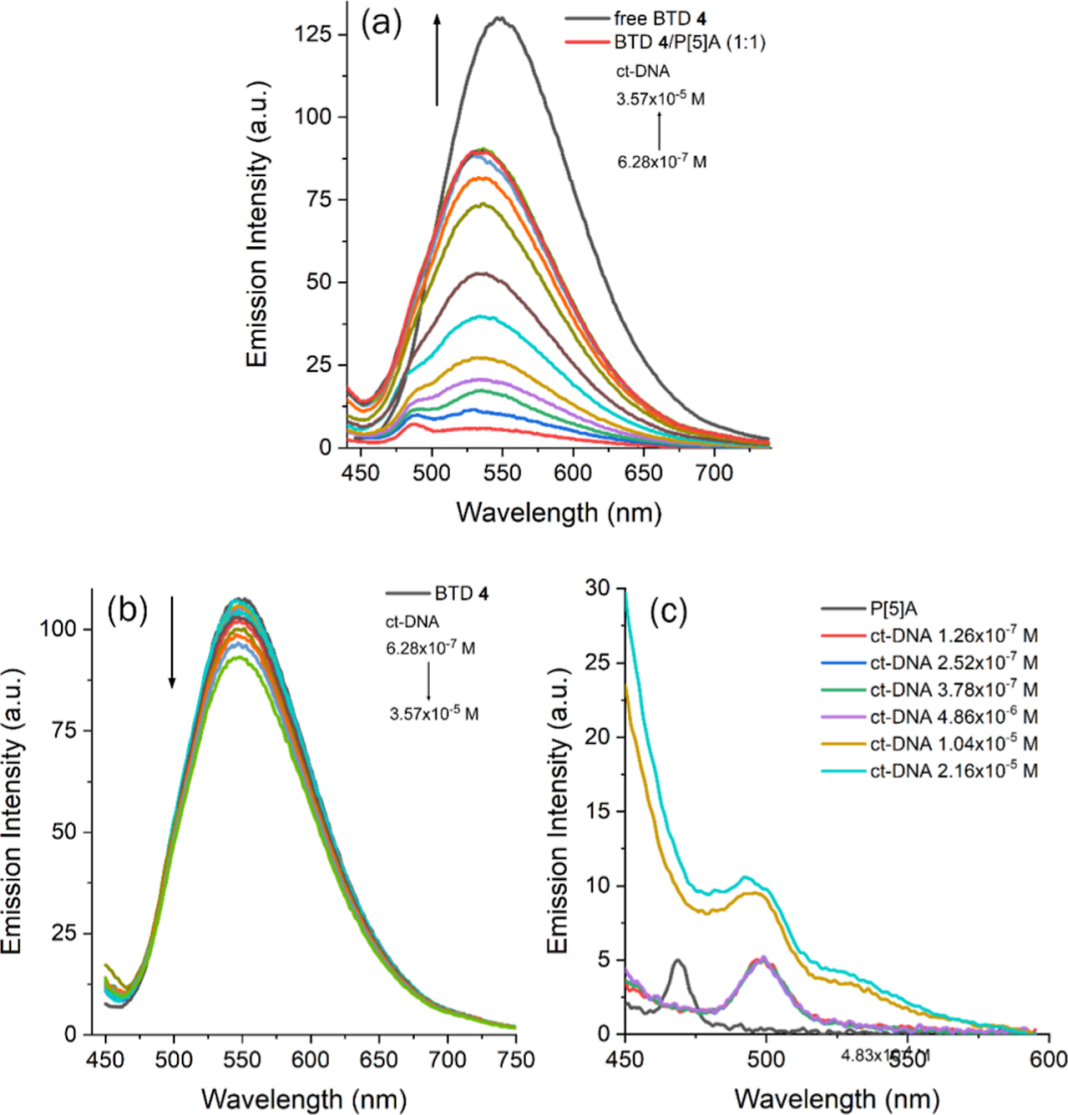
(a) Spectrofluorimetric titration of the BTD **4**⊂P­[5]­A complex in water (1.0 × 10^–6^ M) with ct-DNA (1.0 × 10^–7^–1.0 × 10^–5^ M). (b) Control experiment with pure BTD **4** in water (1.0 × 10^–6^ M). (c) Control experiment with pure P[5]­A in water (1.0 × 10^–6^ M). All spectra were recorded at λ_exc_ = 428 nm with excitation/emission slit widths of 10.0 nm/10.0 nm.

Control titrations of ct-DNA with the isolated host (P[5]­A) and guest (BTD **4**) confirmed that the observed enhancement arises from supramolecular disassembly (Figure 13b,c), supporting the application of BTD **4**⊂P­[5]­A as an “off–on” sensor for anionic biomolecules. As shown in Figure 13b, a slight decrease in BTD fluorescence intensity was observed in the presence of ct-DNA, with no shift in the emission wavelength. Planar aromatic molecules such as BTD **4** may act as groove-binding agents, and previous studies suggest that 4,7-aryl-substituted benzothiadiazoles can serve as promising cell-imaging probes with minimal genotoxicity.^100,101^ Taken together, these results indicate that the nonemissive supramolecular complex undergoes disassembly in the presence of ct-DNA. The resulting “off–on” fluorescence response arises from the emission of the released fluorophore, which can further interact with ct-DNA grooves to enable efficient biomolecular detection.

## 4 Conclusions

In this work, we synthesized benzothiadiazole derivatives and demonstrated their integration into guest–host assemblies with functionalized pillar[5]­arenes. Photophysical characterization combined with spectroscopic and theoretical analyses confirmed the formation of stable 1:1 BTD **4**⊂P­[5]­A complexes (formed by the anionic BTD **4** and cationic P[5]­A), driven primarily by electrostatic attraction and secondary π–π interactions. The complexes exhibit static fluorescence quenching, which can be reversed through competitive guest exchange with simple anions or biomacromolecules such as DNA. This reversible “off–on” switching highlights the potential of these systems for selective sensing of anionic species in dilute aqueous media (10^–7^–10^–5^ M). Beyond the experimental findings, quantum-chemical calculations provided a consistent rationale for the observed emission behavior and structure–property relationships. Overall, the integration of highly emissive anionic BTD fluorophores with cationic pillar[5]­arenes expands the toolbox of supramolecular sensors and offers a versatile strategy for designing responsive materials for environmental and biological applications.

## Supplementary Material



## Data Availability

The data underlying this study are available in the article, Supporting Information and from the corresponding author upon reasonable request.

## Notes

A detailed spectroscopic characterization, additional photophysical data and additional theoretical data are available (PDF)

## References

[1] Bode S. A., Minten I. J., Nolte R. J., Cornelissen J. J. (2011). Reactions
inside nanoscale protein cages. Nanoscale.

[2] Hof F., Craig S. L., Nuckolls C., Rebek (2002). Molecular encapsulation. Angew. Chem., Int. Ed..

[3] Ma X., Zhao Y. (2015). Biomedical applications
of supramolecular systems based on host–guest
interactions. Chem. Rev..

[4] Guo F., Sun Y., Xi B., Diao G. (2018). Recent progress in the research on
the host-guest chemistry of pillar­[*n*]­arenes. Supramol. Chem..

[5] Yu G., Jie K., Huang F. (2015). Supramolecular
amphiphiles based on host–guest
molecular recognition motifs. Chem. Rev..

[6] Yan M., Zhou J. (2023). Pillararene-based supramolecular polymers for cancer therapy. Molecules.

[7] Ogoshi T., Nishida Y., Yamagishi T. A., Nakamoto Y. (2010). High yield synthesis
of polyrotaxane constructed from pillar[5]­arene and viologen polymer
and stabilization of its radical cation. Macromolecules.

[8] Ogoshi T., Kanai S., Fujinami S., Yamagishi T. A., Nakamoto Y. (2008). para-Bridged symmetrical pillar[5]­arenes: Their Lewis
acid catalyzed synthesis and host–guest property. J. Am. Chem. Soc..

[9] Ogoshi T., Kitajima K., Yamagishi T. A., Nakamoto Y. (2010). Synthesis and conformational
characteristics of nonsymmetric pillar[5]­arene. Org. Lett..

[10] Ogoshi T., Aoki T., Kitajima K., Fujinami S., Yamagishi T. A., Nakamoto Y. (2011). Facile, rapid, and
high-yield synthesis of pillar[5]­arene
from commercially available reagents and its X-ray crystal structure. J. Org. Chem..

[11] Ogoshi T., Yamagishi T. A., Nakamoto Y. (2016). Pillar-shaped macrocyclic hosts pillar­[*n*]­arenes: New key players for supramolecular chemistry. Chem. Rev..

[12] Fang Y., Deng Y., Dehaen W. (2020). Tailoring
pillararene-based receptors
for specific metal ion binding: From recognition to supramolecular
assembly. Coord. Chem. Rev..

[13] Zhu H., Li Q., Zhu W., Huang F. (2022). Pillararenes as versatile building
blocks for fluorescent materials. Acc. Mater.
Res..

[14] Li H., Yang Y., Xu F., Liang T., Wen H., Tian W. (2019). Pillararene-based supramolecular
polymers. Chem. Commun..

[15] Wu X., Gao L., Hu X. Y., Wang L. (2016). Supramolecular drug delivery systems
based on water-soluble pillar­[*n*]­arenes. Chem. Rec..

[16] Wang K., Wang C. Y., Zhang Y., Zhang S. X. A., Yang B., Yang Y. W. (2014). Ditopic pillar[5]­arene-based
fluorescence enhancement
material mediated by [*c*2]­daisy chain formation. Chem. Commun..

[17] Yang K., Chao S., Zhang F., Pei Y., Pei Z. (2019). Recent advances
in the development of rotaxanes and pseudorotaxanes based on pillar­[*n*]­arenes: From construction to application. Chem. Commun..

[18] Feng W. X., Sun Z., Barboiu M. (2018). Pillar­[*n*]­arenes for construction of
artificial transmembrane channels. Isr. J. Chem..

[19] Liz D. G., Manfredi A. M., Medeiros M., Montecinos R., Gómez-González B., Garcia-Rio L., Nome F. (2016). Supramolecular phosphate transfer catalysis by pillar[5]­arene. Chem. Commun..

[20] Pathak R.
K., Tabbasum K., Rai A., Panda D., Rao C. P. (2012). Pyrophosphate
sensing by a fluorescent Zn^2+^ bound triazole linked imino-thiophenyl
conjugate of calix[4]­arene in HEPES buffer medium: spectroscopy, microscopy,
and cellular studies. Anal. Chem..

[21] Sathiyajith C., Shaikh R. R., Han Q., Zhang Y., Meguellati K., Yang Y. W. (2017). Biological and related applications of pillar­[*n*]­arenes. Chem. Commun..

[22] Mummidivarapu V. V. S., Joseph R., Rao C. P., Pathak R. K. (2023). Suprareceptors emerging
from click chemistry: Comparing the triazole based scaffolds of calixarenes,
cyclodextrins, cucurbiturils and pillararenes. Coord. Chem. Rev..

[23] Fang Y., Li C., Wu L., Bai B., Li X., Jia Y., Feng W., Yuan L. (2015). A non-symmetric
pillar[5]­arene based
on triazole-linked 8-oxyquinolines as a sequential sensor for thorium
(IV) followed by fluoride ions. Dalton Trans..

[24] Joseph R. (2020). Selective
detection of Fe^3+^, F^–^, and cysteine by
a novel triazole-linked decaamine derivative of pillar[5]­arene and
its metal ion complex in water. ACS Omega.

[25] Stepanova V. B., Shurpik D. N., Evtugyn V. G., Stoikov I. I., Evtugyn G. A., Osin Y. N., Hianik T. (2016). Label-free electrochemical
aptasensor
for cytochrome c detection using pillar[5]­arene bearing neutral red. Sens. Actuators B Chem..

[26] Yang S., Liu L., You M., Zhang F., Liao X., He P. (2016). The novel
pillar[5]­arene derivative for recyclable electrochemical sensing platform
of homogeneous DNA hybridization. Sens. Actuators
B Chem..

[27] Yu G., Wu D., Li Y., Zhang Z., Shao L., Zhou J., Hu Q., Tang G., Huang F. (2016). A pillar[5]­arene-based (2) rotaxane
lights up mitochondria. Chem. Sci..

[28] Zhou J., Chen M., Diao G. (2014). Synthesis of the first amphiphilic
pillar[6]­arene and its enzyme-responsive self-assembly in water. Chem. Commun..

[29] Cao D., Meier H. (2019). Pillararene-based fluorescent
sensors for the tracking of organic
compounds. Chin. Chem. Lett..

[30] Cui W., Wang L., Xu L., Zhang G., Meier H., Tang H., Cao D. (2018). Fluorescent-cavity host: An efficient
probe to study supramolecular recognition mechanisms. J. Phys. Chem. Lett..

[31] Lin Q., Zhong K. P., Zhu J. H., Ding L., Su J. X., Yao H., Wei T. B., Zhang Y. M. (2017). Iodine controlled pillar[5]­arene-based
multiresponsive supramolecular polymer for fluorescence detection
of cyanide, mercury, and cysteine. Macromolecules.

[32] Tan L. L., Yang Y. W. (2015). Molecular recognition
and self-assembly of pillarenes. J. Incl. Phenom.
Macro..

[33] Hua B., Shao L., Yu G., Huang F. (2016). Fluorescence indicator
displacement detection based on pillar[5]­arene-assisted dye deprotonation. Chem. Commun..

[34] Lin Q., Liu L., Zheng F., Mao P. P., Liu J., Zhang Y. M., Yao H., Wei T. B. (2017). A novel water soluble self-assembled supramolecular
sensor based on pillar[5]­arene for fluorescent detection CN^–^ in water. Tetrahedron.

[35] Lin Q., Zheng F., Liu L., Mao P. P., Zhang Y. M., Yao H., Wei T. B. (2016). Efficient sensing of fluoride ions in water using a
novel water soluble self-assembled supramolecular sensor based on
pillar[5]­arene. RSC Adv..

[36] Lin Q., Mao P. P., Zheng F., Liu L., Liu J., Zhang Y. M., Yao H., Wei T. B. (2017). Novel supramolecular
sensors constructed from pillar[5]­arene and a naphthalimide for efficient
detection of Fe^3+^ and F^–^ in water. New J. Chem..

[37] Wanderlind E. H., Liz D. G., Gerola A. P., Affeldt R. F., Nascimento V., Bretanha L. C., Montecinos R., García-Río L., Fiedler H. D., Nome F. (2018). Imidazole-functionalized
pillar­[5]­arenes:
Highly reactive and selective supramolecular artificial enzymes. ACS Catal..

[38] Vieira
Silveira E., Montecinos R., Scorsin L., Garcia-Rio L., Medeiros M., Nascimento V., Nome F., Affeldt R. F., Micke G. A. (2021). Supramolecular kinetic effects by pillararenes: the
synergism between spatiotemporal and preorganization concepts in decarboxylation
reactions. New J. Chem..

[39] Silveira E. V., Nascimento V., Wanderlind E. H., Affeldt R. F., Micke G. A., García-Río L., Nome F. (2019). Inhibitory and cooperative
effects regulated by pH in host–guest complexation between
cationic pillar[5]­arene and reactive 2-carboxyphthalanilic acid. J. Org. Chem..

[40] Silveira E. V., Wanderlind E. H., Masson A. K., Cordeiro P. S., Nascimento V., Affeldt R. F., Micke G. A. (2020). Molecular recognition of methamphetamine
by carboxylatopillar[5]­arene: Drug-dependent complexation stoichiometry
and insights into medical applications. New
J. Chem..

[41] Demos W., Micke L. C. B., Lacerda L. H. S., Souza B. S., Gerola A. P., Affeldt R. F. (2024). Supramolecular assembly
between cationic pyridinium-pillararene
and aminosalicylate drug. J. Braz. Chem. Soc..

[42] Xiong W., Tang S., Murto P., Zhu W., Edman L., Wang E. (2019). Combining benzotriazole and benzodithiophene
host units in host–guest
polymers for efficient and stable near-infrared emission from light-emitting
electrochemical cells. Adv. Opt. Mater..

[43] Li S., Liu Q., Mao L., Zhang X., Li C., Ma D. (2024). Benzothiadiazole-based
water-soluble macrocycle: Synthesis, aggregation-induced emission
and selective detection of spermine. Chin. Chem.
Lett..

[44] Chakraborty D., Kaur N., Sahoo J., Hickey N., De M., Mukherjee P. S. (2024). Host–guest
interactions induced enhancement
in oxidase-like activity of a benzothiadiazole dye inside an aqueous
Pd_8_L_4_ barrel. J. Am. Chem.
Soc..

[45] Hua B., Zhang C., Zhou W., Shao Li., Wang Z., Wang L., Zhu H., Huang F. (2020). Pillar­[5]­arene-Based
Solid-State Supramolecular Polymers with Suppressed Aggregation-Caused
Quenching Effects and Two-Photon Excited Emission. J. Am. Chem. Soc..

[46] Maisonneuve S., Fang Q., Xie J. (2008). Benzothiadiazoyl-triazoyl cyclodextrin:
A selective fluoroionophore for Ni­(II). Tetrahedron.

[47] Moro A. V., Ferreira P. C., Migowski P., Rodembusch F. S., Dupont J., Lüdtke D. S. (2013). Synthesis
and photophysical properties
of fluorescent 2,1,3-benzothiadiazole-triazole-linked glycoconjugates:
selective chemosensors for Ni­(II). Tetrahedron.

[48] Wang B., Pan H., Jia J., Ge Y. Q., Cai W. Q., Wang J. W., Zhao C. H. (2014). Highly
emissive dimesitylboryl-substituted 2,1,3-benzothiadiazole
derivatives: Photophysical properties and efficient fluorescent sensor
for fluoride anions. Tetrahedron.

[49] Çimen O., Dinçalp H., Varlikli C. (2015). Studies on UV–vis and fluorescence
changements in Co^2+^ and Cu^2+^ recognition by
a new benzimidazole–benzothiadiazole derivative. Sens. Actuators B Chem..

[50] Zhang Q., Zhang J., Zuo H., Wang C., Shen Y. (2016). A novel colorimetric
and fluorescent sensor for cyanide anions detection based on triphenylamine
and benzothiadiazole. Tetrahedron.

[51] Hu Z., Feng L., Yang P. (2024). 2,1,3-Benzothiadiazole
derivative
small molecule fluorophores for NIR-II bioimaging. Adv. Funct. Mater..

[52] Wang Z., Peng Z., Huang K., Lu P., Wang Y. (2019). Butterfly-shaped
π-extended benzothiadiazoles as promising emitting materials
for white OLEDs. J. Mater. Chem. C.

[53] Sudyoadsuk T., Chasing P., Chaiwai C., Chawanpunyawat T., Kaewpuang T., Manyum T., Namuangruk S., Promarak V. (2020). Highly fluorescent solid-state benzothiadiazole derivatives
as saturated red emitters for efficient solution-processed non-doped
electroluminescent devices. J. Mater. Chem.
C.

[54] Fang Q., Xu B., Jiang B., Fu H., Chen X., Cao A. (2005). Bisindoles
containing a 2,1,3-benzothiadiazole unit: novel non-doping red organic
light-emitting diodes with excellent color purity. Chem. Commun..

[55] Salami F., Farshi H., Zhao Y., Chen B. (2025). Benzothiadiazole-centered
donor–acceptor–donor systems: Synthesis, characterization,
and PFAS-induced fluorochromism. J. Org. Chem..

[56] Liu R., Zhou C., Ding Q., Qu L., Wang K., Tang H., Li Y., Yang C. (2023). Dual-state emission
of D-A-D type benzothiadiazole derivatives for the sensitive detection
of amine compounds. Dyes Pigm..

[57] Du J., Biewer M. C., Stefan M. C. (2016). Benzothiadiazole building units in
solution processable small molecules for organic photovoltaics. J. Mater. Chem. A.

[58] Zhang X., Bronstein H., Kronemeijer A. J., Smith J., Kim Y., Kline R. J., Richter L. J., Anthopoulos T. D., Sirringhaus H., Song K., Heeney M., Zhang W., McCulloch I., DeLongchamp D. M. (2013). Molecular origin of high field-effect
mobility in an indacenodithiophene–benzothiadiazole copolymer. Nat. Commun..

[59] Zhang D., Yang T., Xu H., Miao Y., Chen R., Shinar R., Shinar J., Wang H., Xu B., Yu J. (2021). Triphenylamine/benzothiadiazole-based
compounds for non-doped orange
and red fluorescent OLEDs with high efficiencies and low efficiency
roll-off. J. Mater. Chem. C.

[60] Isoppo V. G., Gil E. S., Gonçalves P. F.
B., Rodembusch F. S., Moro A. V. (2020). Highly fluorescent lipophilic 2,1,3-benzothiadiazole
fluorophores as optical sensors for tagging material and gasoline
adulteration with ethanol. Sens. Actuators B
Chem..

[61] Marshall R. J., Kalinovskyy Y., Griffin S. L., Wilson C., Blight B. A., Forgan R. S. (2017). Functional
versatility of a series of Zr metal–organic
frameworks probed by solid-state photoluminescence spectroscopy. J. Am. Chem. Soc..

[62] Pilgram K., Zupan M., Skiles R. (1970). Bromination of 2,1,3-benzothiadiazoles. J. Heterocycl. Chem..

[63] Neto B. A., Lapis A. A. M., da
Silva Júnior E. N., Dupont J. (2013). 2,1,3-Benzothiadiazole
and derivatives: Synthesis, properties, reactions, and applications
in light technology of small molecules. Eur.
J. Org Chem..

[64] Gutiérrez-Arzaluz L., Nadinov I., Healing G., Czaban-Jozwiak J., Jia J., Huang Z., Zhao Y., Shekhah O., Schanze K. S., Eddaoudi M., Mohammed O. F. (2021). Ultrafast aggregation-induced tunable
emission enhancement in a benzothiadiazole-based fluorescent metal–organic
framework linker. J. Phys. Chem. B.

[65] Swirepik O., Smith J. N., White N. G. (2023). Balancing on a knife’s
edge:
Studies on the synthesis of pillar[6]­arene derivatives. J. Org. Chem..

[66] Santos E. C., dos Santos T. C., Fernandes T. S., Jorge F. L., Nascimento V., Madriaga V. G., Cordeiro P. S., Checca N. R., Da Costa N. M., Pinto L. F. R. (2020). A reversible, switchable pH-driven quaternary
ammonium pillar[5]­arene nanogate for mesoporous silica nanoparticles. J. Mater. Chem..

[67] Wurth C., Grabolle M., Pauli J., Spieles M., Resch-Genger U. (2013). Relative and
absolute determination of fluorescence quantum yields of transparent
samples. Nat. Protoc..

[68] Neese F. (2025). Software Update:
The ORCA Program System, Version 6.0. WIRES
Comput. Mol. Sci..

[69] de
Souza B. (2025). GOAT: A global optimization algorithm for molecules and atomic clusters. Angew. Chem., Int. Ed..

[70] Bannwarth C., Ehlert S., Grimme S. (2019). GFN2-xTB-An Accurate and broadly
parametrized self-consistent tight-binding quantum chemical method
with multipole electrostatics and density-dependent dispersion contributions. J. Chem. Theory Comput..

[71] Chai J. D., Head-Gordon M. (2008). Long-range corrected hybrid density functionals with
damped atom-atom dispersion corrections. Phys.
Chem. Chem. Phys..

[72] Caldeweyher E., Ehlert S., Hansen A., Neugebauer H., Spicher S., Bannwarth C., Grimme S. (2019). A Generally applicable
atomic-charge dependent London dispersion correction. J. Chem. Phys..

[73] Weigend F., Ahlrichs R. (2005). Balanced basis sets
of split valence, triple zeta valence
and quadruple zeta valence quality for H to Rn: Design and assessment
of accuracy. Phys. Chem. Chem. Phys..

[74] Barone V., Cossi M. (1998). Quantum calculation of molecular energies and energy gradients in
solution by a conductor solvent model. J. Phys.
Chem. A.

[75] Runge E., Gross E. K. U. (1984). Density-functional
theory for time-dependent systems. Phys. Rev.
Lett..

[76] Casanova-Páez M., Goerigk L. (2021). Time-dependent
long-range-corrected double-hybrid density
functionals with spin-component and spin-opposite scaling: A comprehensive
analysis of singlet–singlet and singlet–triplet excitation
energies. J. Chem. Theory Comput..

[77] Hirata S., Head-Gordon M. (1999). Time-dependent density functional
theory within the
Tamm–Dancoff approximation. Chem. Phys.
Lett..

[78] Martin R. L. (2003). Natural
transition orbitals. J. Chem. Phys..

[79] Glendening E. D., Landis C. R., Weinhold F. (2019). NBO 7.0: New vistas in localized
and delocalized chemical bonding theory. J.
Comput. Chem..

[80] Frisch, M. J. ; Trucks, G. W. ; Schlegel, H. B. ; Scuseria, G. E. ; Robb, M. A. ; Cheeseman, J. R. ; Scalmani, G. ; Barone, V. ; Petersson, G. A. ; Nakatsuji, H. ; Li, X. ; Caricato, M. ; Marenich, A. V. ; Bloino, J. ; Janesko, B. G. ; Gomperts, R. ; Mennucci, B. ; Hratchian, H. P. ; Ortiz, J. V. ; Izmaylov, A. F. ; Sonnenberg, J. L. ; Williams-Young, D. ; Ding, F. ; Lipparini, F. ; Egidi, F. ; Goings, J. ; Peng, B. ; Petrone, A. ; Henderson, T. ; Ranasinghe, D. ; Zakrzewski, V. G. ; Gao, J. ; Rega, N. ; Zheng, G. ; Liang, W. ; Hada, M. ; Ehara, M. ; Toyota, K. ; Fukuda, R. ; Hasegawa, J. ; Ishida, M. ; Nakajima, T. ; Honda, Y. ; Kitao, O. ; Nakai, H. ; Vreven, T. ; Throssell, K. ; Montgomery, J. A. ; Peralta, J. E. ; Ogliaro, F. ; Bearpark, M. J. ; Heyd, J. J. ; Brothers, E. N. ; Kudin, K. N. ; Staroverov, V. N. ; Keith, T. A. ; Kobayashi, R. ; Normand, J. ; Raghavachari, K. ; Rendell, A. P. ; Burant, J. C. ; Iyengar, S. S. ; Tomasi, J. ; Cossi, M. ; Millam, J. M. ; Klene, M. ; Adamo, C. ; Cammi, R. ; Ochterski, J. W. ; Martin, R. L. ; Morokuma, K. ; Farkas, O. ; Foresman, J. B. ; Fox, D. J. Gaussian 16, Gaussian 09; Gaussian, Inc.: Wallingford CT, 2016.

[81] Morris G.
M., Huey R., Lindstrom W., Sanner M. F., Belew R. K., Goodsell D. S., Olson A. J. (2009). AutoDock4 and AutoDockTools4: Automated
docking with selective receptor flexibility. J. Comput. Chem..

[82] Strickler S. J., Berg R. A. (1962). Relationship between
absorption intensity and fluorescence
lifetime of molecules. J. Chem. Phys..

[83] Turro, N. J. ; Scaiano, J. C. ; Ramamurthy, V. Principles of Molecular Photochemistry: An Introduction; 1st edn.: University Science Books, 2008.

[84] Lawrence J., Bernal J., Witzgall C. (2019). A Purely algebraic
justification
of the Kabsch-Umeyama algorithm. J. Res. Natl.
Inst. Stand. Technol..

[85] Ogoshi T., Hashizume M., Yamagishi T. A., Nakamoto Y. (2010). Synthesis, conformational
and host–guest properties of water-soluble pillar[5]­arene. Chem. Commun..

[86] Gerola A.
P., Wanderlind E. H., Idrees M., Sangaletti P., Zaramello L., Nome R. A., Silva G. T. M., Quina F. H., Tachiya M., Nome F., Fiedler H. D. (2020). Anion binding to
surfactant aggregates: AuCl_4_
^–^ in cationic,
anionic and zwitterionic micelles. J. Mol. Liq..

[87] Guo D. S., Wang K., Liu Y. (2008). Selective binding behaviors
of p-sulfonatocalixarenes
in aqueous solution. J. Incl. Phenom. Macro..

[88] Gómez B., Francisco V., Fernández-Nieto F., Garcia-Rio L., Martín-Pastor M., Paleo M. R., Sardina F. J. (2014). Host–guest
chemistry of a water-soluble pillar[5]­arene: Evidence for an ionic-exchange
recognition process and different complexation modes. Chem.—Eur. J..

[89] Hua B., Zhou J., Yu G. (2015). Hydrophobic interactions in the pillar[5]­arene-based
host–guest complexation and their application in the inhibition
of acetylcholine hydrolysis. Tetrahedron Lett..

[90] Ghosh T., Slanina T., König B. (2015). Visible light
photocatalytic reduction
of aldehydes by Rh (III)–H: A detailed mechanistic study. Chem. Sci..

[91] Tiong P., Lintang H. O., Endud S., Yuliati L. (2015). Improved interfacial
charge transfer and visible light activity of reduced graphene oxide–graphitic
carbon nitride photocatalysts. RSC Adv..

[92] Hopkinson M. N., Gómez-Suárez A., Teders M., Sahoo B., Glorius F. (2016). Accelerated discovery in photocatalysis using a mechanism-based
screening method. Angew. Chem., Int. Ed..

[93] Gómez-González B., Francisco V., Montecinos R., García-Río L. (2017). Investigation
of the binding modes of a positively charged pillar[5]­arene: Internal
and external guest complexation. Org. Biomol.
Chem..

[94] Ma Y., Ji X., Xiang F., Chi X., Han C., He J., Abliz Z., Chen W., Huang F. (2011). A cationic water-soluble
pillar[5]­arene: Synthesis and host–guest complexation with
sodium 1-octanesulfonate. Chem. Commun..

[95] Arena G., Gentile S., Gulino F. G., Sciotto D., Sgarlata C. (2004). Water-soluble
pentasulfonatocalix­[5]­arene: Selective recognition of ditopic trimethylammonium
cations by a triple non-covalent interaction. Tetrahedron Lett..

[96] Hapiot F., Tilloy S., Monflier E. (2006). Cyclodextrins
as supramolecular hosts
for organometallic complexes. Chem. Rev..

[97] Ma Y., Xue M., Zhang Z., Chi X., Huang F. (2013). Neutral guest capture
by a cationic water-soluble pillar[5]­arene in water. Tetrahedron.

[98] Hibbert D. B., Thordarson P. (2016). The death
of the Job plot, transparency, open science
and online tools, uncertainty estimation methods and other developments
in supramolecular chemistry data analysis. Chem.
Commun..

[99] Pan Z., Zhao X., Li Q., Zhang Z., Liu Y. (2023). Recent Advances
in supramolecular-macrocycle-based nanomaterials in cancer treatment. Molecules.

[100] Neto B. A. D., Corrêa J. R., Carvalho P. H. P. R., Santos D. C. B. D., Guido B. C., Gatto C. C., Oliveira H. C. B., Fasciotti M., Eberlin M. N., da Silva
Junior E. S. (2012). Selective
and efficient mitochondrial staining with designed 2,1,3-benzothiadiazole
derivatives as live cell fluorescence imaging probes. J. Braz. Chem. Soc..

[101] Damiani A. P., Rohr P., Westrup J. L., Duarte R. C., Longaretti L. M., Rocha B. M., Strapazzon G., Venturini L. C., Souza E., Pich C. T., Frizon T. E. A., Andrade V. M., Rodembusch F. S., Dal-Bó A. G. (2018). Synthesis,
DNA interaction and genotoxic evaluation of a photoactive benzothiadiazole
with potential application in photovoltaic paint. J. Braz. Chem. Soc..

