# Supplementary material for: Responsive Supramolecular Sensors Based on Pillar[5]arene–BTD Complexes for Aqueous Sensing: From Static Quenching to Anion and DNA Recognition
Source: ACS Omega. 2025 Dec 31;11(1):1849–66. doi: 10.1021/acsomega.5c10011 (PMC12809561; doi:10.1021/acsomega.5c10011)
Supplement: Supplementary file 1 [file ao5c10011_si_001.pdf]

Supporting Information

Responsive Supramolecular Sensors Based on Pillar[5]arene-BTD Complexes for Aqueous Sensing: From Static Quenching to Anion and DNA Recognition

Débora Kélen Silva da Conceição,[1,2] Yasmin Petter da Veiga,[2] Claudiana Dotti,[2] Luis García-Río,[3] Adriana Passarela Gerola,[2] Henrique de Castro Silva Junior,[4] Fabiano Severo Rodembusch,[1] Ricardo Ferreira Affeldt,*[2] Angélica Venturini Moro*[1]

[1]Laboratory of Molecular Catalysis, Universidade Federal do Rio Grande do Sul, Porto Alegre/RS - Brazil.

[2]Laboratory of Catalysis and Interfacial Phenomena, Universidade Federal de Santa Catarina, Florianópolis/SC - Brazil.

[3]Centro Singular de Investigación en Química Biológica y Materiales Moleculares, Universidade de Santiago de Compostela, Santiago de Compostela - Spain.

[4]Departamento de Química Fundamental, Instituto de Química, Universidade Federal Rural do Rio de Janeiro, Rodovia BR-465, Km 7, Seropédica, RJ 23897-000, Brazil.

*Corresponding authors: angelica.venturini@ufrgs.br and ricardo.affeldt@ufsc.br

Summary

Spectroscopic characterization ……………………..…………………..………… 2
Additional photophysical data ……………………..……………………….…….. 15
Additional theoretical data ………………………………………………… 29

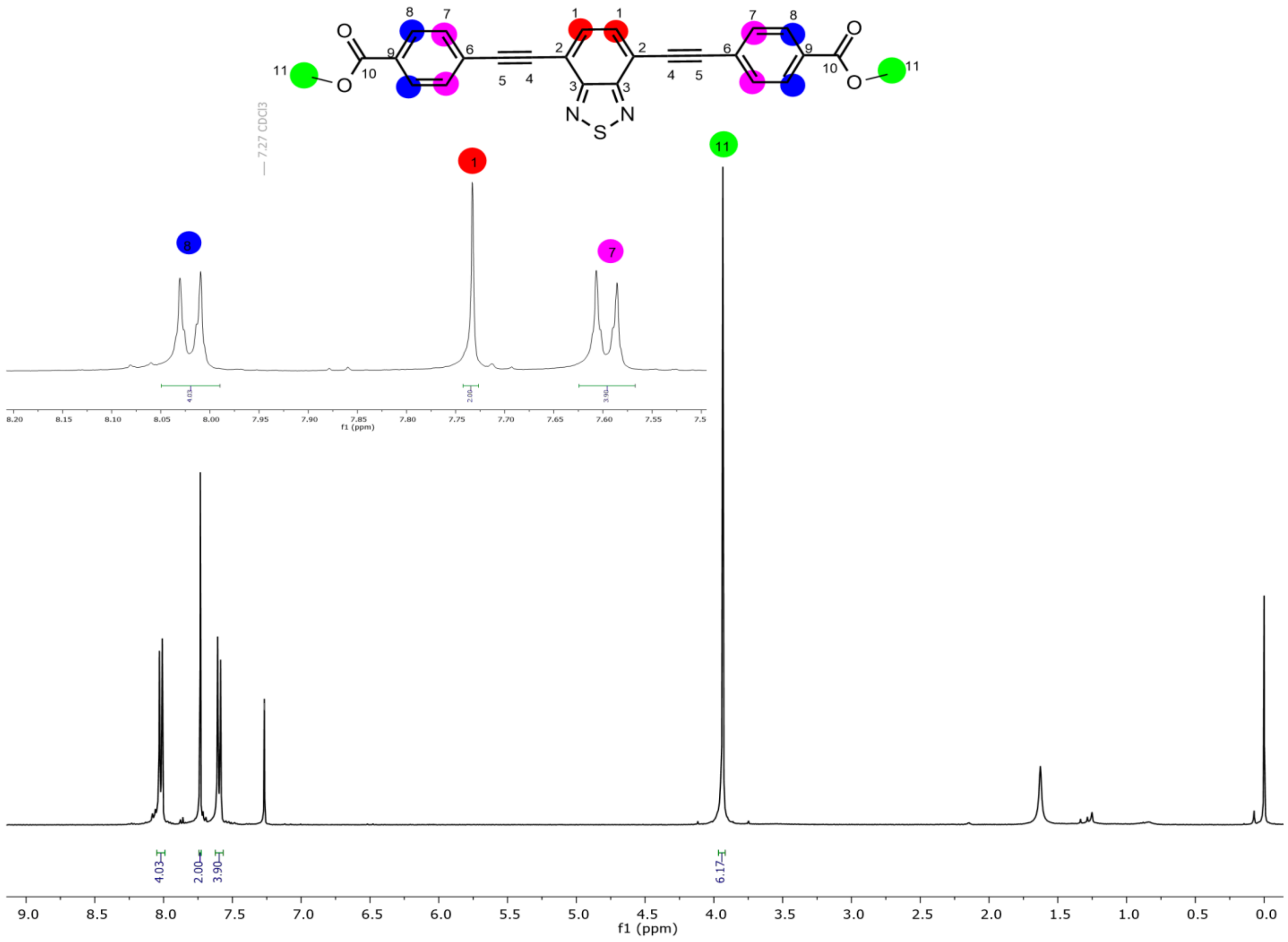

Figure S1. ^{1}H NMR spectrum of BTD **1** in $CDCl_3$ (400 MHz).

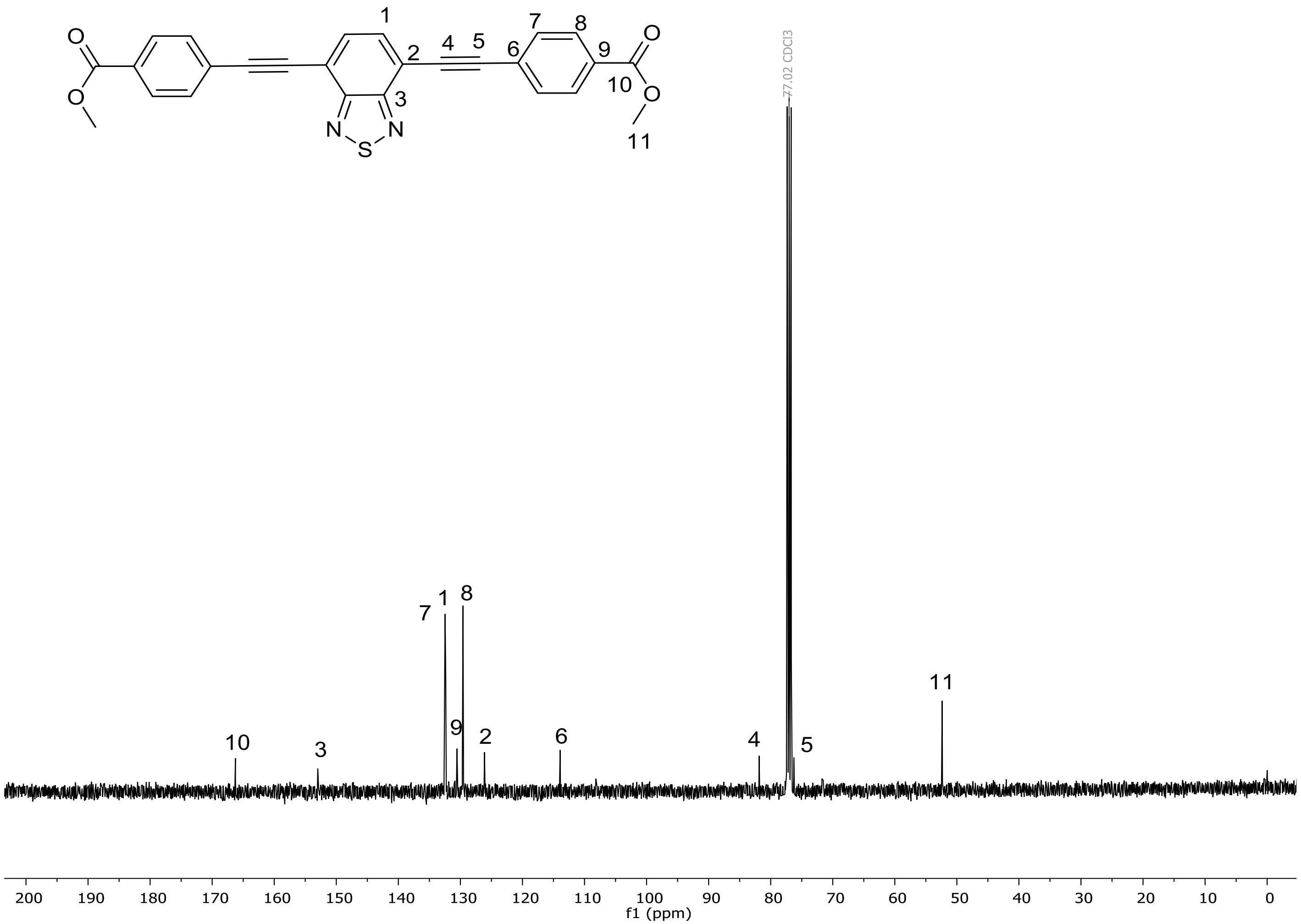

Figure S2. ^{13}C NMR spectrum of BTD **1** in $CDCl_3$ (100 MHz).

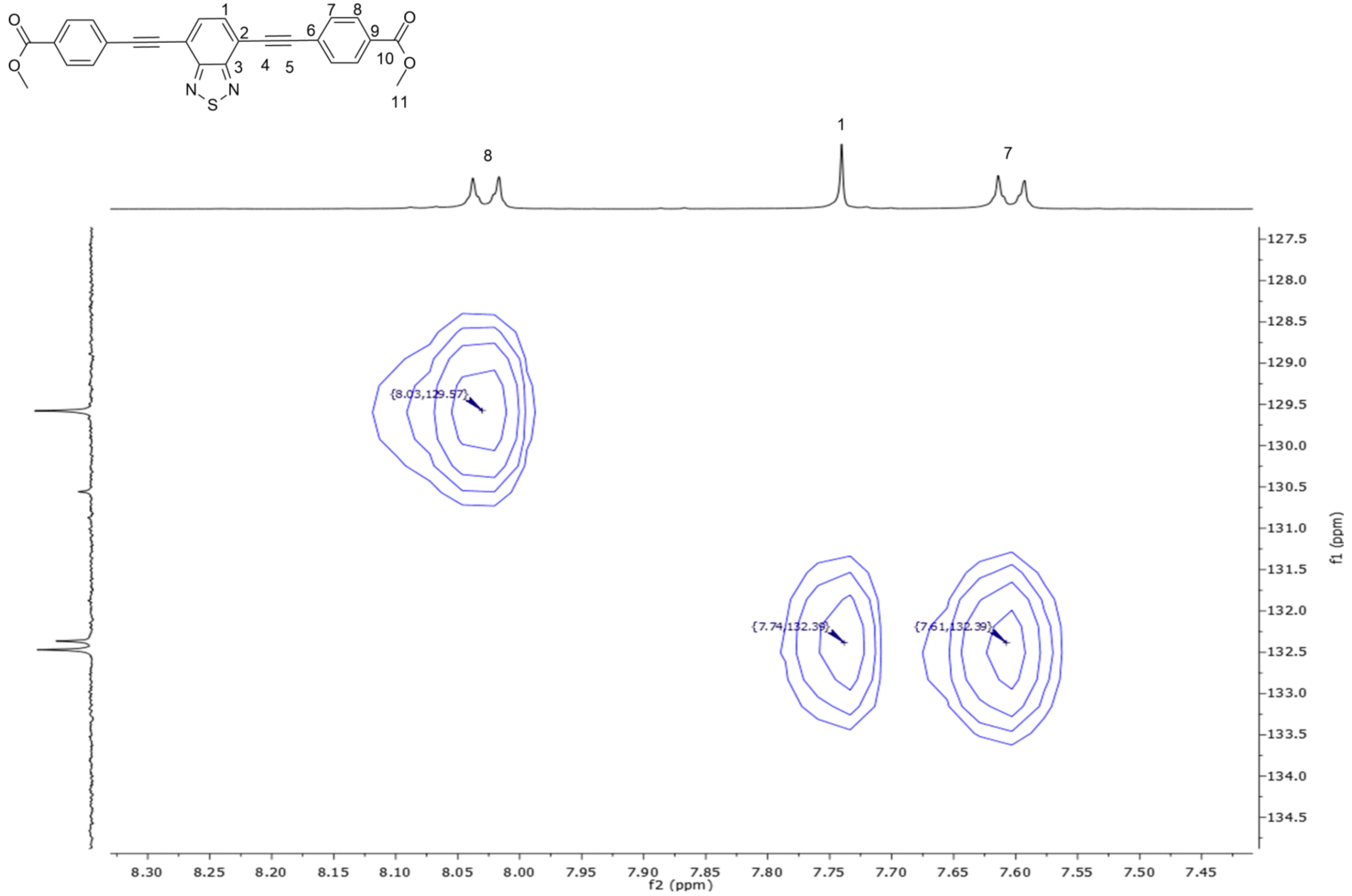

Figure S3. ^{1}H-^{13}C HSQC spectra of BTD **1** in $CDCl_3$ (400 MHz).

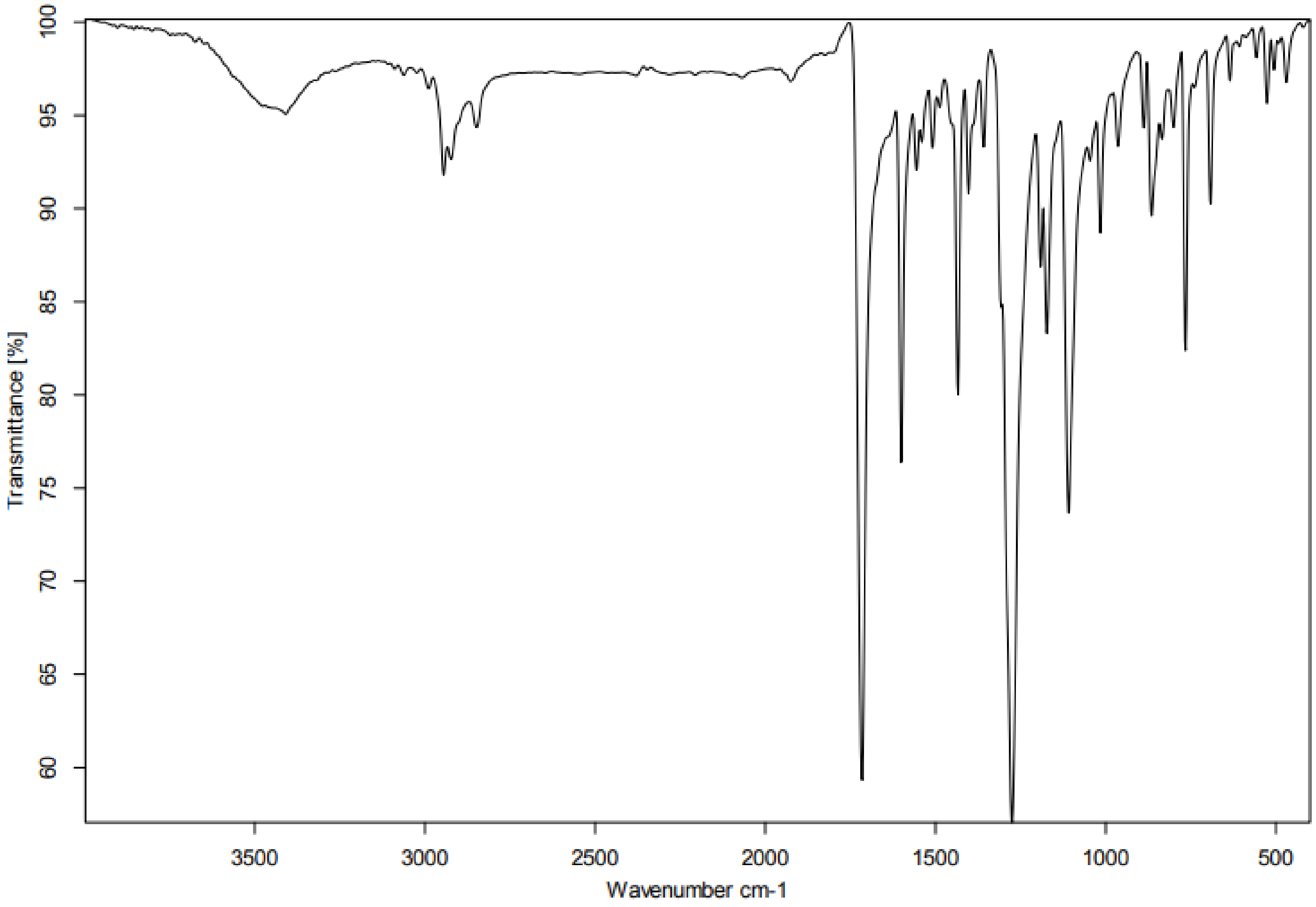

Figure S4. IR spectrum (ATR mode) of BTD **1**.

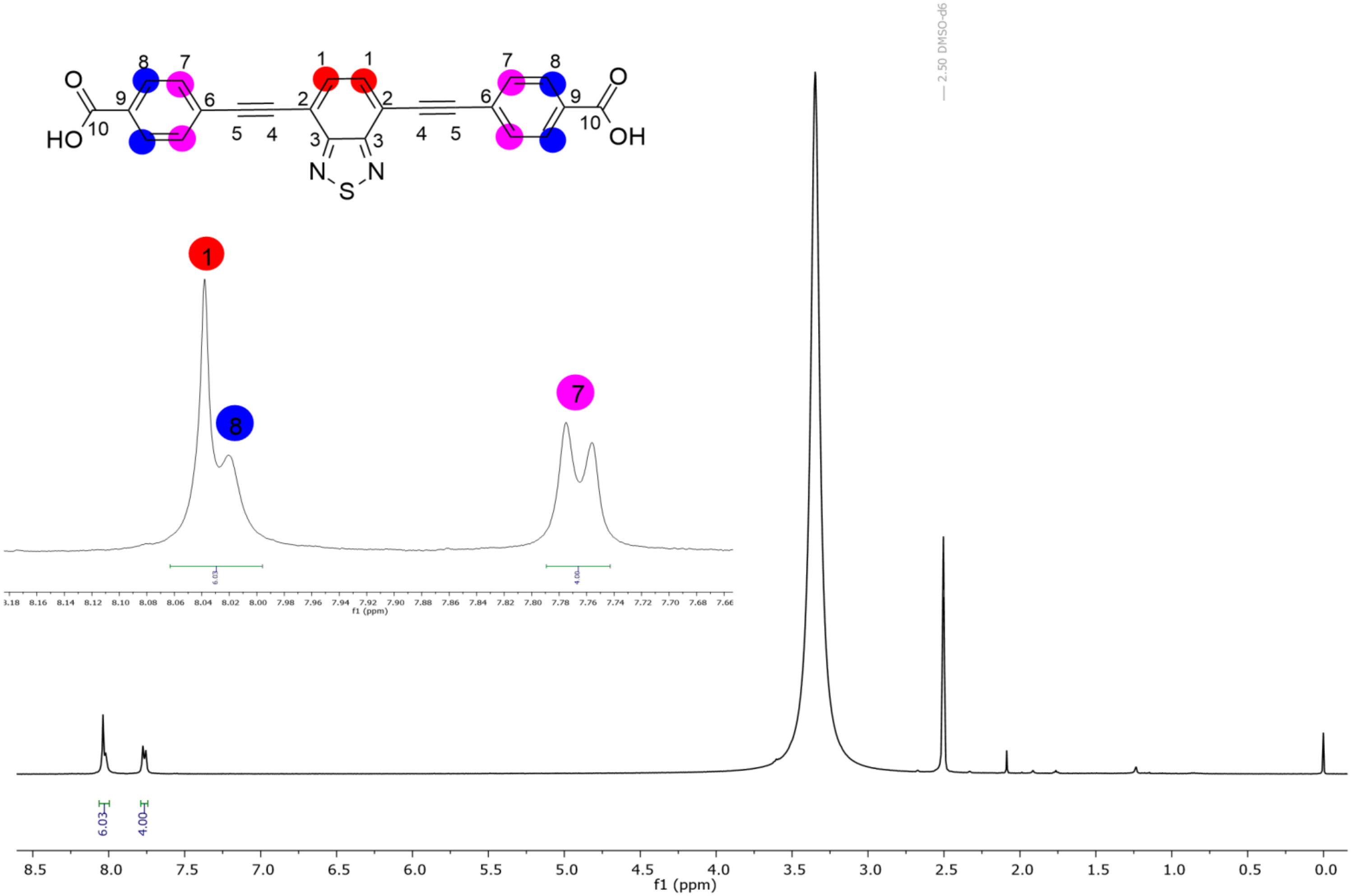

Figure S5. ^{1}H NMR spectrum of BTD **2** in DMSO-d_6 (400 MHz).

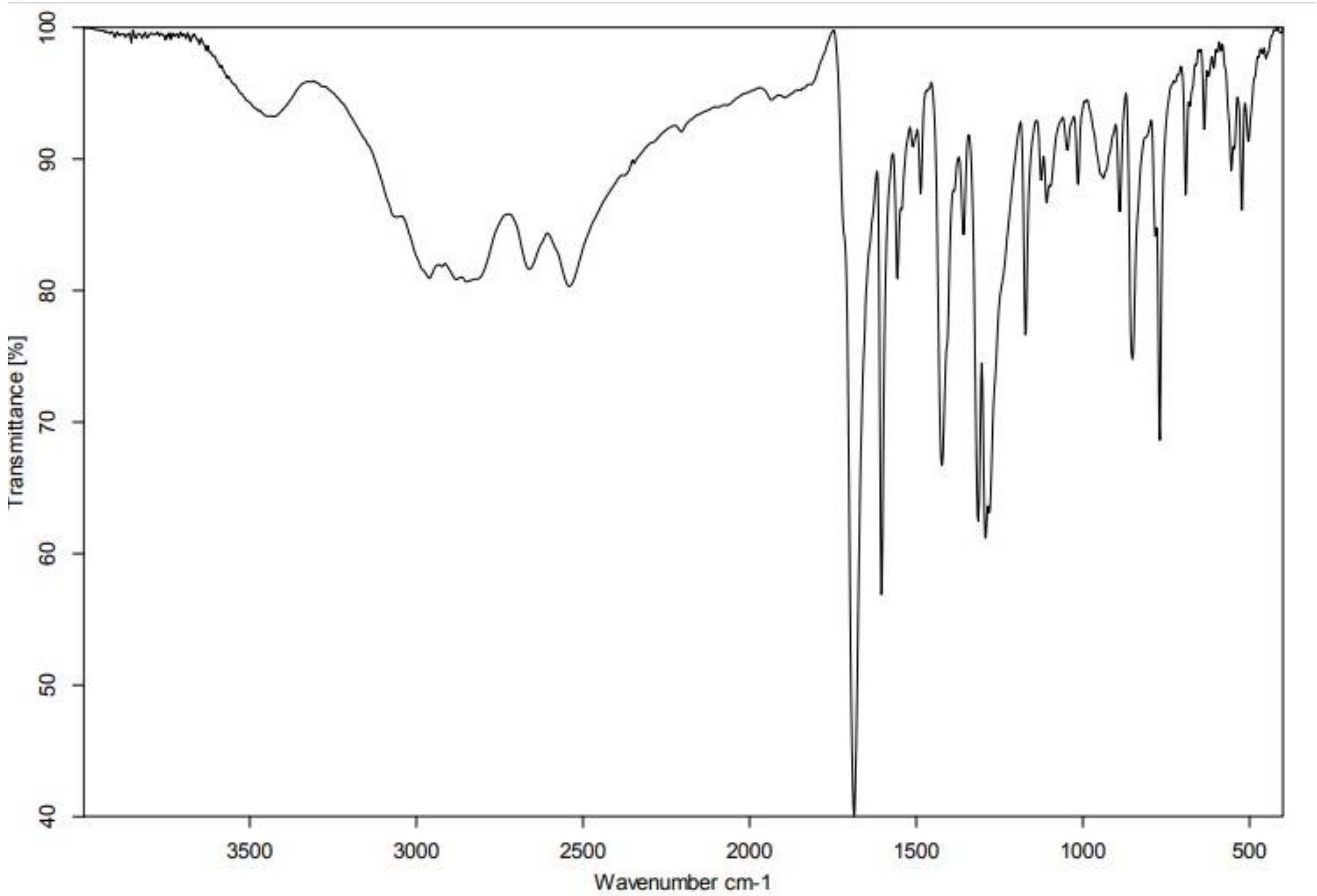

Figure S6. IR spectrum (ATR mode) of BTD **2**.

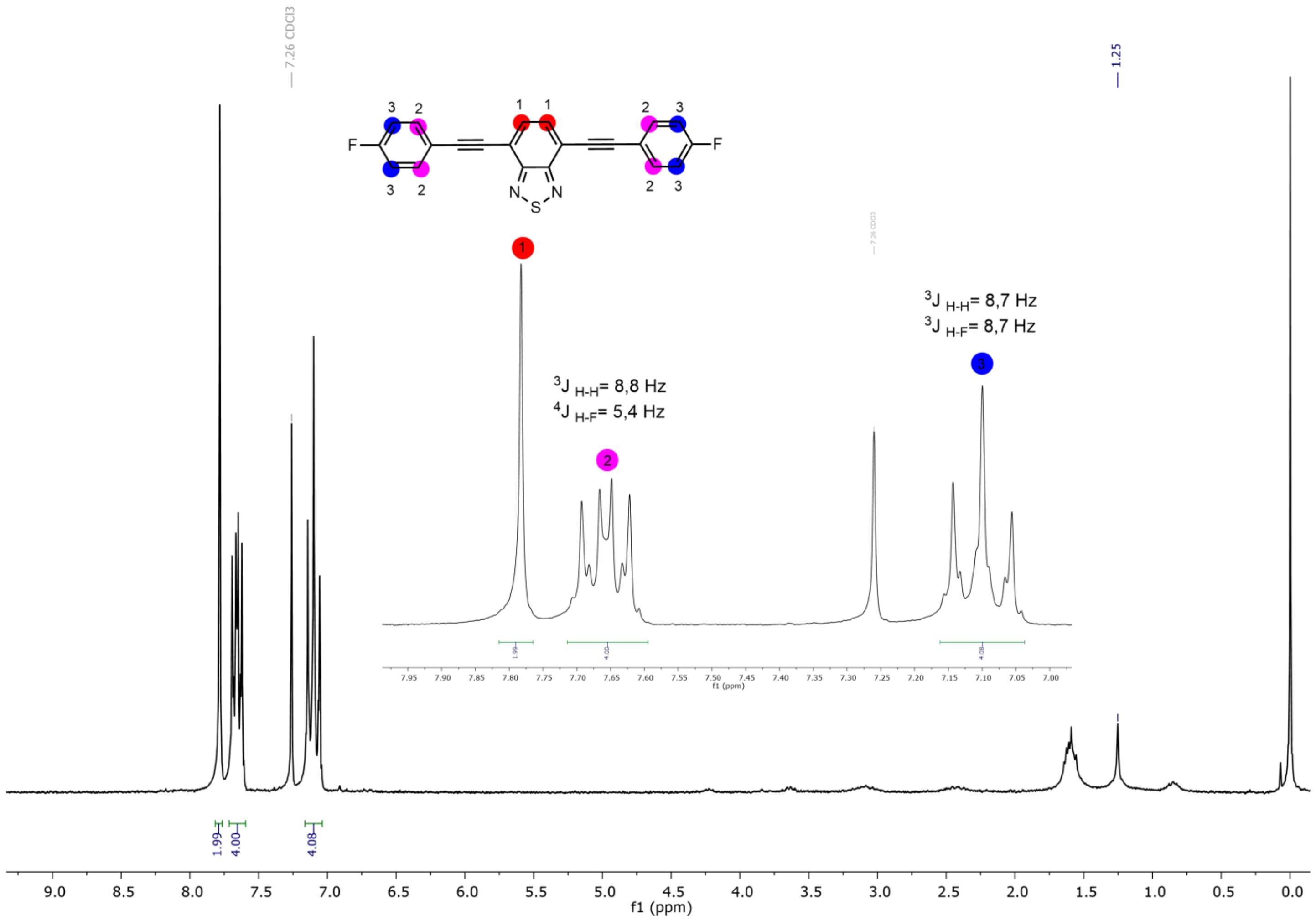

Figure S7. ^{1}H NMR spectrum of BTD **3** in $CDCl_3$ (400 MHz).

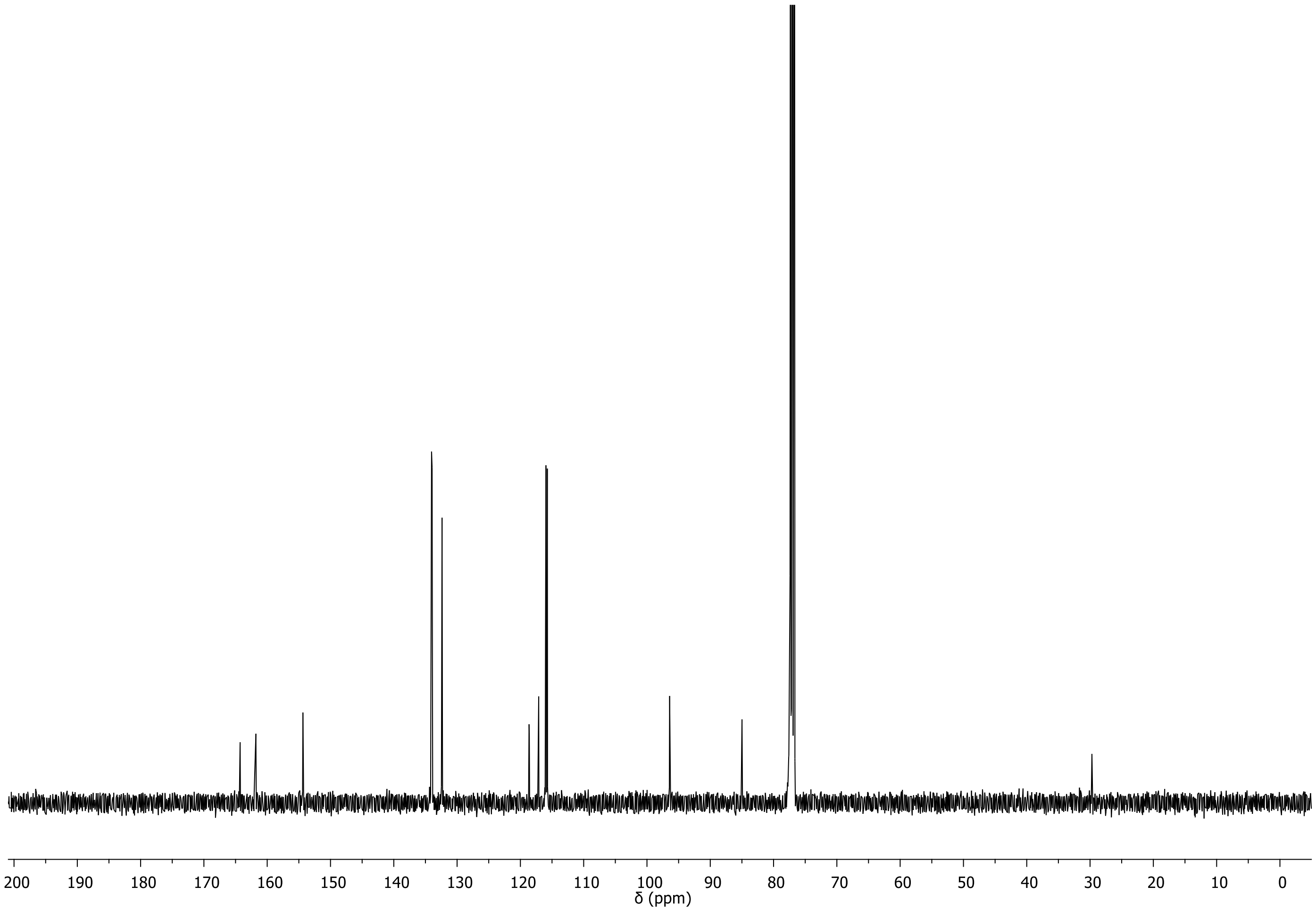

Figure S8. ^{13}C NMR spectrum of BTD **3** in $CDCl_3$ (100 MHz).

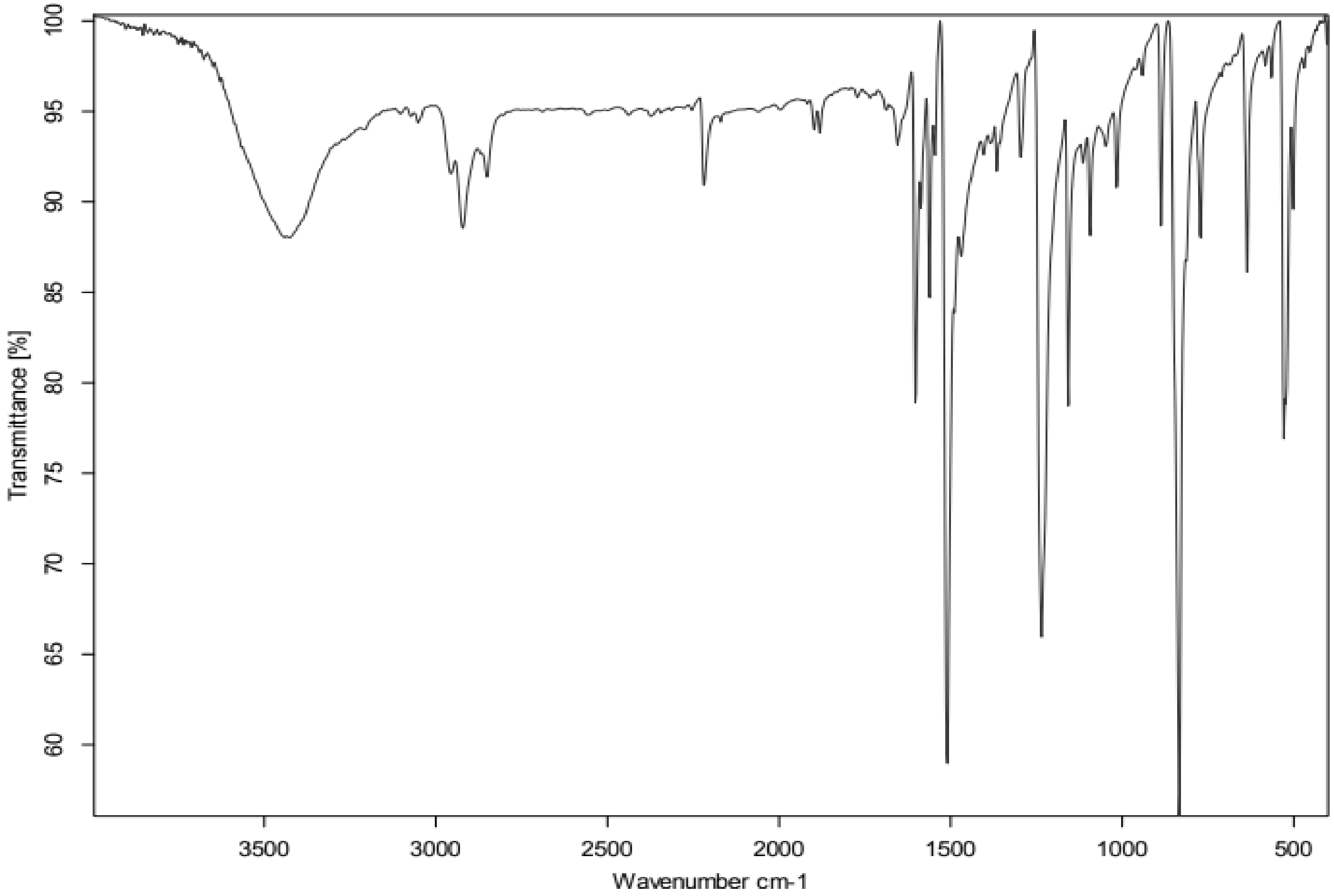

Figure S9. IR spectrum (ATR mode) of BTD **3**.

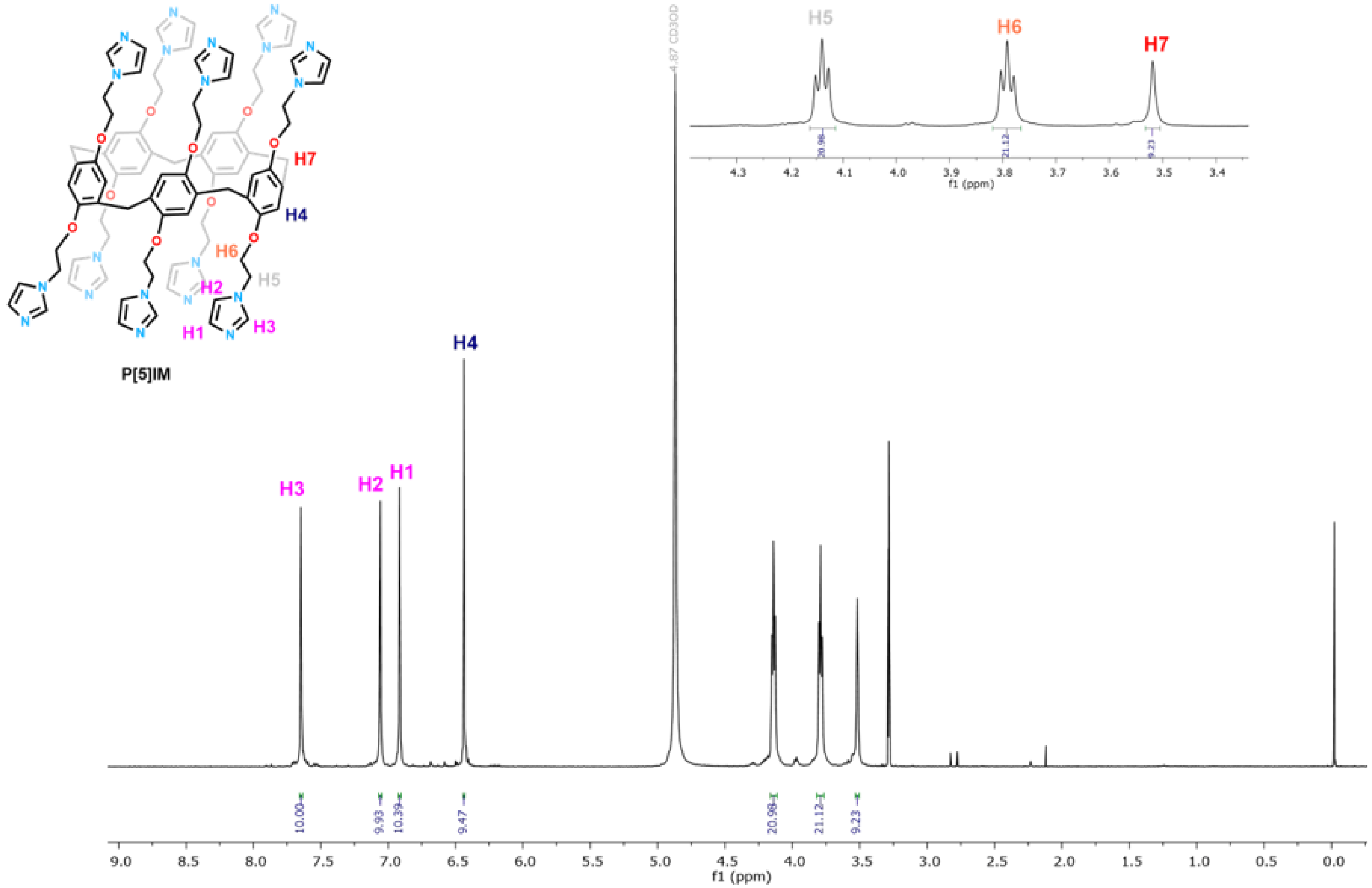

Figure S10. ^{1}H NMR spectrum of P[5]Im in CD_3OD (400 MHz).

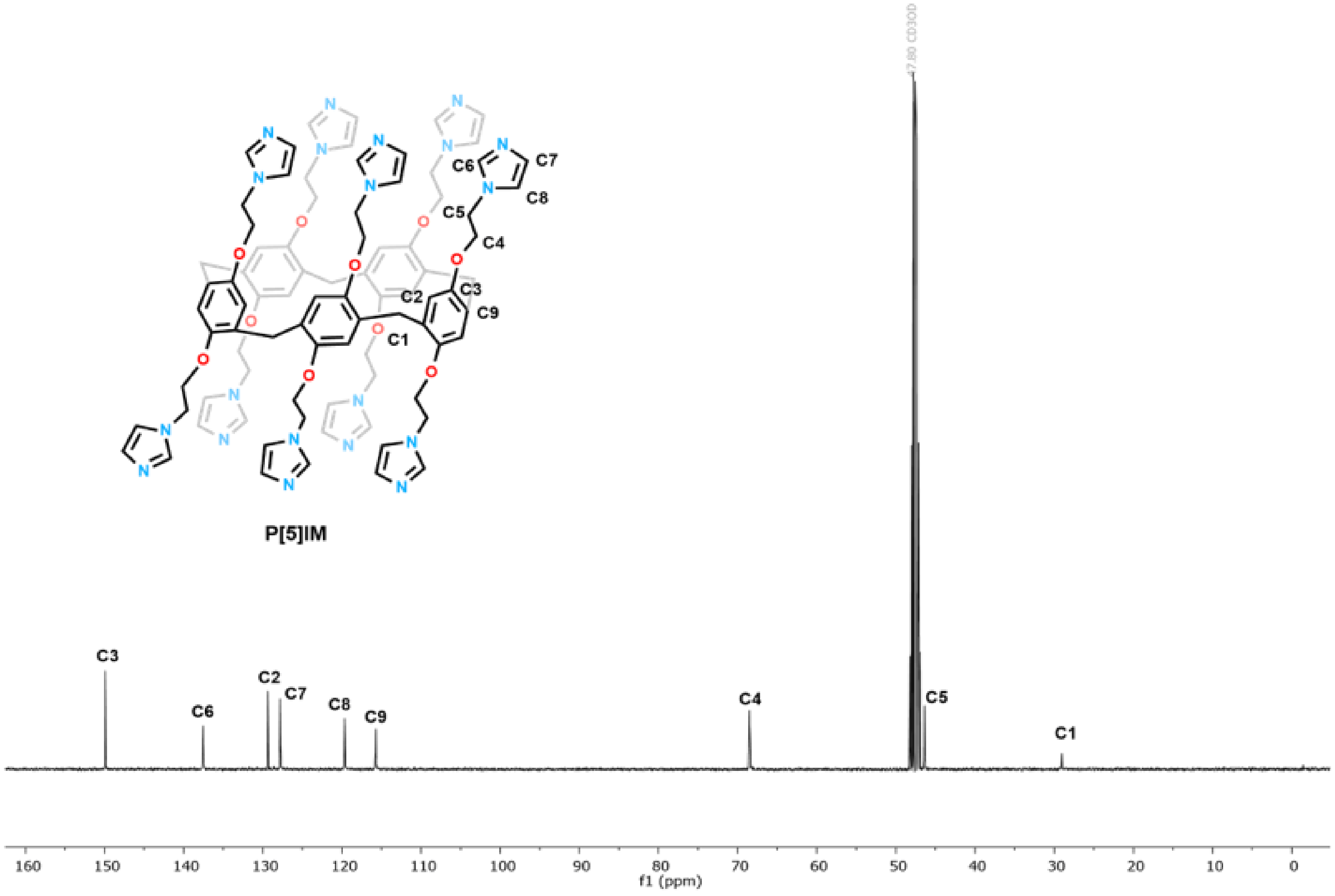

Figure S11. ^{13}C NMR spectrum of P[5]Im in CD_3OD (100 MHz).

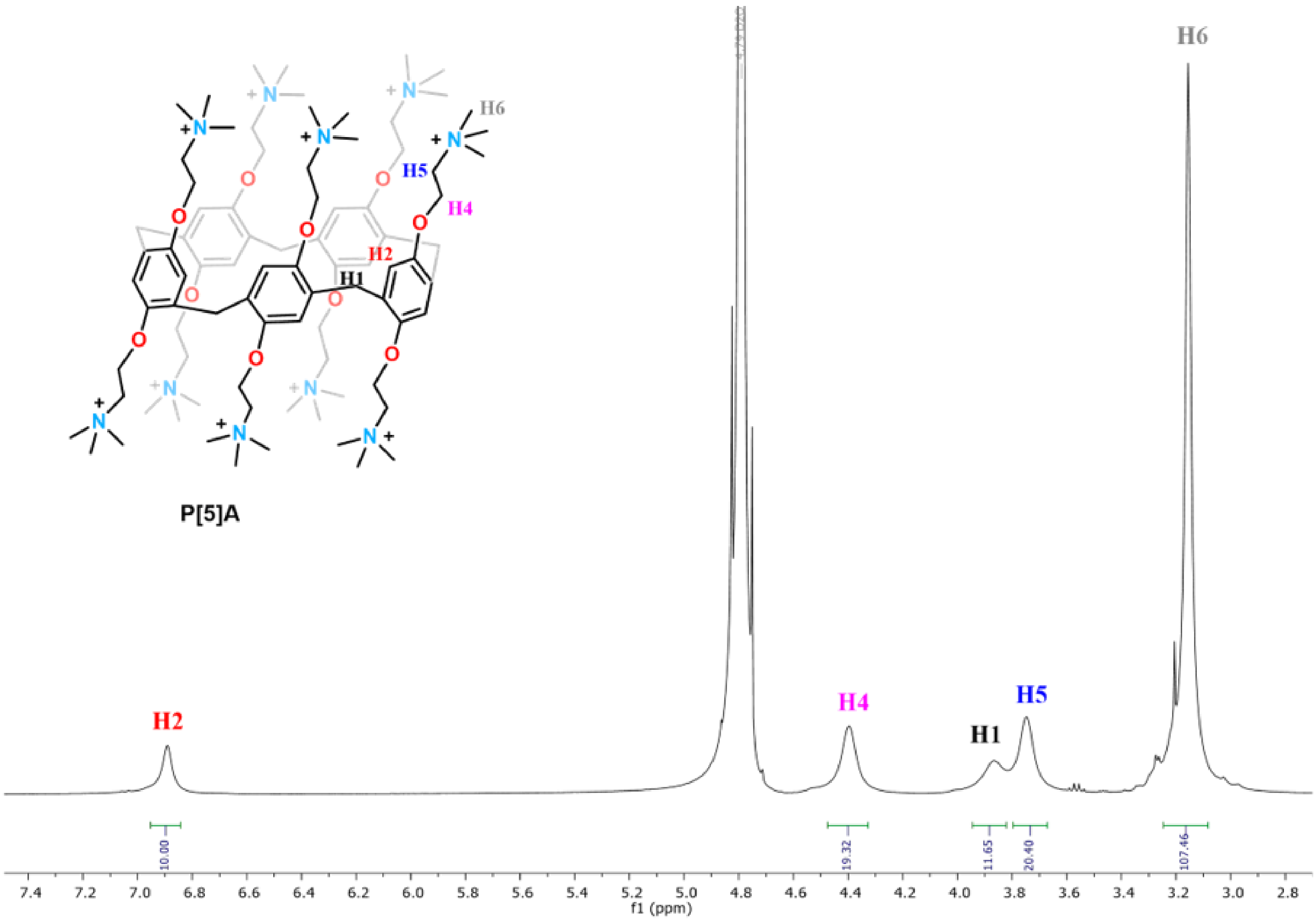

Figure S12. ^{1}H NMR spectrum of P[5]A in D_2O (400 MHz).

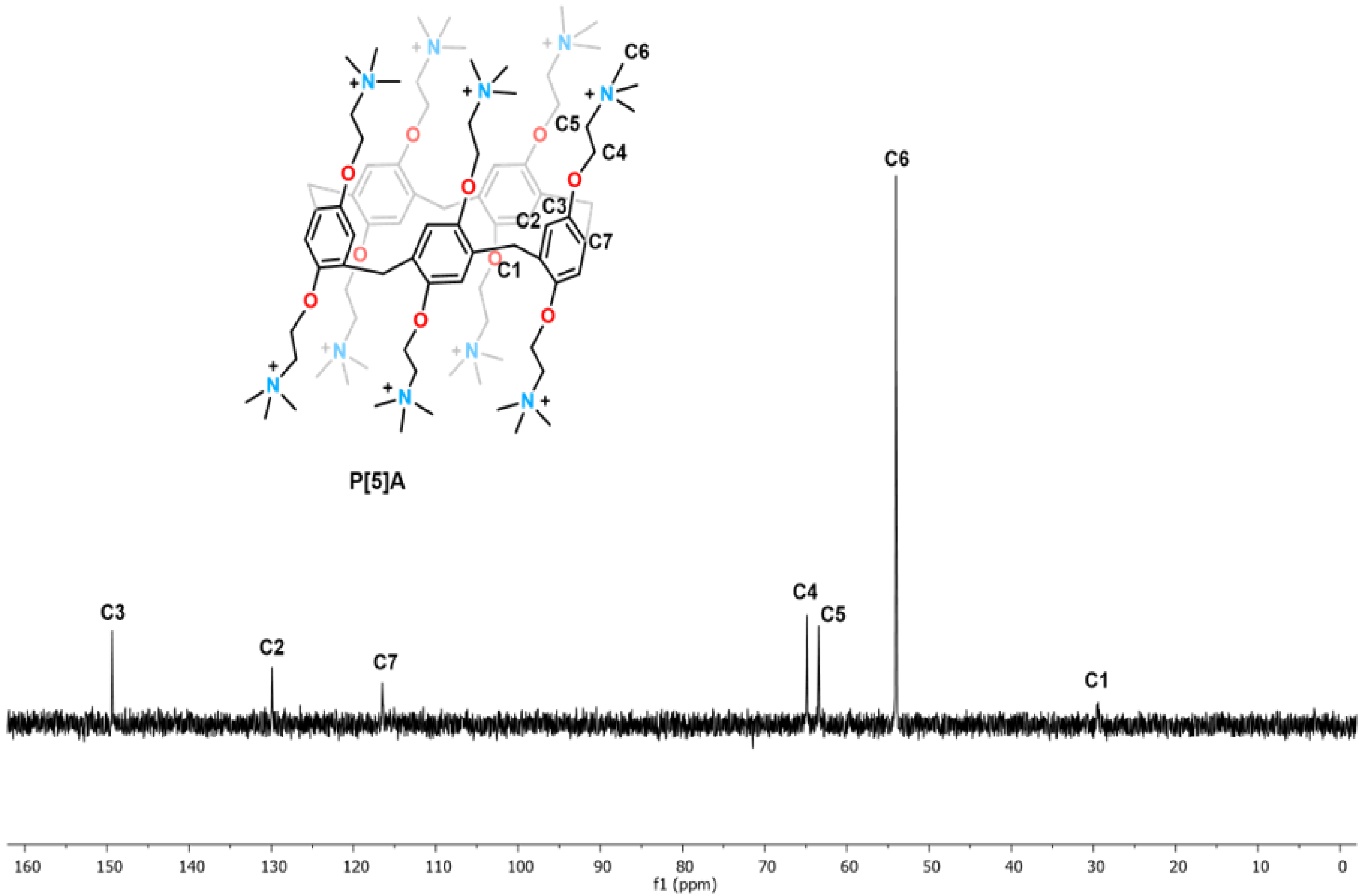

Figure S13. ^{13}C NMR spectrum of P[5]A **6** in D_2O (100 MHz).

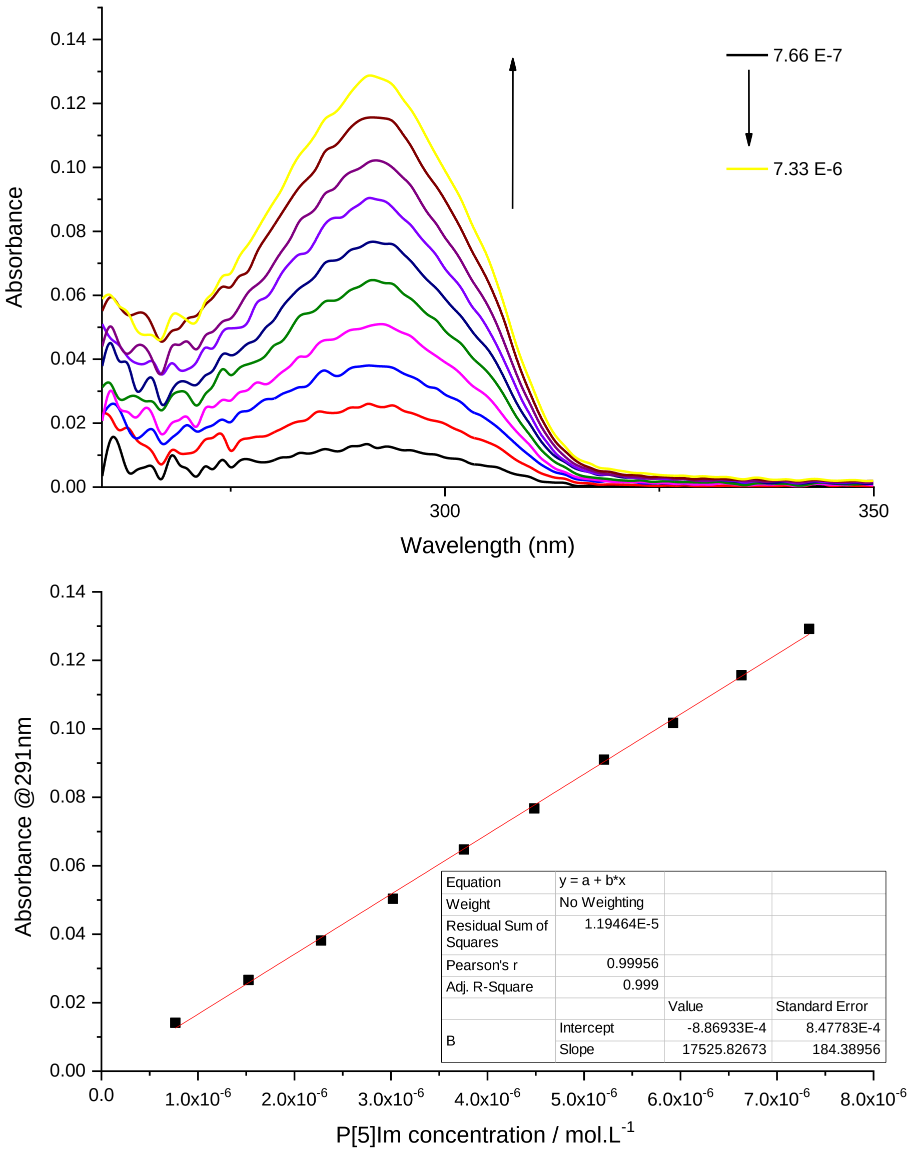

Figure S14. UV-Vis absorption spectra of P[5]Im and respective calibration curve in ethanol at concentrations ranging 10^{-7}- 10^{-6} M.

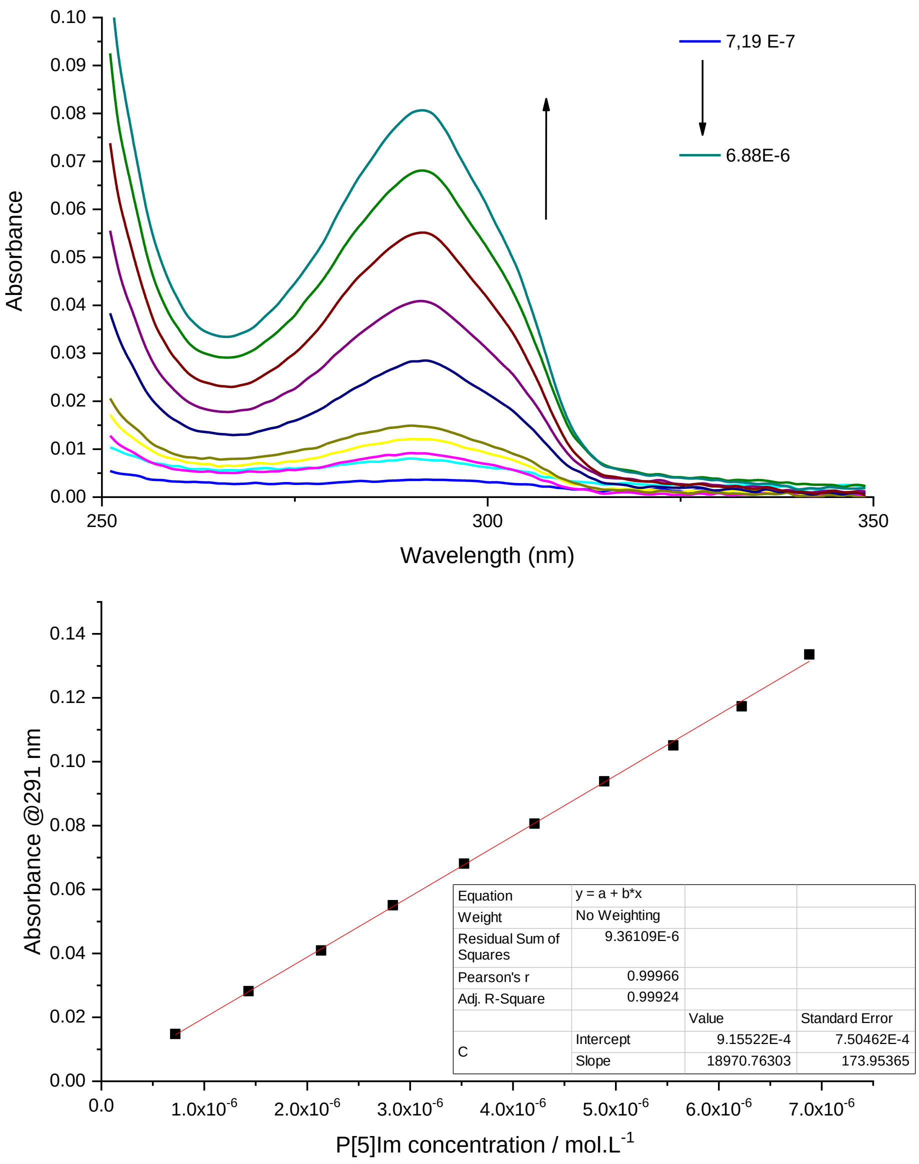

Figure S15. UV-Vis absorption spectra of P[5]Im and respective calibration curves in methanol at concentrations ranging 10^{-7}- 10^{-6} M.

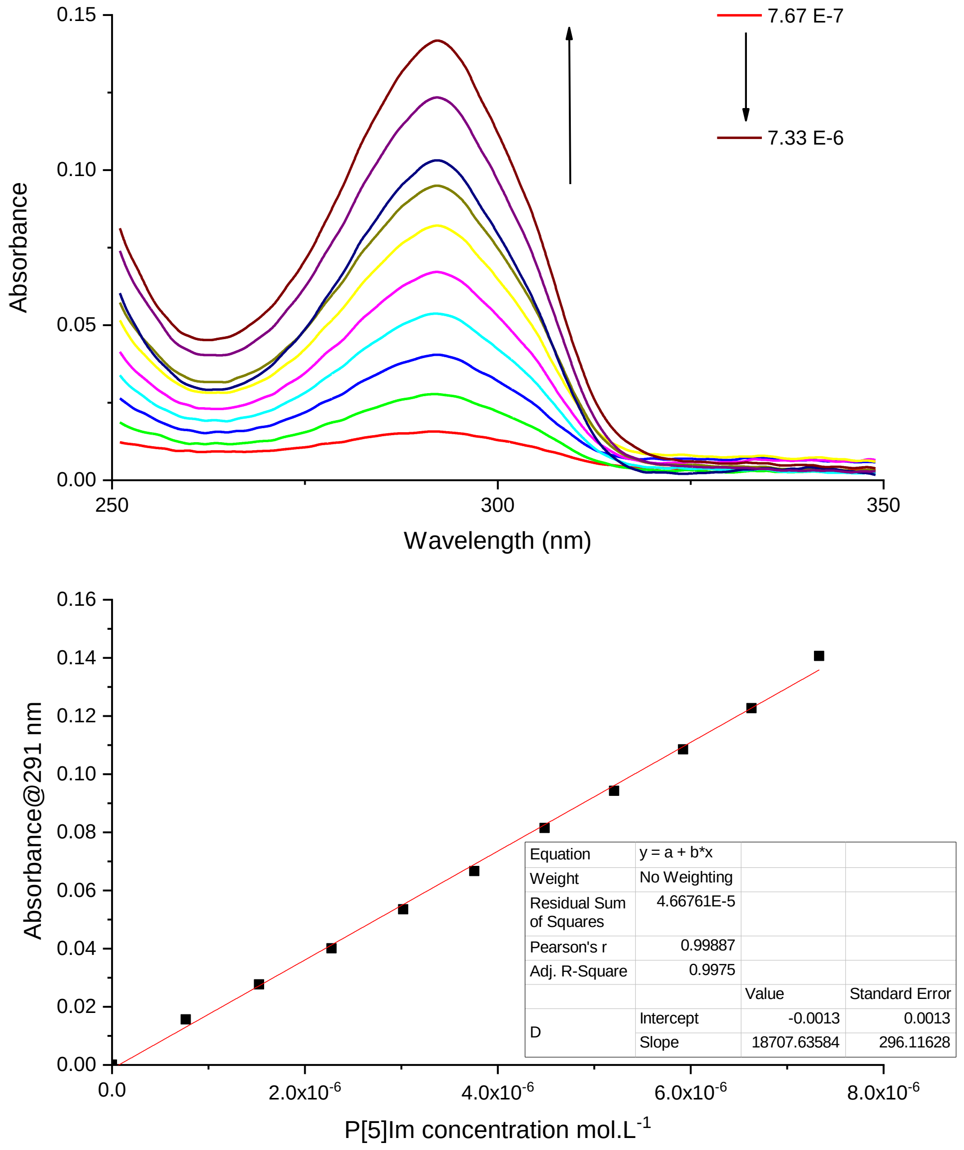

Figure S16. UV-Vis absorption spectra of P[5]Im and respective calibration curves in 20/80 ethanol-water at concentrations ranging 10^{-7}- 10^{-6} M.

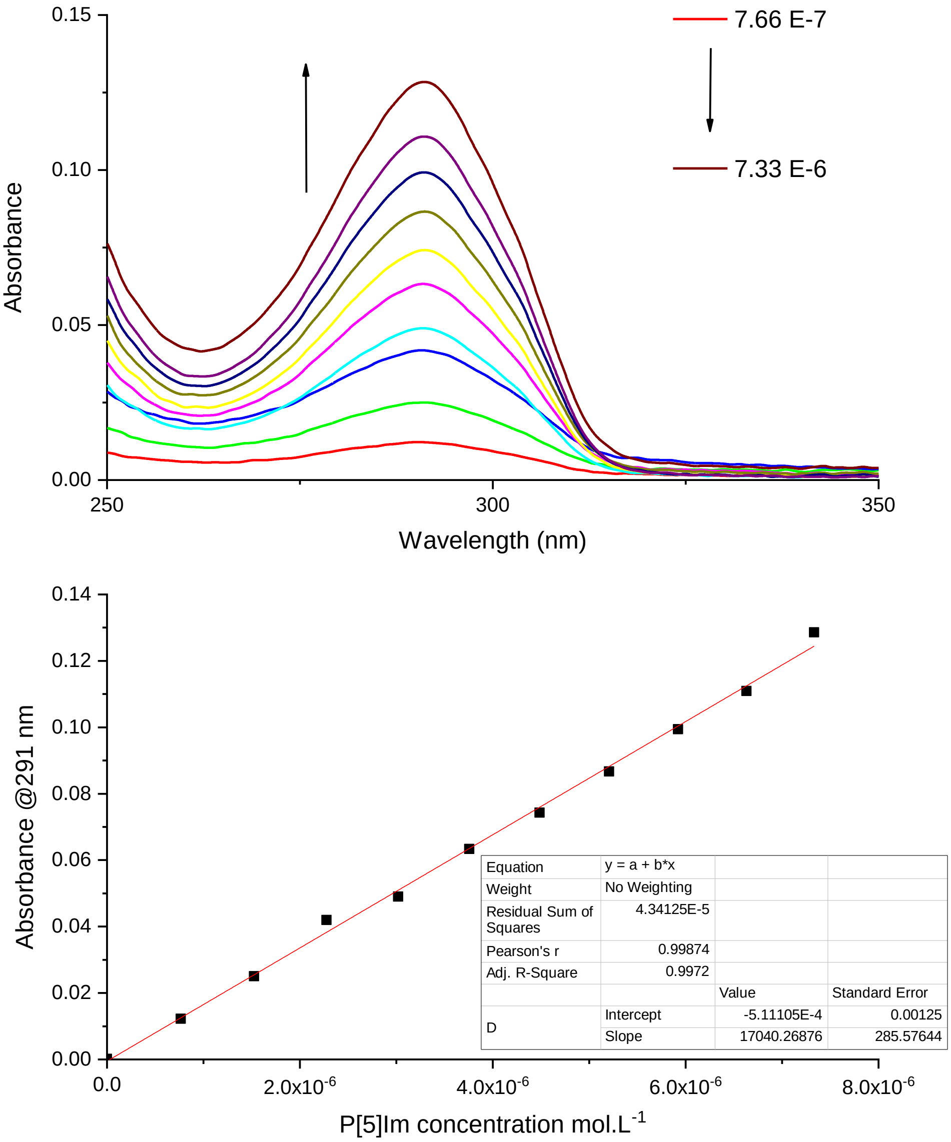

Figure S17. UV-Vis absorption spectra of P[5]Im and respective calibration curves in 30/70 ethanol-water (right) at concentrations ranging 10^{-7}- 10^{-6} M.

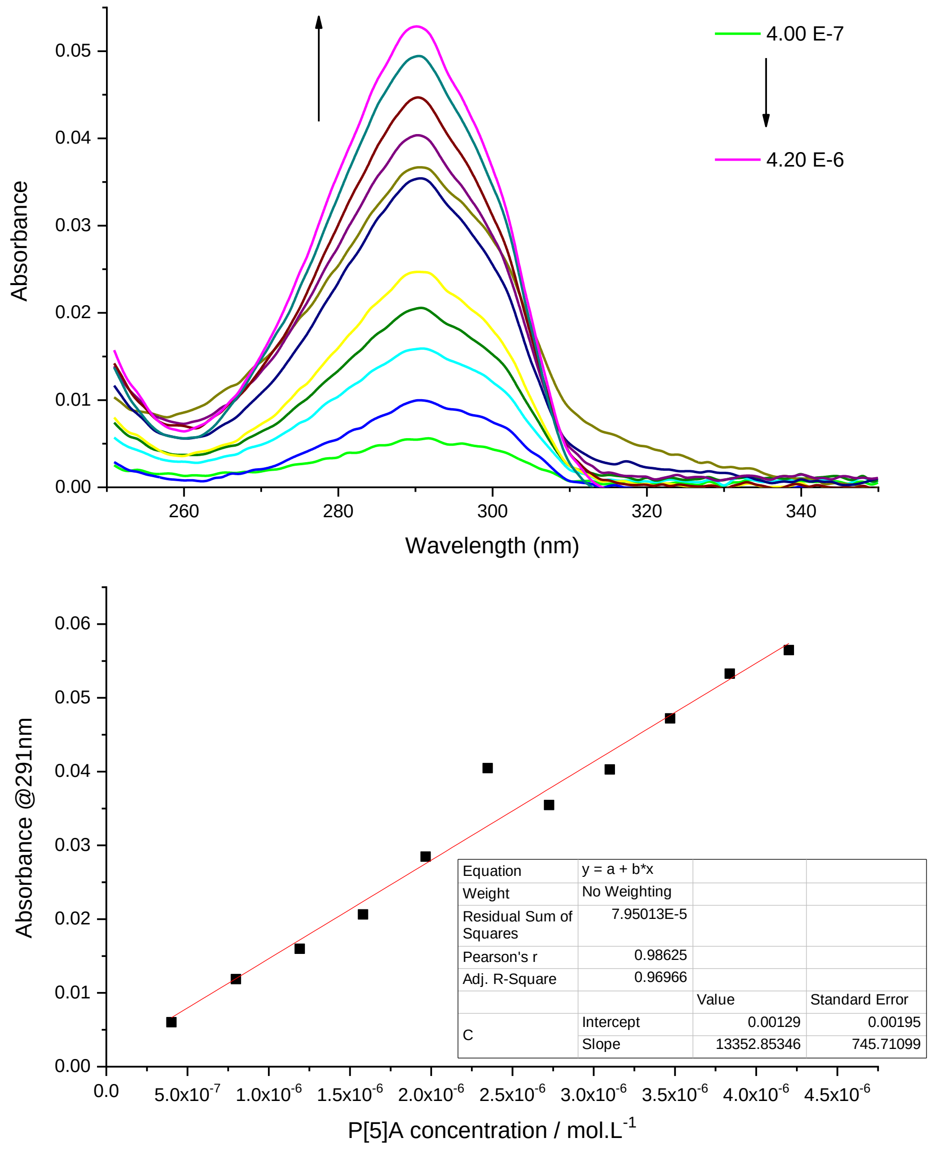

Figure S18. UV-Vis absorption spectra of P[5]A and respective calibration curve in water at concentrations ranging 10^{-7}- 10^{-6} M.

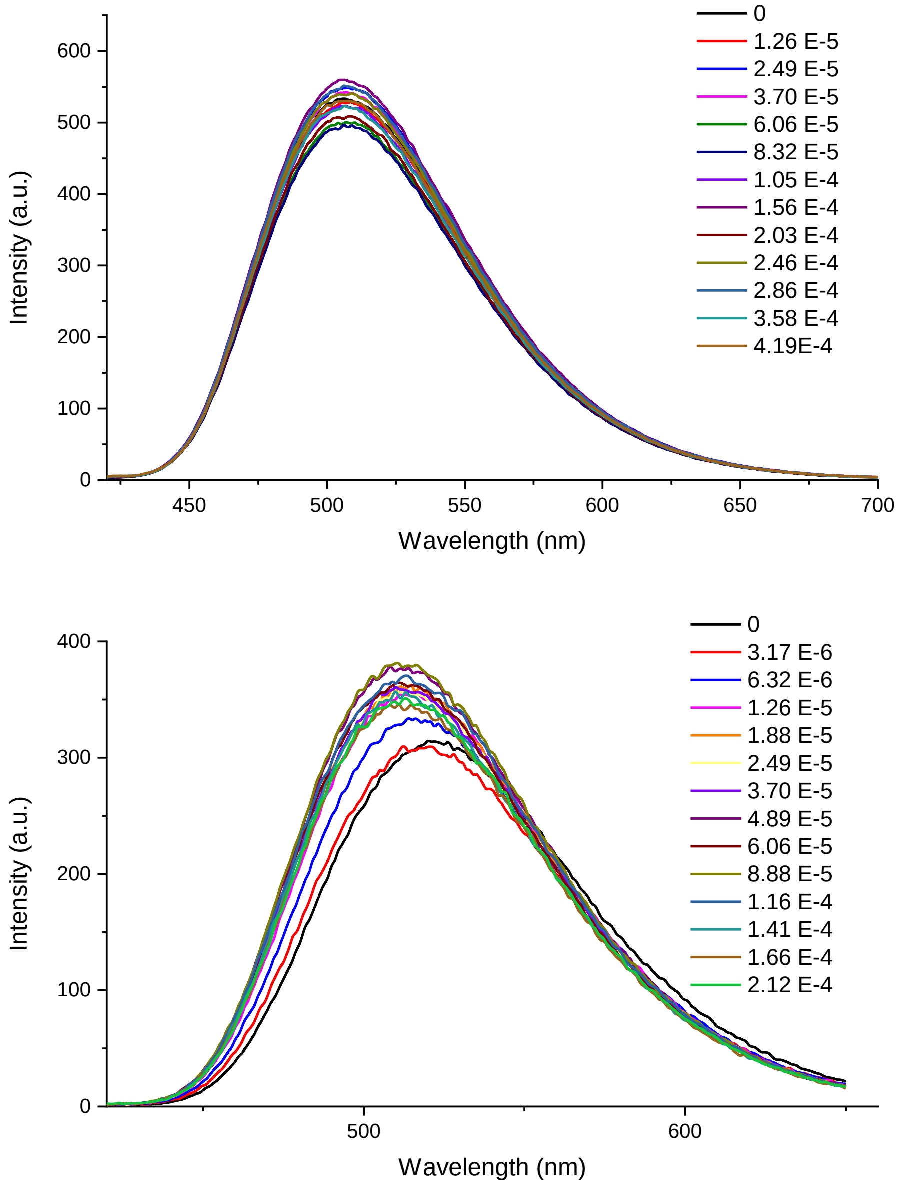

Figure S19. Spectrofluorimetric titration of BTD **1** (top) and BTD **2** (bottom) with different concentrations of P[5]Im in ethanol.

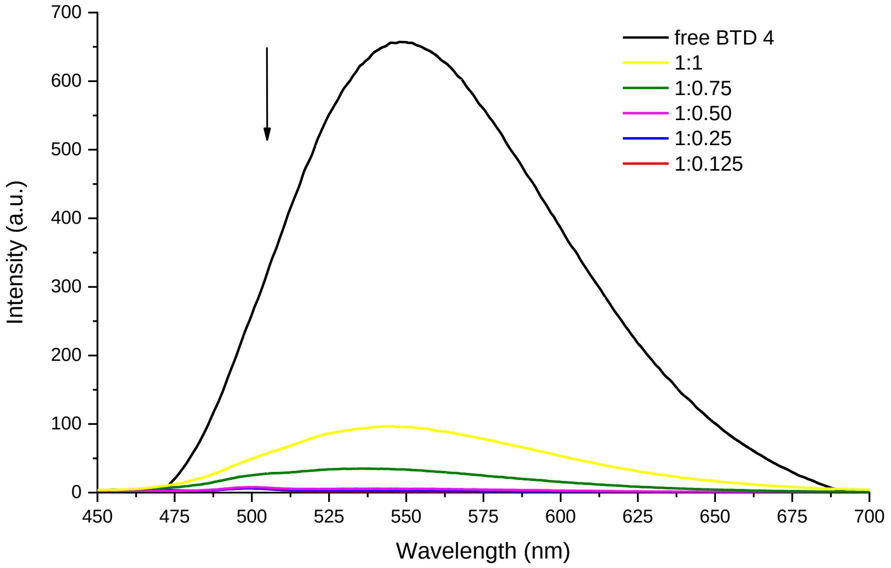

Figure S20. Fluorescence emission spectra for anionic BTD **4** and different mixtures of cationic P[5]A in water (10^{-5} M, λ_{exc}=428 nm, excitation/emission slits: 10.0 nm/10.0 nm).

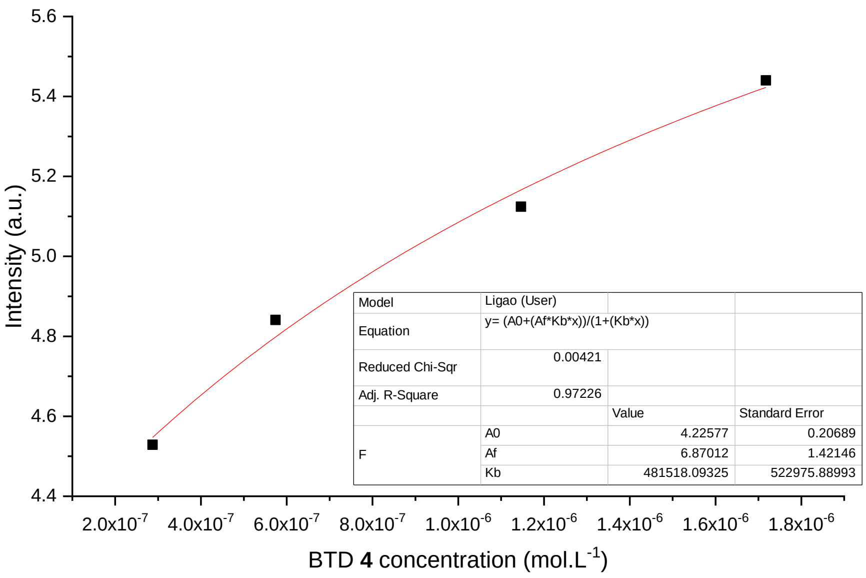

Figure S21. Fluorescence emission intensity for different mixtures of BTD **4**/P[5]A as a function of BTD concentrations at 10^{-6} M (λ_{em}= 423 nm, excitation/emission slits: 5.0 nm/5.0 nm).

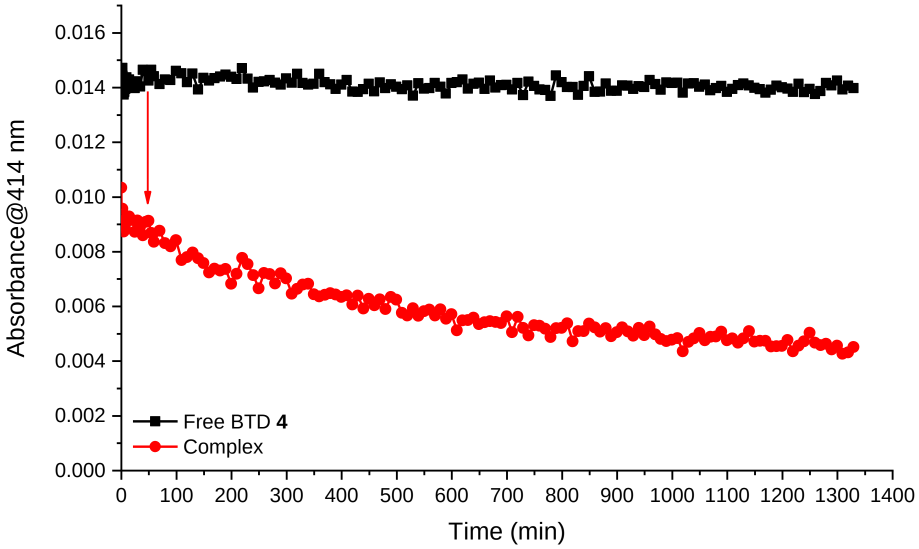

Figure S22. Successive UV-Vis absorption spectra of the 1:1 mixture of BTD **4**/P[5]A at 10^{-6} M monitored by 17 hours (left) and absorbance intensity at 291 nm (black) and 414 nm (red) versus time.

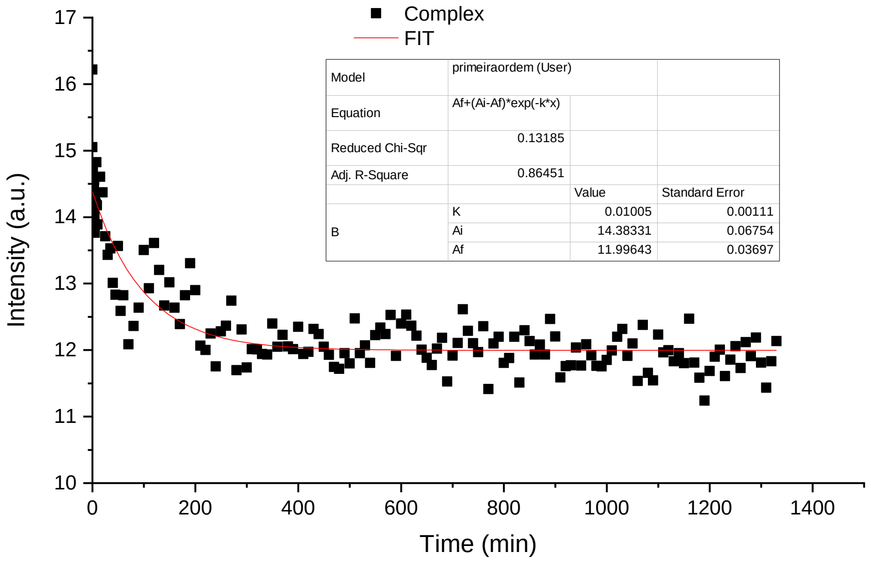

Figure S23. Fluorescence emission intensity for the 1:1 mixture of BTD **4**/P[5]A at10^{-6} M monitored by 17 hours (excitation/emission slits: 10.0 nm/10.0 nm).

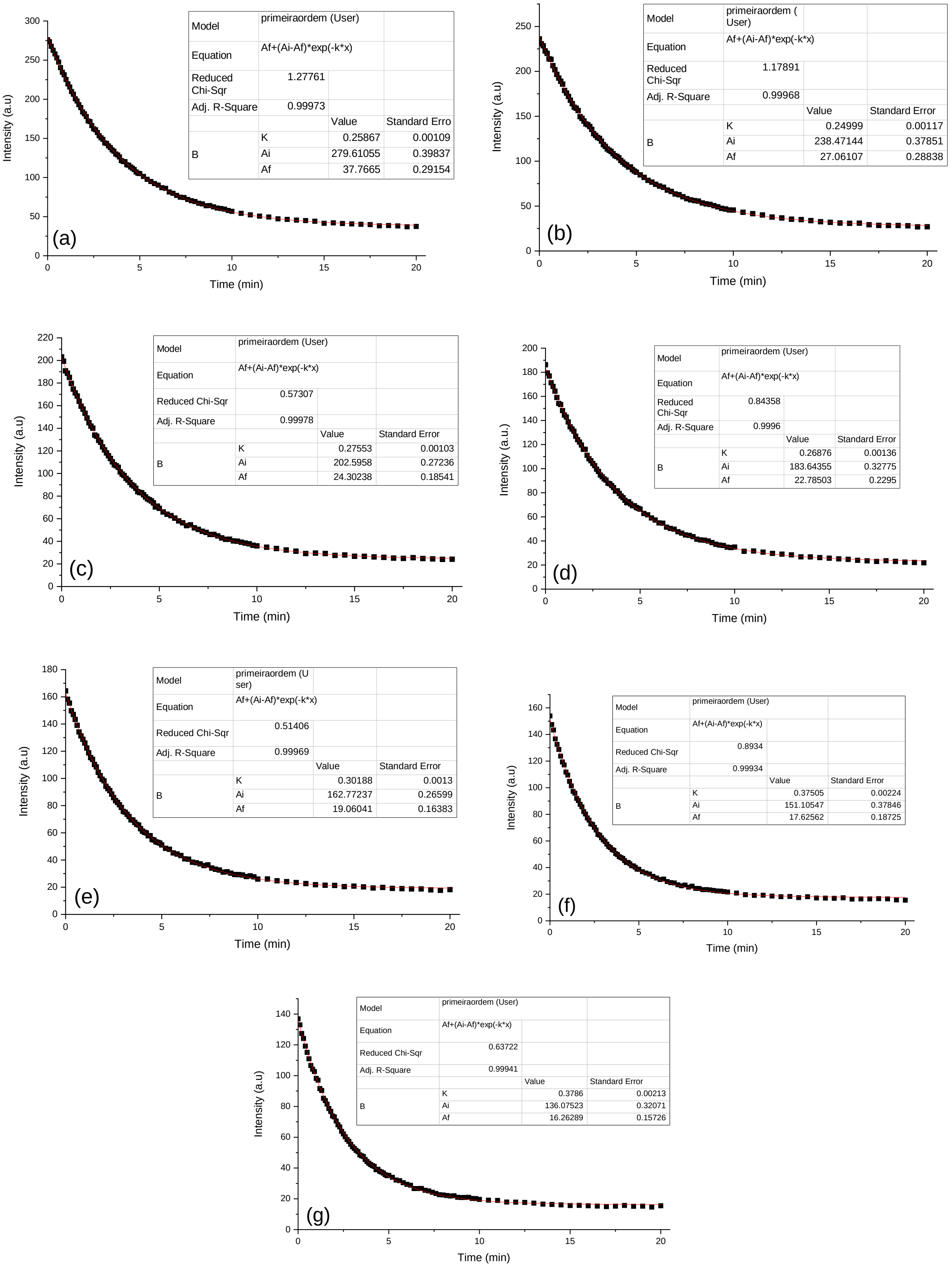

Figure S24. Fluorescence emission intensities of mixtures of 1:1 BTD **4**/P[5]A in different concentrations: (a) 4.98×10^{-6} M, (b) 6.52×10^{-6} M, (c) 8.00×10^{-6} M, (d) 9.44×10^{-6} M, (e) 1.08×10^{-5} M, (f) 1.15×10^{-5} M, and (g) 1.28×10^{-5} M (excitation/emission slits: 10.0 nm/10.0 nm).

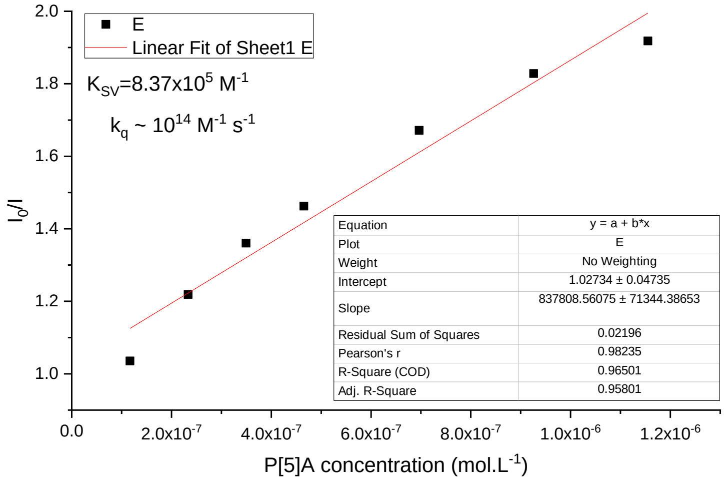

Figure S25. Stern-Volmer plot of fluorescence quenching of BTD **4** in the presence of different concentrations of P[5]A.

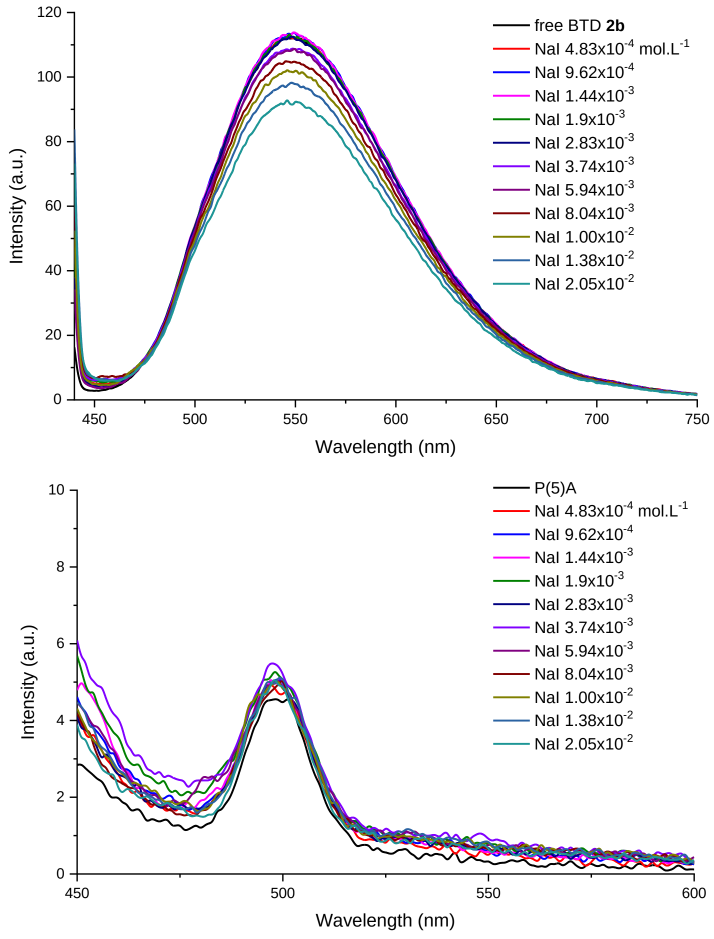

Figure S26. Spectrofluorimetric titration of NaI in the presence of (top) pure BTD **2b** and (bottom) pure P[5]A in water (10^{-6} M) slits 10/10 (Exc./Em.).

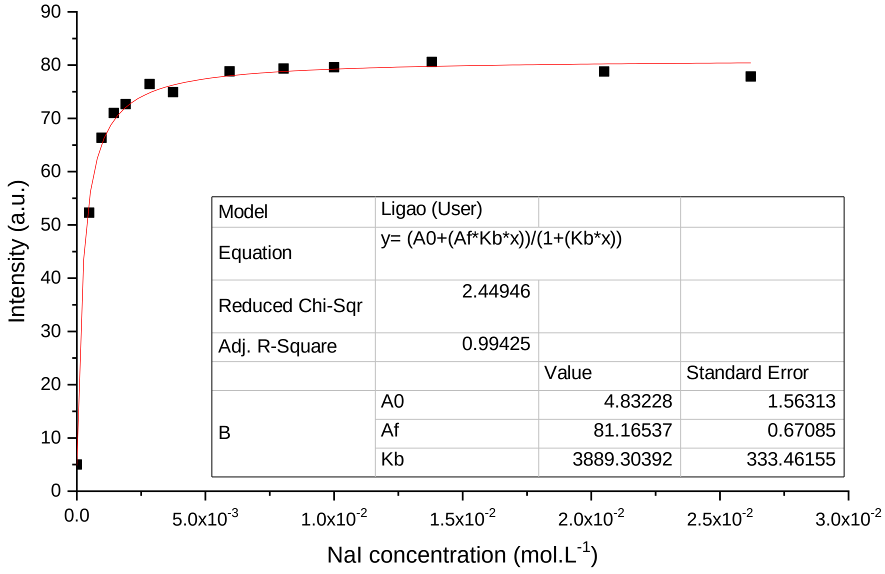

Figure S27. Non-linear fitting for K_b determination between BTD **4**⊂P[5]A and NaI.

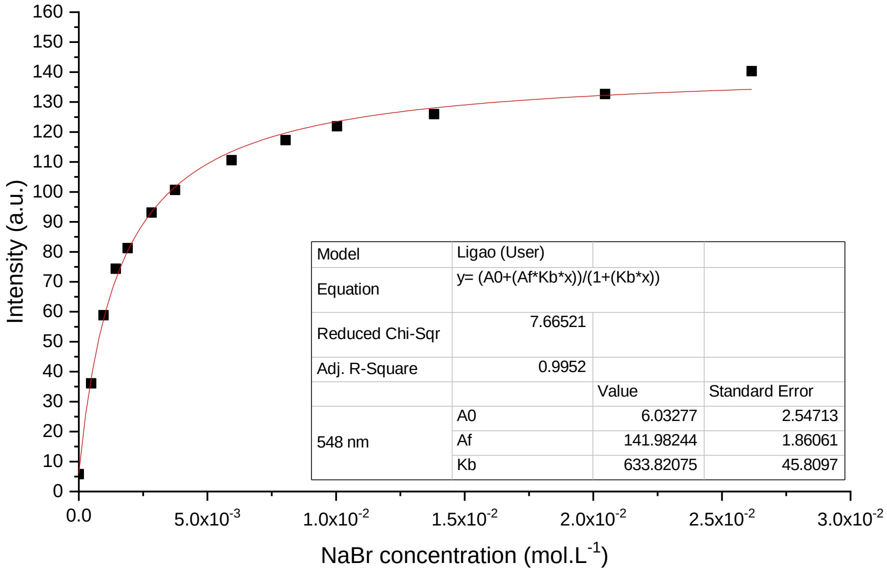

Figure S28. Non-linear fitting for K_b determination between BTD **4**⊂P[5]A and NaBr.

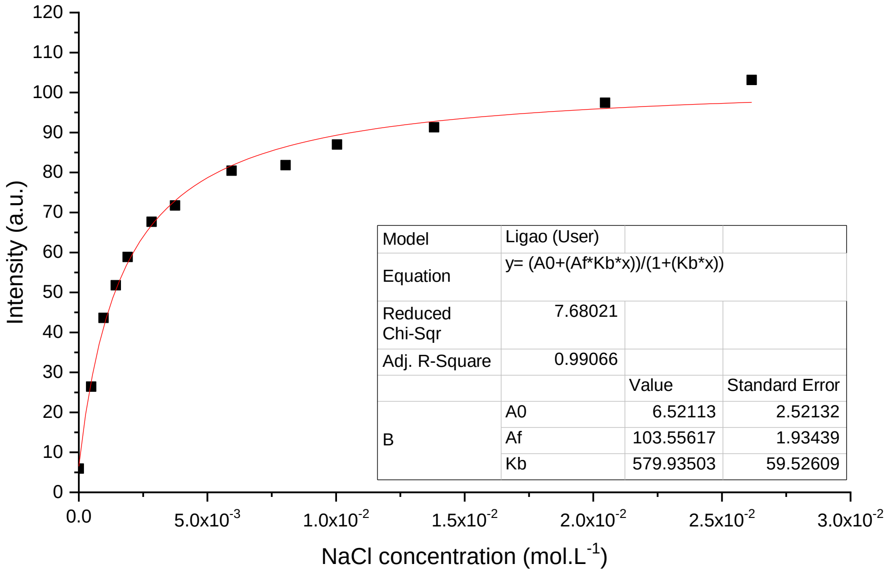

Figure S29. Non-linear fitting for K_b determination between BTD **4**⊂P[5]A and NaCl.

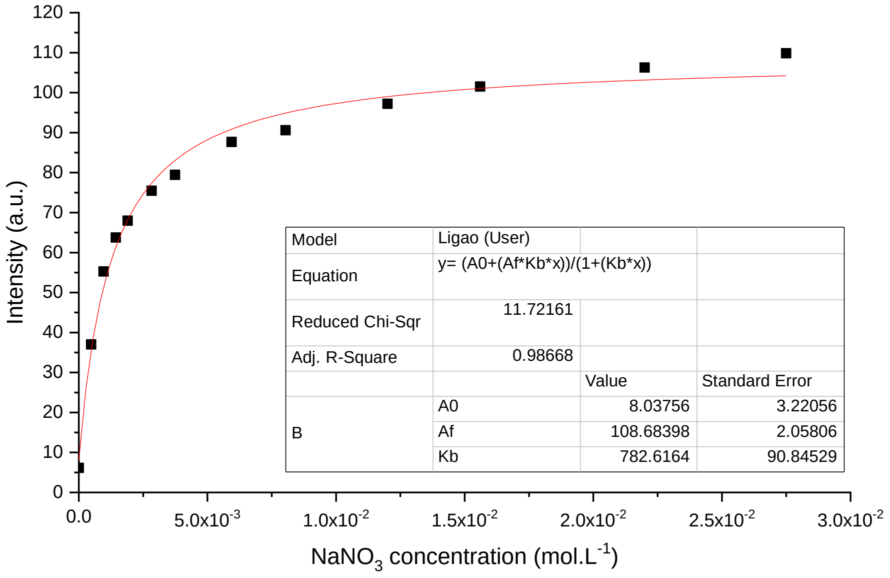

Figure S30. Non-linear fitting for K_b determination between BTD **4**⊂P[5]A and $NaNO_3$.

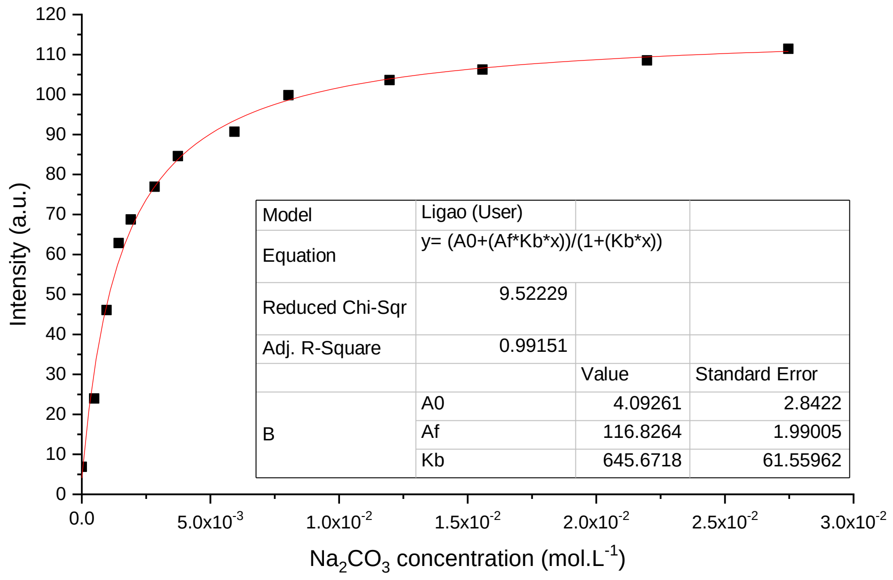

Figure S31. Non-linear fitting for K_b determination between BTD **4**⊂P[5]A and Na_2CO_3.

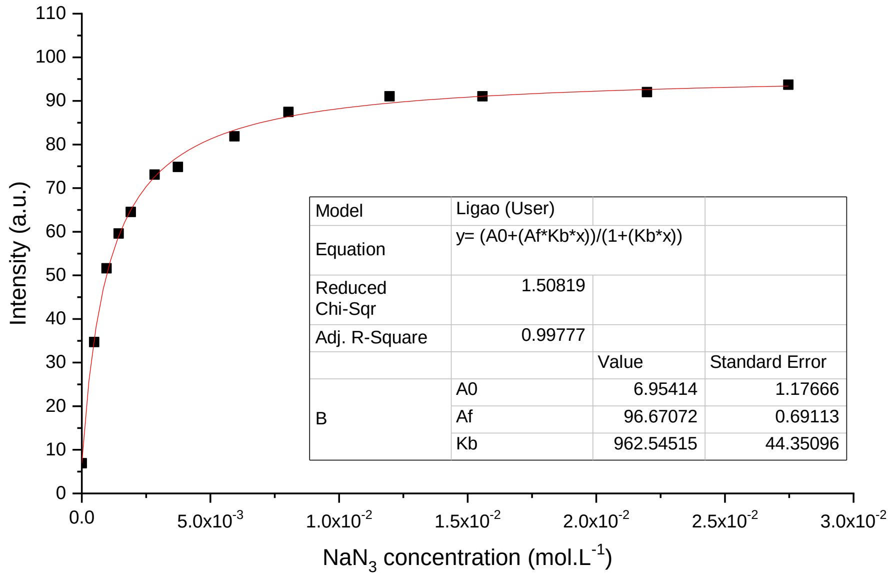

Figure S32. Non-linear fitting for K_b determination between BTD **4**⊂P[5]A and NaN_3.

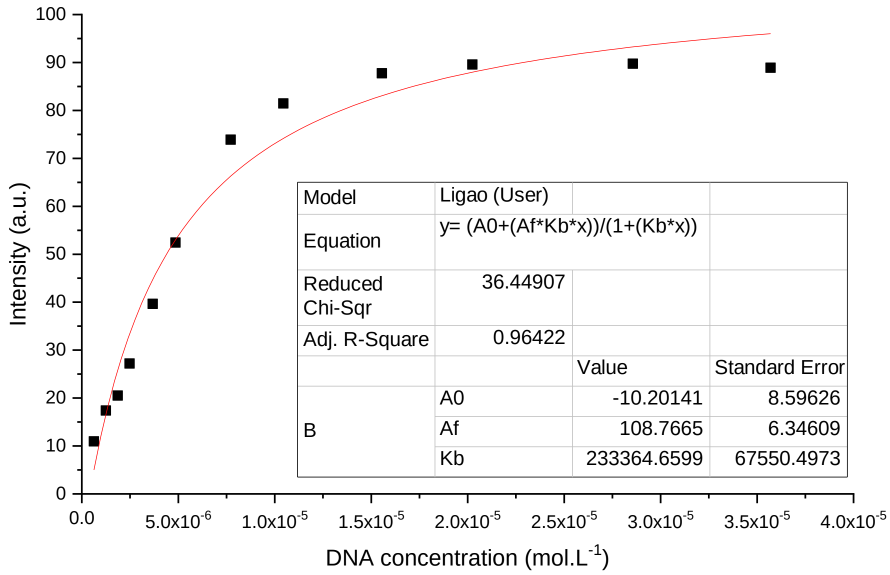

Figure S33. Non-linear fitting for K_b determination between BTD **4**⊂P[5]A and ct-DNA.

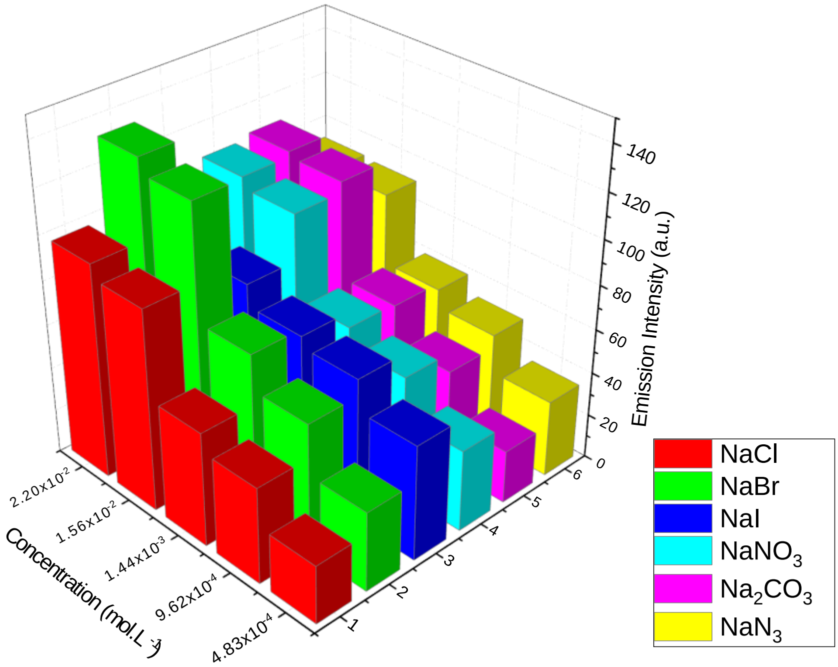

Figure S34. Comparative histogram showing the relative fluorescence intensities of BTD **4**⊂P[5]A in the presence of various anions (1: NaCl, 2: NaBr, 3: NaI, 4: $NaNO_3$, 5: Na_2CO_3, and 6: NaN_3).

Table S1. HOMOLUMO gaps (eV) at S_0 and S_1 minima by solvent (CPCM), where Δ=S_1-S_0.

BTD	Solvent	S_0 gap	S_1 gap	Δ
**1**	Dichloromethane	6.994	6.100	−0.894
	1,4-Dioxane	6.973	6.127	−0.846
	EtOH	6.998	6.092	−0.906
	THF	6.992	6.101	−0.891
	Water	7.010	6.090	−0.919
**2**	Dichloromethane	6.998	6.109	−0.889
	1,4-Dioxane	6.982	6.138	−0.844
	EtOH	7.002	6.103	−0.900
	THF	6.997	6.112	−0.885
	Water	7.008	6.100	−0.908
**3**	Dichloromethane	6.980	6.094	−0.886
	1,4-Dioxane	6.967	6.130	−0.837
	EtOH	6.982	6.085	−0.897
	THF	6.979	6.097	−0.882
	Water	6.983	6.082	−0.901
**4**	Dichloromethane	6.940	6.059	−0.881
	1,4-Dioxane	6.891	6.062	−0.829
	EtOH	6.952	6.058	−0.894
	THF	6.937	6.059	−0.878
	Water	6.956	6.058	−0.899
